# Supplementary material for: Characterizing and inferring quantitative cell cycle phase in single-cell RNA-seq data analysis
Source: Genome Res. 2020 Apr;30(4):611–21. doi: 10.1101/gr.247759.118 (PMC7197478; doi:10.1101/gr.247759.118)
Supplement: Supplemental Material [file supp_30_4_611__index.html]

Characterizing and inferring quantitative cell cycle phase in single-cell RNA-seq data analysis — Characterizing and inferring quantitative cell cycle phase in single-cell RNA-seq data analysis — Supplemental Material 

# Characterizing and inferring quantitative cell cycle phase in single-cell RNA-seq data analysis

## Supplemental Material

- Supplemental\_File\_S1\_.xlsx
- Supplemental\_File\_S2\_.xlsx
- Supplemental\_Material.pdf
- Supplemental\_peco-paper-master-source-code.tar.gz
