# Supplementary material for: Characterizing and inferring quantitative cell cycle phase in single-cell RNA-seq data analysis
Source: Genome Res. 2020 Apr;30(4):611–21. doi: 10.1101/gr.247759.118 (PMC7197478; doi:10.1101/gr.247759.118)
Supplement: Supplemental Material [file supp_gr.247759.118_Supplemental_peco-paper-master-source-code.tar.gz › peco-paper-master/docs/predict_thinned_data.html]

Comparison of performances on our data


peco-paper

- Home
- About
- License

- Source code


# Comparison of performances on our data

workflowr

- Summary
- Checks
- Past versions

**Last updated:** 2020-01-25

**Checks:**  7  0

**Knit directory:** `peco-paper/`

This reproducible R Markdown analysis was created with workflowr (version 1.6.0). The *Checks* tab describes the reproducibility checks that were applied when the results were created. The *Past versions* tab lists the development history.

---

**R Markdown file:** up-to-date

Great! Since the R Markdown file has been committed to the Git repository, you know the exact version of the code that produced these results.

**Environment:** empty

Great job! The global environment was empty. Objects defined in the global environment can affect the analysis in your R Markdown file in unknown ways. For reproduciblity it’s best to always run the code in an empty environment.

**Seed:** `set.seed(20190814)`

The command `set.seed(20190814)` was run prior to running the code in the R Markdown file. Setting a seed ensures that any results that rely on randomness, e.g. subsampling or permutations, are reproducible.

**Session information:** recorded

Great job! Recording the operating system, R version, and package versions is critical for reproducibility.

**Cache:** none

Nice! There were no cached chunks for this analysis, so you can be confident that you successfully produced the results during this run.

**File paths:** relative

Great job! Using relative paths to the files within your workflowr project makes it easier to run your code on other machines.

**Repository version:** fdebe2b

Great! You are using Git for version control. Tracking code development and connecting the code version to the results is critical for reproducibility. The version displayed above was the version of the Git repository at the time these results were generated.   
  
 Note that you need to be careful to ensure that all relevant files for the analysis have been committed to Git prior to generating the results (you can use `wflow_publish` or `wflow_git_commit`). workflowr only checks the R Markdown file, but you know if there are other scripts or data files that it depends on. Below is the status of the Git repository when the results were generated:

```
Ignored files:
    Ignored:    .Rhistory
    Ignored:    .Rproj.user/

Untracked files:
    Untracked:  analysis/npreg_trendfilter_quantile.Rmd
    Untracked:  code/suppfig09_?.R
    Untracked:  data/HumanLengESC.rds
    Untracked:  data/data_training_test/
    Untracked:  data/eset-filtered.rds
    Untracked:  data/fit.quant.rds
    Untracked:  data/fit.trend.perm.lowmiss.rds
    Untracked:  data/fit_diff_cyclone.rds
    Untracked:  data/fit_diff_oscope.rds
    Untracked:  data/fit_diff_peco.rds
    Untracked:  data/fit_diff_recat.rds
    Untracked:  data/fit_diff_seurat.rds
    Untracked:  data/intensity.rds
    Untracked:  data/log2cpm.quant.rds
    Untracked:  data/macosko-2015.rds
    Untracked:  data/ourdata_cyclone_NA18511.rds
    Untracked:  data/ourdata_cyclone_NA18855.rds
    Untracked:  data/ourdata_cyclone_NA18870.rds
    Untracked:  data/ourdata_cyclone_NA19098.rds
    Untracked:  data/ourdata_cyclone_NA19101.rds
    Untracked:  data/ourdata_cyclone_NA19160.rds
    Untracked:  data/ourdata_oscope_366genes.rda
    Untracked:  data/ourdata_peco_NA18511_top005genes.rds
    Untracked:  data/ourdata_peco_NA18855_top005genes.rds
    Untracked:  data/ourdata_peco_NA18870_top005genes.rds
    Untracked:  data/ourdata_peco_NA19098_top005genes.rds
    Untracked:  data/ourdata_peco_NA19101_top005genes.rds
    Untracked:  data/ourdata_peco_NA19160_top005genes.rds
    Untracked:  data/ourdata_phase_cyclone.rds
    Untracked:  data/ourdata_phase_seurat.rds
    Untracked:  data/ourdata_recat.rda
    Untracked:  data/sce-filtered.rds

Unstaged changes:
    Modified:   analysis/eval_on_our_data.Rmd
    Modified:   analysis/index.Rmd
    Modified:   code/fig2.R
    Modified:   code/fig3.R
    Modified:   code/fig4.R
```

Note that any generated files, e.g. HTML, png, CSS, etc., are not included in this status report because it is ok for generated content to have uncommitted changes.

---

These are the previous versions of the R Markdown and HTML files. If you’ve configured a remote Git repository (see `?wflow_git_remote`), click on the hyperlinks in the table below to view them.

| File | Version | Author | Date | Message |
| --- | --- | --- | --- | --- |
| Rmd | fdebe2b | jhsiao999 | 2020-01-25 | code to compute prediction error in unthinned and thinned data |

---

## Introduction

## Set up

Load packages

```
library(SingleCellExperiment)
library(peco)
library(ggplot2)
library(dplyr)
```

Prepare training/testing data

```
sce <- readRDS("data/sce-final.rds")
sce <- sce[grep("ENSG", rownames(sce)),]
fdata <- data.frame(colData(sce))
fdata <- data.frame(rowData(sce))
counts <- data.frame(assay(sce, "counts"))

sce_normed <- data_transform_quantile(sce)
log2cpm_quant <- assay(sce_normed, "cpm_quantNormed")

inds <- c("NA18511", "NA18855", "NA18870", "NA19098", "NA19101", "NA19160")
theta <- pdata$theta

# function to make training/testing data
makedata_supervised <- function(sce, log2cpm_quant,
                                theta) {
  message("Create data/data_training_test folder \n")
  if (!file.exists("data/data_training_test")) { dir.create("data/data_training_test") }
  library(SingleCellExperiment)
  library(peco)
  pdata <- data.frame(colData(sce))
  fdata <- data.frame(rowData(sce))
  counts <- data.frame(assay(sce))
  counts <- counts[grep("ENSG", rownames(counts)), ]
  log2cpm <- t(log2(1+(10^6)*(t(counts)/pdata$molecules)))

  for (ind in unique(pdata$chip_id)) {
    ii_test <- c(1:nrow(pdata))[which(pdata$chip_id == ind)]
    ii_train <- c(1:nrow(pdata))[which(pdata$chip_id != ind)]

    pdata_test <- pdata[ii_test,]
    pdata_train <- pdata[ii_train,]

    log2cpm_quant_test <- log2cpm_quant[,ii_test]
    log2cpm_quant_train <- log2cpm_quant[,ii_train]
    theta <- pdata$theta
    names(theta) <- rownames(pdata)

    log2cpm_test <- log2cpm[,ii_test]
    log2cpm_train <- log2cpm[,ii_train]

    counts_test <- counts[,ii_test]
    counts_train <- counts[,ii_train]

    theta_test <- theta[ii_test]
    theta_train <- theta[ii_train]

    #sig.genes <- readRDS("output/npreg-trendfilter-quantile.Rmd/out.stats.ordered.sig.476.rds")
    data_training <- list(theta_train=theta_train,
                          log2cpm_quant_train=log2cpm_quant_train,
                          log2cpm_train=log2cpm_train,
                          counts_train=counts_train,
                          pdata_train=pdata_train,
                          fdata=fdata)

    data_test <- list(theta_test=theta_test,
                      log2cpm_quant_test=log2cpm_quant_test,
                      log2cpm_test=log2cpm_test,
                      counts_test = counts_test,
                      pdata_test=pdata_test,
                      fdata=fdata)

    saveRDS(data_training,
            file=file.path(paste0("data/data_training_test/ind_",ind,"_data_training.rds")))
    saveRDS(data_test,
            file=file.path(paste0("data/data_training_test/ind_",ind,"_data_test.rds")))
  }
}
makedata_supervised(sce, log2cpm_quant, theta)
```

Prepare thinned data. We used R package `seqgendiff` to thin the expression.

```
# function to make thinned data
makedata_thinned <- function(sce, thinlog2_rate) {

  library(SingleCellExperiment)
  library(peco)
  pdata <- data.frame(colData(sce))
  fdata <- data.frame(rowData(sce))
  counts <- assay(sce)[grep("ENSG", rownames(sce)), ]
  log2cpm <- t(log2(1+(10^6)*(t(counts)/pdata$molecules)))

  # ---- thinning!
  library(seqgendiff)
  nsamp <- ncol(counts)
  inputmat <- counts
  thinlog2 <- rexp(nsamp, rate = thinlog2_rate)
  outmat <- thin_lib(inputmat, thinlog2 = thinlog2)

  counts_thin <- outmat$mat
  dimnames(counts_thin) <- dimnames(counts)
  libsize <- colSums(counts_thin)

  keep_samples <- which(colMeans(counts_thin==0)>.01)
  keep_genes <- which(rowMeans(counts_thin == 0 ) < .5)
  counts_thin <- counts_thin[keep_genes, keep_samples]
  libsize <- libsize[keep_samples]

  log2cpm_thin <- t(log2(1+(10^6)*t(counts_thin)/libsize))
  dimnames(log2cpm_thin) <- dimnames(counts_thin)

  log2cpm_thin_quant <- do.call(rbind,
                                lapply(1:nrow(log2cpm_thin), function(i) {
                                  qqnorm(log2cpm_thin[i,], plot.it = F )$x }) )
  dimnames(log2cpm_thin_quant) <- dimnames(counts_thin)

  saveRDS(list(counts_thin=counts_thin,
               log2cpm_thin= log2cpm_thin,
               log2cpm_thin_quant=log2cpm_thin_quant),
          file=file.path(paste0("data/data_training_test/data_thinlog2_",
                                     sprintf("%03d", 100*thinlog2_rate),".rds")))

  pdata_thin <- pdata[keep_samples,]

  for (i in 1:length(unique(pdata$chip_id))) {
    ind <- unique(pdata_thin$chip_id)[i]
    ii_test <- c(1:nrow(pdata_thin))[which(pdata_thin$chip_id == ind)]
    counts_test <- counts[,ii_test]
    counts_thin_test <- counts_thin[,ii_test]
    log2cpm_thin_test <- log2cpm_thin[,ii_test]
    log2cpm_thin_quant_test <- log2cpm_thin_quant[,ii_test]

    data_thin_test <- list(counts_test=counts_test,
                           counts_thin_test=counts_thin_test,
                           log2cpm_thin_test=log2cpm_thin_test,
                           log2cpm_thin_quant_test=log2cpm_thin_quant_test)

    saveRDS(data_thin_test,
            file=file.path(paste0("data/data_training_test/ind_",ind,"_data_test_thinlog2_",
                                       sprintf("%03d", 100*thinlog2_rate),".rds")))
  }
}

makedata_thinned(sce, thinlog2_rate = .8)
makedata_thinned(sce, thinlog2_rate = .33)
```

## Properties of the thinned datasets

Library size

```
data_thinlog2_033 <- readRDS("data/data_training_test/data_thinlog2_033.rds")
data_thinlog2_080 <- readRDS("data/data_training_test/data_thinlog2_080.rds")


# thin library size by a factor of 4.24
mean(colSums(data_thinlog2_033$counts_thin))/mean(colSums(counts))
mean(colSums(counts)/(1/.23))
mean(colSums(data_thinlog2_033$counts_thin))
1/.23

# thin library size by a factor of 2.2
mean(colSums(data_thinlog2_080$counts_thin))/mean(colSums(counts))
mean(colSums(counts)/(1/.45))
mean(colSums(data_thinlog2_080$counts_thin))
1/.45
```

Number of genes detected

```
top5genes <- c("ENSG00000170312","ENSG00000175063",
           "ENSG00000131747", "ENSG00000198518", "ENSG00000197061")
inds <- unique(pdata$chip_id)

do.call(rbind, lapply(1:length(inds), function(i) {
  ind <- inds[i]
  samps <- rownames(pdata)[which(pdata$chip_id == ind)]
  exp_tmp <- data_thinlog2_080$counts_thin[,colnames(data_thinlog2_080$counts_thin) %in% samps]
  data.frame(ind = ind,
             g_detect = min(rowMeans(exp_tmp > 0)),
             g_detect_top50 = mean(rowMeans(exp_tmp[which(rownames(exp_tmp) %in% top5genes),] > 0)))
}))


do.call(rbind, lapply(1:length(inds), function(i) {
  ind <- inds[i]
  samps <- rownames(pdata)[which(pdata$chip_id == ind)]
  exp_tmp <- data_thinlog2_033$counts_thin[,colnames(data_thinlog2_033$counts_thin) %in% samps]
  data.frame(ind = ind,
             g_detect = min(rowMeans(exp_tmp > 0)))
}))

do.call(rbind, lapply(1:length(inds), function(i) {
  ind <- inds[i]
  samps <- rownames(pdata)[which(pdata$chip_id == ind)]
  exp_tmp <- counts[,colnames(counts) %in% samps]
  data.frame(ind = ind,
             g_detect = min(rowMeans(exp_tmp > 0)))
}))
```

Plot out properties of thinned data

```
# checking...
thing_080 <- readRDS("data/data_training_test/data_thinlog2_080.rds")
thing_033 <- readRDS("data/data_training_test/data_thinlog2_033.rds")

subsam <- do.call(rbind, list(data.frame(libsize=colSums(counts), thinlog2="1"),
                              data.frame(libsize=colSums(thing_080$counts_thin), thinlog2="080"),
                              data.frame(libsize=colSums(thing_033$counts_thin), thinlog2="033")))
subsam$thinlog2 <- factor(subsam$thinlog2,
                          levels=c("033", "080", "1"),
                          labels=c("4.4", "2.2", "1 (none)"))
ggplot(subsam, aes(x=thinlog2, y=libsize, group=thinlog2, fill=thinlog2)) +
  geom_violin() +
  geom_boxplot(width=.2, col="black") +
  labs(fill="Thinning factor") +
  ylab("Sample molecule count") +
  xlab("Thinning factor")
subsam %>% group_by(thinlog2) %>% summarize(mn = mean(libsize), sd = sd(libsize))
```

## Compute prediction error on unthinned data

```
fits_all <- readRDS("data/fit.quant.rds")
genes_all <- names(fits_all)[order(sapply(fits_all,"[[",3), decreasing=T)]

res_unthinned <- do.call(rbind, lapply(seq_along(unique(pdata$chip_id)), function(ind) {
    ind <- unique(pdata$chip_id)[i]
    res_thin_each <- do.call(rbind, lapply(2:50, function(ngenes) {
        data_test <- readRDS(paste0("data/data_training_test/ind_",ind,"_data_test.rds"))
        data_train <- readRDS(paste0("data/data_training_test/ind_",ind,"_data_training.rds"))

        which_genes <- genes_all[1:ngenes]
        fit_train <- cycle_npreg_insample(
          Y = with(data_train, 
                   log2cpm_quant_train[which(rownames(log2cpm_quant_train) %in% which_genes), ]),
          theta = with(data_train, theta_train))
        fit_test <- cycle_npreg_outsample(
            Y_test=with(data_test, 
                        log2cpm_quant_test[which(rownames(log2cpm_quant_test) %in% which_genes), ]),
            sigma_est=with(fit_train, sigma_est),
            funs_est=with(fit_train, funs_est))
    
        diff_time <- circ_dist(data_test$theta_test,
                               rotation(data_test$theta_test, fit_test$cell_times_est))
        out <- data.frame(phase_pred_rot=rotation(data_test$theta_test, fit_test$cell_times_est),
                   phase_ref=data_test$theta_test,
                   diff_time=diff_time,
                   ind = ind,
                   ngenes = ngenes)
        return(out)
    }) )
}) )


res_unthinned %>% group_by(ind, ngenes) %>%
  #  filter(ngenes <= 20) %>%
  summarise(diff_mean = mean(diff_time/2/pi),
            diff_se = sd(diff_time/2/pi)/sqrt(length(diff_time/2/pi))) %>%
  ggplot(., aes(x=factor(ngenes), y=diff_mean, group = ind)) +
  #  geom_vline(xintercept=seq(5, 50,5)-1, col="gray90", lty=1) +
  geom_hline(yintercept=seq(.1, .2, .01), col="gray90", lty=1) +
  geom_line(aes(col=ind), lwd=.7) + # ggtitle("thinlog2 = .80") +
  scale_fill_brewer(palette="Dark2") +
  geom_errorbar(aes(ymin=diff_mean-diff_se,
                    ymax=diff_mean+diff_se, col=ind), width=.2, alpha=.5) +
  stat_summary(fun.y=mean,geom="line",lwd=.5, group=1) +
  ylim(0,.3) + geom_hline(yintercept=.25, col="red") +
  labs(color="Test data (Cell line)") +
  xlab("Number of cyclic genes used in peco prediction") +
  ylab("Prediction error (% circle)") +
  scale_x_discrete(breaks=c(2:50),
                   labels=c(rep("",3),5, rep("",4), 10, rep("",4), 15,
                            rep("",4), 20, rep("",4), 25,
                            rep("",4), 30, rep("",4), 35,
                            rep("",4), 40, rep("",4), 45, rep("",4), 50)) +
  ggtitle("Performance in unthinned data")
```

## Compute prediction error on thinned data

Use peco predictors trained on unthinned data of samples from 5 individual cell lines to predict cell cycle phase in thinned data of samples from an individual not seen in training.

```
fits_all <- readRDS("data/fit.quant.rds")
genes_all <- names(fits_all)[order(sapply(fits_all,"[[",3), decreasing=T)]

res_thin_080 <- do.call(rbind, lapply(seq_along(unique(pdata$chip_id)), function(ind) {
    ind <- unique(pdata$chip_id)[i]
    res_thin_each <- do.call(rbind, lapply(2:50, function(ngenes) {
        data_test <- readRDS(paste0("data/data_training_test/ind_",ind,"_data_test.rds"))
        data_train <- readRDS(paste0("data/data_training_test/ind_",ind,"_data_training.rds"))

        which_genes <- genes_all[1:ngenes]
        fit_train <- cycle_npreg_insample(
          Y = with(data_train, 
                   log2cpm_quant_train[which(rownames(log2cpm_quant_train) %in% which_genes), ]),
          theta = with(data_train, theta_train))
        
        data_thin_test_080 <- readRDS(paste0("data/data_training_test/ind_", 
                                           ind, "_data_test_thinlog2_080.rds"))
        fit_test_080 <- cycle_npreg_outsample(
            Y_test=with(data_thin_test_080, 
                        log2cpm_thin_quant_test[which(rownames(log2cpm_thin_quant_test) %in% which_genes), ]),
            sigma_est=with(fit_train, sigma_est),
            funs_est=with(fit_train, funs_est))
    
        diff_time <- circ_dist(data_test$theta_test,
                               rotation(data_test$theta_test, fit_test_080$cell_times_est))
        out <- data.frame(phase_pred_rot=rotation(data_test$theta_test, fit_test$cell_times_est),
                   phase_ref=data_test$theta_test,
                   diff_time=diff_time,
                   ind = ind,
                   ngenes = ngenes)
        return(out)
    }) )
}) )

res_thin_033 %>% group_by(ind, ngenes) %>%
  #  filter(ngenes <= 20) %>%
  summarise(diff_mean = mean(diff_time/2/pi),
            diff_se = sd(diff_time/2/pi)/sqrt(length(diff_time/2/pi))) %>%
  ggplot(., aes(x=factor(ngenes), y=diff_mean, group = ind)) +
  #  geom_vline(xintercept=seq(5, 50,5)-1, col="gray90", lty=1) +
  geom_hline(yintercept=seq(.1, .2, .01), col="gray90", lty=1) +
  geom_line(aes(col=ind), lwd=.7) + # ggtitle("thinlog2 = .80") +
  scale_fill_brewer(palette="Dark2") +
  geom_errorbar(aes(ymin=diff_mean-diff_se,
                    ymax=diff_mean+diff_se, col=ind), width=.2, alpha=.5) +
  stat_summary(fun.y=mean,geom="line",lwd=.5, group=1) +
  ylim(0,.3) + geom_hline(yintercept=.25, col="red") +
  labs(color="Test data (Cell line)") +
  xlab("Number of cyclic genes used in peco prediction") +
  ylab("Prediction error (% circle)") +
  scale_x_discrete(breaks=c(2:50),
                   labels=c(rep("",3),5, rep("",4), 10, rep("",4), 15,
                            rep("",4), 20, rep("",4), 25,
                            rep("",4), 30, rep("",4), 35,
                            rep("",4), 40, rep("",4), 45, rep("",4), 50)) +
  ggtitle("Performance in data thinned by a factor of 2.2")


res_thin_033 <- do.call(rbind, lapply(seq_along(unique(pdata$chip_id)), function(ind) {
    ind <- unique(pdata$chip_id)[i]
    res_thin_each <- do.call(rbind, lapply(2:50, function(ngenes) {
        data_test <- readRDS(paste0("data/data_training_test/ind_",ind,"_data_test.rds"))
        data_train <- readRDS(paste0("data/data_training_test/ind_",ind,"_data_training.rds"))

        which_genes <- genes_all[1:ngenes]
        fit_train <- cycle_npreg_insample(
          Y = with(data_train, 
                   log2cpm_quant_train[which(rownames(log2cpm_quant_train) %in% which_genes), ]),
          theta = with(data_train, theta_train))
        
        data_thin_test_033 <- readRDS(paste0("data/data_training_test/ind_", 
                                           ind, "_data_test_thinlog2_033.rds"))
        fit_test_033 <- cycle_npreg_outsample(
            Y_test=with(data_thin_test_033, 
                        log2cpm_thin_quant_test[which(rownames(log2cpm_thin_quant_test) %in% which_genes), ]),
            sigma_est=with(fit_train, sigma_est),
            funs_est=with(fit_train, funs_est))
    
        diff_time <- circ_dist(data_test$theta_test,
                               rotation(data_test$theta_test, fit_test_033$cell_times_est))
        out <- data.frame(phase_pred_rot=rotation(data_test$theta_test, fit_test$cell_times_est),
                   phase_ref=data_test$theta_test,
                   diff_time=diff_time,
                   ind = ind,
                   ngenes = ngenes)
        return(out)
    }) )
}) )


res_thin_033 %>% group_by(ind, ngenes) %>%
  #  filter(ngenes <= 20) %>%
  summarise(diff_mean = mean(diff_time/2/pi),
            diff_se = sd(diff_time/2/pi)/sqrt(length(diff_time/2/pi))) %>%
  ggplot(., aes(x=factor(ngenes), y=diff_mean, group = ind)) +
  #  geom_vline(xintercept=seq(5, 50,5)-1, col="gray90", lty=1) +
  geom_hline(yintercept=seq(.1, .2, .01), col="gray90", lty=1) +
  geom_line(aes(col=ind), lwd=.7) + # ggtitle("thinlog2 = .80") +
  scale_fill_brewer(palette="Dark2") +
  geom_errorbar(aes(ymin=diff_mean-diff_se,
                    ymax=diff_mean+diff_se, col=ind), width=.2, alpha=.5) +
  stat_summary(fun.y=mean,geom="line",lwd=.5, group=1) +
  ylim(0,.3) + geom_hline(yintercept=.25, col="red") +
  labs(color="Test data (Cell line)") +
  xlab("Number of cyclic genes used in peco prediction") +
  ylab("Prediction error (% circle)") +
  scale_x_discrete(breaks=c(2:50),
                   labels=c(rep("",3),5, rep("",4), 10, rep("",4), 15,
                            rep("",4), 20, rep("",4), 25,
                            rep("",4), 30, rep("",4), 35,
                            rep("",4), 40, rep("",4), 45, rep("",4), 50)) +
  ggtitle("Performance in data thinned by a factor of 4.3")
```

## Session information

```
sessionInfo()
```

```
R version 3.5.1 (2018-07-02)
Platform: x86_64-pc-linux-gnu (64-bit)
Running under: Scientific Linux 7.4 (Nitrogen)

Matrix products: default
BLAS/LAPACK: /software/openblas-0.2.19-el7-x86_64/lib/libopenblas_haswellp-r0.2.19.so

locale:
 [1] LC_CTYPE=en_US.UTF-8       LC_NUMERIC=C              
 [3] LC_TIME=en_US.UTF-8        LC_COLLATE=en_US.UTF-8    
 [5] LC_MONETARY=en_US.UTF-8    LC_MESSAGES=en_US.UTF-8   
 [7] LC_PAPER=en_US.UTF-8       LC_NAME=C                 
 [9] LC_ADDRESS=C               LC_TELEPHONE=C            
[11] LC_MEASUREMENT=en_US.UTF-8 LC_IDENTIFICATION=C       

attached base packages:
[1] parallel  stats4    stats     graphics  grDevices utils     datasets 
[8] methods   base     

other attached packages:
 [1] dplyr_0.8.0.1               ggplot2_3.2.1              
 [3] peco_0.99.6                 SingleCellExperiment_1.4.1 
 [5] SummarizedExperiment_1.12.0 DelayedArray_0.8.0         
 [7] BiocParallel_1.16.0         matrixStats_0.55.0         
 [9] Biobase_2.42.0              GenomicRanges_1.34.0       
[11] GenomeInfoDb_1.18.1         IRanges_2.16.0             
[13] S4Vectors_0.20.1            BiocGenerics_0.28.0        

loaded via a namespace (and not attached):
 [1] viridis_0.5.1            genlasso_1.4            
 [3] viridisLite_0.3.0        foreach_1.4.4           
 [5] DelayedMatrixStats_1.4.0 assertthat_0.2.1        
 [7] vipor_0.4.5              GenomeInfoDbData_1.2.0  
 [9] yaml_2.2.0               pillar_1.3.1            
[11] backports_1.1.2          lattice_0.20-38         
[13] glue_1.3.0               digest_0.6.20           
[15] promises_1.0.1           XVector_0.22.0          
[17] colorspace_1.3-2         plyr_1.8.4              
[19] htmltools_0.3.6          httpuv_1.4.5            
[21] Matrix_1.2-17            pkgconfig_2.0.3         
[23] zlibbioc_1.28.0          purrr_0.3.2             
[25] mvtnorm_1.0-11           scales_1.0.0            
[27] HDF5Array_1.10.1         whisker_0.3-2           
[29] later_0.7.5              pracma_2.2.9            
[31] git2r_0.26.1             tibble_2.1.1            
[33] conicfit_1.0.4           withr_2.1.2             
[35] lazyeval_0.2.1           magrittr_1.5            
[37] crayon_1.3.4             evaluate_0.12           
[39] fs_1.3.1                 doParallel_1.0.14       
[41] MASS_7.3-51.1            beeswarm_0.2.3          
[43] geigen_2.3               tools_3.5.1             
[45] scater_1.10.1            stringr_1.3.1           
[47] Rhdf5lib_1.4.3           munsell_0.5.0           
[49] compiler_3.5.1           rlang_0.4.0             
[51] rhdf5_2.26.2             grid_3.5.1              
[53] RCurl_1.95-4.11          iterators_1.0.12        
[55] circular_0.4-93          igraph_1.2.2            
[57] bitops_1.0-6             rmarkdown_1.10          
[59] boot_1.3-20              gtable_0.2.0            
[61] codetools_0.2-15         reshape2_1.4.3          
[63] R6_2.4.0                 gridExtra_2.3           
[65] knitr_1.20               workflowr_1.6.0         
[67] rprojroot_1.3-2          stringi_1.2.4           
[69] ggbeeswarm_0.6.0         Rcpp_1.0.3              
[71] tidyselect_0.2.5
```

  

Session information

```
sessionInfo()
```

```
R version 3.5.1 (2018-07-02)
Platform: x86_64-pc-linux-gnu (64-bit)
Running under: Scientific Linux 7.4 (Nitrogen)

Matrix products: default
BLAS/LAPACK: /software/openblas-0.2.19-el7-x86_64/lib/libopenblas_haswellp-r0.2.19.so

locale:
 [1] LC_CTYPE=en_US.UTF-8       LC_NUMERIC=C              
 [3] LC_TIME=en_US.UTF-8        LC_COLLATE=en_US.UTF-8    
 [5] LC_MONETARY=en_US.UTF-8    LC_MESSAGES=en_US.UTF-8   
 [7] LC_PAPER=en_US.UTF-8       LC_NAME=C                 
 [9] LC_ADDRESS=C               LC_TELEPHONE=C            
[11] LC_MEASUREMENT=en_US.UTF-8 LC_IDENTIFICATION=C       

attached base packages:
[1] parallel  stats4    stats     graphics  grDevices utils     datasets 
[8] methods   base     

other attached packages:
 [1] dplyr_0.8.0.1               ggplot2_3.2.1              
 [3] peco_0.99.6                 SingleCellExperiment_1.4.1 
 [5] SummarizedExperiment_1.12.0 DelayedArray_0.8.0         
 [7] BiocParallel_1.16.0         matrixStats_0.55.0         
 [9] Biobase_2.42.0              GenomicRanges_1.34.0       
[11] GenomeInfoDb_1.18.1         IRanges_2.16.0             
[13] S4Vectors_0.20.1            BiocGenerics_0.28.0        

loaded via a namespace (and not attached):
 [1] viridis_0.5.1            genlasso_1.4            
 [3] viridisLite_0.3.0        foreach_1.4.4           
 [5] DelayedMatrixStats_1.4.0 assertthat_0.2.1        
 [7] vipor_0.4.5              GenomeInfoDbData_1.2.0  
 [9] yaml_2.2.0               pillar_1.3.1            
[11] backports_1.1.2          lattice_0.20-38         
[13] glue_1.3.0               digest_0.6.20           
[15] promises_1.0.1           XVector_0.22.0          
[17] colorspace_1.3-2         plyr_1.8.4              
[19] htmltools_0.3.6          httpuv_1.4.5            
[21] Matrix_1.2-17            pkgconfig_2.0.3         
[23] zlibbioc_1.28.0          purrr_0.3.2             
[25] mvtnorm_1.0-11           scales_1.0.0            
[27] HDF5Array_1.10.1         whisker_0.3-2           
[29] later_0.7.5              pracma_2.2.9            
[31] git2r_0.26.1             tibble_2.1.1            
[33] conicfit_1.0.4           withr_2.1.2             
[35] lazyeval_0.2.1           magrittr_1.5            
[37] crayon_1.3.4             evaluate_0.12           
[39] fs_1.3.1                 doParallel_1.0.14       
[41] MASS_7.3-51.1            beeswarm_0.2.3          
[43] geigen_2.3               tools_3.5.1             
[45] scater_1.10.1            stringr_1.3.1           
[47] Rhdf5lib_1.4.3           munsell_0.5.0           
[49] compiler_3.5.1           rlang_0.4.0             
[51] rhdf5_2.26.2             grid_3.5.1              
[53] RCurl_1.95-4.11          iterators_1.0.12        
[55] circular_0.4-93          igraph_1.2.2            
[57] bitops_1.0-6             rmarkdown_1.10          
[59] boot_1.3-20              gtable_0.2.0            
[61] codetools_0.2-15         reshape2_1.4.3          
[63] R6_2.4.0                 gridExtra_2.3           
[65] knitr_1.20               workflowr_1.6.0         
[67] rprojroot_1.3-2          stringi_1.2.4           
[69] ggbeeswarm_0.6.0         Rcpp_1.0.3              
[71] tidyselect_0.2.5
```
