# Supplementary material for: Characterizing and inferring quantitative cell cycle phase in single-cell RNA-seq data analysis
Source: Genome Res. 2020 Apr;30(4):611–21. doi: 10.1101/gr.247759.118 (PMC7197478; doi:10.1101/gr.247759.118)
Supplement: Supplemental Material [file supp_gr.247759.118_Supplemental_peco-paper-master-source-code.tar.gz › peco-paper-master/docs/reads-v-molecules.html]

Read to molecule conversion


peco-paper

- Home
- About
- License

- Source code


# Read to molecule conversion

#### *John Blischak*

#### *2017-11-29*

workflowr

- Summary
- Checks
- Past versions

**Last updated:** 2020-01-23

**Checks:**  7  0

**Knit directory:** `peco-paper/`

This reproducible R Markdown analysis was created with workflowr (version 1.6.0). The *Checks* tab describes the reproducibility checks that were applied when the results were created. The *Past versions* tab lists the development history.

---

**R Markdown file:** up-to-date

Great! Since the R Markdown file has been committed to the Git repository, you know the exact version of the code that produced these results.

**Environment:** empty

Great job! The global environment was empty. Objects defined in the global environment can affect the analysis in your R Markdown file in unknown ways. For reproduciblity it’s best to always run the code in an empty environment.

**Seed:** `set.seed(20190814)`

The command `set.seed(20190814)` was run prior to running the code in the R Markdown file. Setting a seed ensures that any results that rely on randomness, e.g. subsampling or permutations, are reproducible.

**Session information:** recorded

Great job! Recording the operating system, R version, and package versions is critical for reproducibility.

**Cache:** none

Nice! There were no cached chunks for this analysis, so you can be confident that you successfully produced the results during this run.

**File paths:** relative

Great job! Using relative paths to the files within your workflowr project makes it easier to run your code on other machines.

**Repository version:** fdc569c

Great! You are using Git for version control. Tracking code development and connecting the code version to the results is critical for reproducibility. The version displayed above was the version of the Git repository at the time these results were generated.   
  
 Note that you need to be careful to ensure that all relevant files for the analysis have been committed to Git prior to generating the results (you can use `wflow_publish` or `wflow_git_commit`). workflowr only checks the R Markdown file, but you know if there are other scripts or data files that it depends on. Below is the status of the Git repository when the results were generated:

```
Ignored files:
    Ignored:    .Rhistory
    Ignored:    .Rproj.user/

Untracked files:
    Untracked:  analysis/npreg_trendfilter_quantile.Rmd
    Untracked:  code/fig2_rev.R
    Untracked:  data/fit.quant.rds
    Untracked:  data/log2cpm.quant.rds

Unstaged changes:
    Modified:   analysis/access_data.Rmd
    Modified:   analysis/index.Rmd
    Modified:   code/fig2.R
```

Note that any generated files, e.g. HTML, png, CSS, etc., are not included in this status report because it is ok for generated content to have uncommitted changes.

---

These are the previous versions of the R Markdown and HTML files. If you’ve configured a remote Git repository (see `?wflow_git_remote`), click on the hyperlinks in the table below to view them.

| File | Version | Author | Date | Message |
| --- | --- | --- | --- | --- |
| Rmd | fdc569c | jhsiao999 | 2020-01-23 | move reads-v-molecules.Rmd and change eset to sce |

---

## Setup

```
library("cowplot")
library("dplyr")
library("DT")
library("ggplot2")
library("reshape2")
library("SingleCellExperiment")
theme_set(theme_cowplot())
```

```
sce_raw = readRDS("data/sce-raw.rds")
anno = data.frame(colData(sce_raw))
anno$experiment = factor(anno$experiment)
```

## Reads versus molecules

```
conv_hs_c1 <- ggplot(anno, aes(x = reads_hs, y = mol_hs,
                                     color = experiment)) +
  geom_point(alpha = 1/2) +
  labs(x = "Total read count",
       y = "Total molecule count",
       title = "Endogenous genes by C1 chip") +
  theme(legend.position = "none")

conv_hs_ind <- ggplot(anno, aes(x = reads_hs, y = mol_hs,
                                      color = chip_id)) +
  geom_point(alpha = 1/2) +
  scale_color_brewer(palette = "Dark2") +
  labs(x = "Total read count",
       y = "Total molecule count",
       title = "Endogenous genes by individual") +
  theme(legend.position = "none")

conv_ercc_c1 <- ggplot(anno, aes(x = reads_ercc, y = mol_ercc,
                                        color = experiment)) +
  geom_point(alpha = 1/2) +
  labs(x = "Total read count",
       y = "Total molecule count",
       title = "ERCC genes by C1 chip") +
  theme(legend.position = "none")

conv_ercc_ind <- ggplot(anno, aes(x = reads_ercc, y = mol_ercc,
                                         color = chip_id)) +
  geom_point(alpha = 1/2) +
  scale_color_brewer(palette = "Dark2") +
  labs(x = "Total read count",
       y = "Total molecule count",
       title = "ERCC genes by individual") +
  theme(legend.position = "none")

plot_grid(conv_hs_c1, conv_hs_ind, conv_ercc_c1, conv_ercc_ind,
        labels = letters[1:4])
```

## Read to molecule conversion

```
anno$conv_hs <- anno$mol_hs / anno$reads_hs
anno$conv_ercc <- anno$mol_ercc / anno$reads_ercc

r2_hs_c1 <- summary(lm(conv_hs ~ experiment, data = anno))$r.squared
box_hs_c1 <- ggplot(anno, aes(x = experiment, y = conv_hs,
                                     fill = experiment)) +
  geom_boxplot() +
  labs(x = "C1 chip", y = "Conversion efficiency",
       title = sprintf("Endogenous genes R-squared: %.2f", r2_hs_c1)) +
  theme(legend.position = "none",
        axis.text.x = element_text(angle = 45, hjust = 1, vjust = 1))

r2_hs_ind <- summary(lm(conv_hs ~ chip_id, data = anno))$r.squared
box_hs_ind <- ggplot(anno, aes(x = chip_id, y = conv_hs,
                                      fill = chip_id)) +
  geom_boxplot() +
  scale_fill_brewer(palette = "Dark2") +
  labs(x = "Individual", y = "Conversion efficiency",
       title = sprintf("Endogenous genes R-squared: %.2f", r2_hs_ind)) +
  theme(legend.position = "none",
        axis.text.x = element_text(angle = 45, hjust = 1, vjust = 1))

r2_ercc_c1 <- summary(lm(conv_ercc ~ experiment, data = anno))$r.squared
box_ercc_c1 <- ggplot(anno, aes(x = experiment, y = conv_ercc,
                                       fill = experiment)) +
  geom_boxplot() +
  labs(x = "C1 chip", y = "Conversion efficiency",
       title = sprintf("ERCC genes R-squared: %.2f", r2_ercc_c1)) +
  theme(legend.position = "none",
        axis.text.x = element_text(angle = 45, hjust = 1, vjust = 1))

r2_ercc_ind <- summary(lm(conv_ercc ~ chip_id, data = anno))$r.squared
box_ercc_ind <- ggplot(anno, aes(x = chip_id, y = conv_ercc,
                                        fill = chip_id)) +
  geom_boxplot() +
  scale_fill_brewer(palette = "Dark2") +
  labs(x = "Individual", y = "Conversion efficiency",
       title = sprintf("ERCC genes R-squared: %.2f", r2_ercc_ind)) +
  theme(legend.position = "none",
        axis.text.x = element_text(angle = 45, hjust = 1, vjust = 1))

plot_grid(box_hs_c1, box_hs_ind, box_ercc_c1, box_ercc_ind,
          labels = letters[1:4])
```

## Total ERCC versus total endogenous molecules

Recreating Tung et al., 2017 Figure 3b:

Tung et al., 2017 Figure 3b

```
gene_v_ercc_c1 <- ggplot(anno, aes(x = mol_hs, y = mol_ercc,
                        color = experiment)) +
  geom_point(alpha = 1/2) +
  labs(x = "Total gene molecule-counts per sample",
       y = "Total ERCC molecule-counts per sample",
       title = "C1 chip") +
  theme(legend.position = "none")

gene_v_ercc_ind <- ggplot(anno, aes(x = mol_hs, y = mol_ercc,
                                           color = chip_id)) +
  geom_point(alpha = 1/2) +
  scale_color_brewer(palette = "Dark2") +
  labs(x = "Total gene molecule-counts per sample",
       y = "Total ERCC molecule-counts per sample",
       title = "Individual") +
  theme(legend.position = "none")

plot_grid(gene_v_ercc_c1, gene_v_ercc_ind, labels = letters[1:2])
```

  

Session information

```
sessionInfo()
```

```
R version 3.5.1 (2018-07-02)
Platform: x86_64-pc-linux-gnu (64-bit)
Running under: Scientific Linux 7.4 (Nitrogen)

Matrix products: default
BLAS/LAPACK: /software/openblas-0.2.19-el7-x86_64/lib/libopenblas_haswellp-r0.2.19.so

locale:
 [1] LC_CTYPE=en_US.UTF-8       LC_NUMERIC=C              
 [3] LC_TIME=en_US.UTF-8        LC_COLLATE=en_US.UTF-8    
 [5] LC_MONETARY=en_US.UTF-8    LC_MESSAGES=en_US.UTF-8   
 [7] LC_PAPER=en_US.UTF-8       LC_NAME=C                 
 [9] LC_ADDRESS=C               LC_TELEPHONE=C            
[11] LC_MEASUREMENT=en_US.UTF-8 LC_IDENTIFICATION=C       

attached base packages:
[1] parallel  stats4    stats     graphics  grDevices utils     datasets 
[8] methods   base     

other attached packages:
 [1] SingleCellExperiment_1.4.1  SummarizedExperiment_1.12.0
 [3] DelayedArray_0.8.0          BiocParallel_1.16.0        
 [5] matrixStats_0.55.0          Biobase_2.42.0             
 [7] GenomicRanges_1.34.0        GenomeInfoDb_1.18.1        
 [9] IRanges_2.16.0              S4Vectors_0.20.1           
[11] BiocGenerics_0.28.0         reshape2_1.4.3             
[13] DT_0.5                      dplyr_0.8.0.1              
[15] cowplot_0.9.4               ggplot2_3.2.1              

loaded via a namespace (and not attached):
 [1] tidyselect_0.2.5       purrr_0.3.2            lattice_0.20-38       
 [4] colorspace_1.3-2       htmltools_0.3.6        yaml_2.2.0            
 [7] rlang_0.4.0            later_0.7.5            pillar_1.3.1          
[10] glue_1.3.0             withr_2.1.2            RColorBrewer_1.1-2    
[13] GenomeInfoDbData_1.2.0 plyr_1.8.4             stringr_1.3.1         
[16] zlibbioc_1.28.0        munsell_0.5.0          gtable_0.2.0          
[19] workflowr_1.6.0        htmlwidgets_1.3        evaluate_0.12         
[22] labeling_0.3           knitr_1.20             httpuv_1.4.5          
[25] Rcpp_1.0.3             promises_1.0.1         scales_1.0.0          
[28] backports_1.1.2        XVector_0.22.0         fs_1.3.1              
[31] digest_0.6.20          stringi_1.2.4          grid_3.5.1            
[34] rprojroot_1.3-2        tools_3.5.1            bitops_1.0-6          
[37] magrittr_1.5           lazyeval_0.2.1         RCurl_1.95-4.11       
[40] tibble_2.1.1           crayon_1.3.4           whisker_0.3-2         
[43] pkgconfig_2.0.3        Matrix_1.2-17          assertthat_0.2.1      
[46] rmarkdown_1.10         R6_2.4.0               git2r_0.26.1          
[49] compiler_3.5.1
```
