# Supplementary figures and images for: Characterizing and inferring quantitative cell cycle phase in single-cell RNA-seq data analysis
Source: Genome Res. 2020 Apr;30(4):611–21. doi: 10.1101/gr.247759.118 (PMC7197478; doi:10.1101/gr.247759.118)

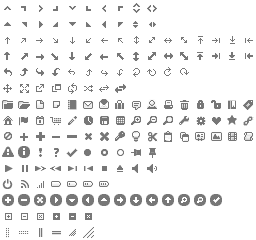

Supplement: Supplemental Material [file supp_gr.247759.118_Supplemental_peco-paper-master-source-code.tar.gz › peco-paper-master/docs/site_libs/jqueryui-1.11.4/images/ui-icons_777777_256x240.png]

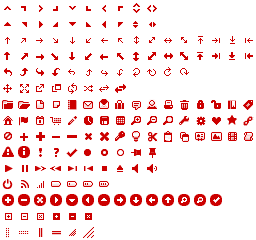

Supplement: Supplemental Material [file supp_gr.247759.118_Supplemental_peco-paper-master-source-code.tar.gz › peco-paper-master/docs/site_libs/jqueryui-1.11.4/images/ui-icons_cc0000_256x240.png]

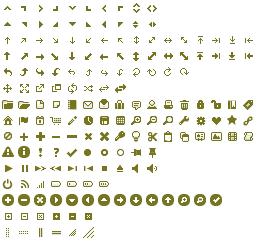

Supplement: Supplemental Material [file supp_gr.247759.118_Supplemental_peco-paper-master-source-code.tar.gz › peco-paper-master/docs/site_libs/jqueryui-1.11.4/images/ui-icons_777620_256x240.png]

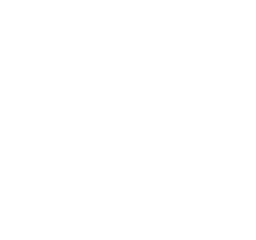

Supplement: Supplemental Material [file supp_gr.247759.118_Supplemental_peco-paper-master-source-code.tar.gz › peco-paper-master/docs/site_libs/jqueryui-1.11.4/images/ui-icons_ffffff_256x240.png]

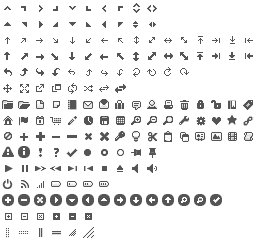

Supplement: Supplemental Material [file supp_gr.247759.118_Supplemental_peco-paper-master-source-code.tar.gz › peco-paper-master/docs/site_libs/jqueryui-1.11.4/images/ui-icons_555555_256x240.png]

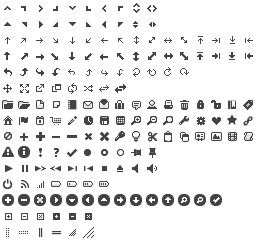

Supplement: Supplemental Material [file supp_gr.247759.118_Supplemental_peco-paper-master-source-code.tar.gz › peco-paper-master/docs/site_libs/jqueryui-1.11.4/images/ui-icons_444444_256x240.png]

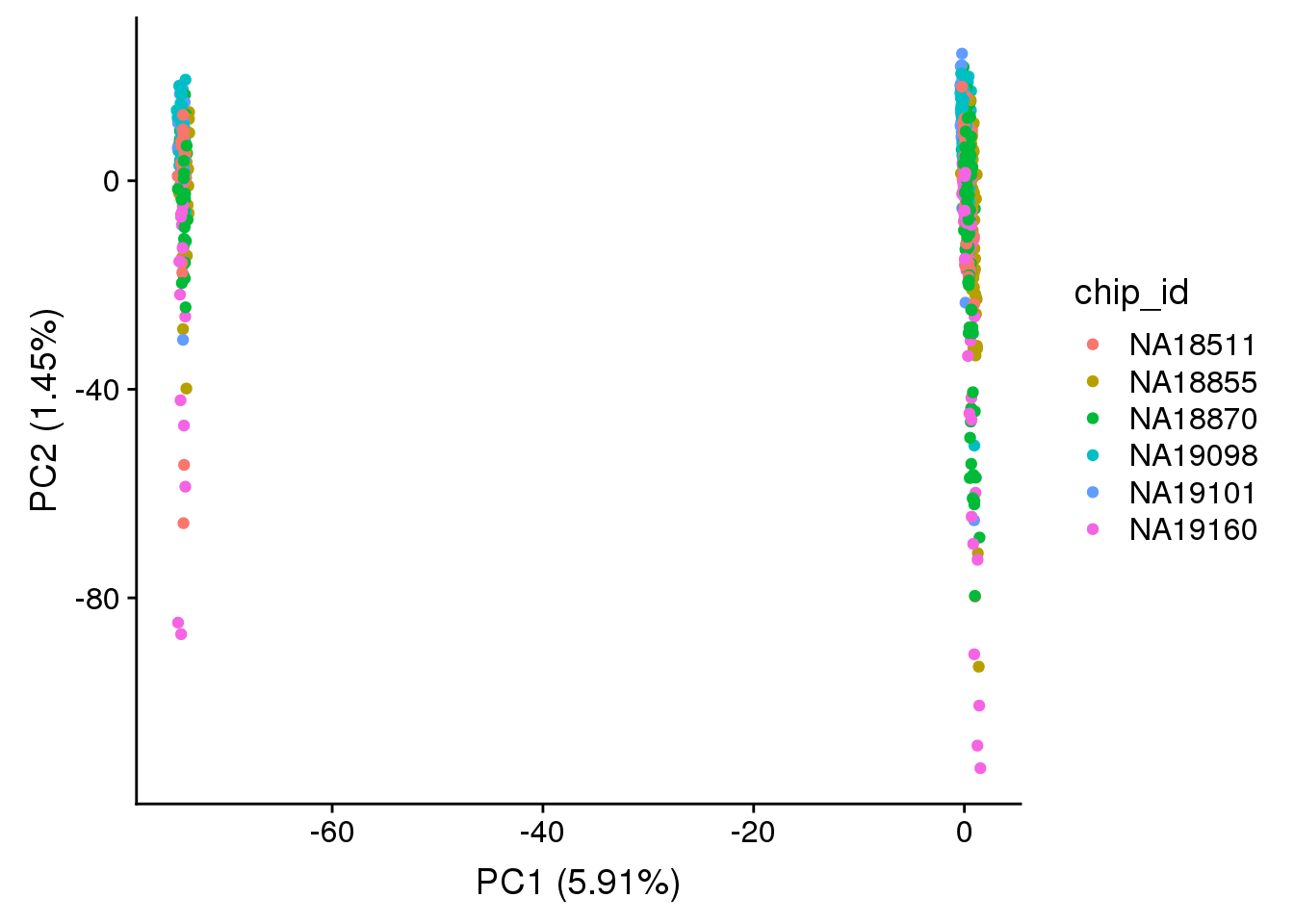

Supplement: Supplemental Material [file supp_gr.247759.118_Supplemental_peco-paper-master-source-code.tar.gz › peco-paper-master/docs/figure/pca_tf.Rmd/after-filter-1.png]

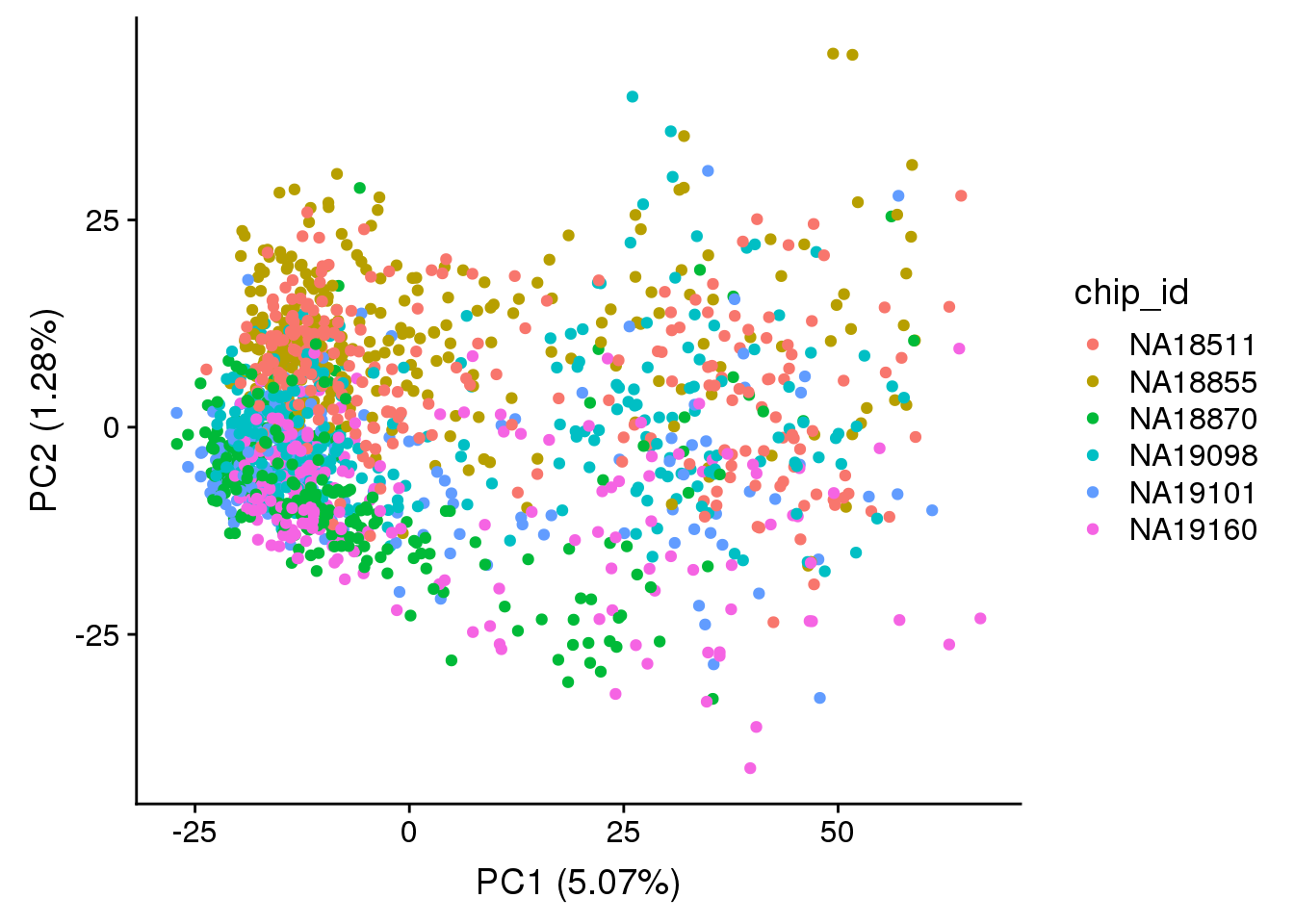

Supplement: Supplemental Material [file supp_gr.247759.118_Supplemental_peco-paper-master-source-code.tar.gz › peco-paper-master/docs/figure/pca_tf.Rmd/before-filter-2.png]

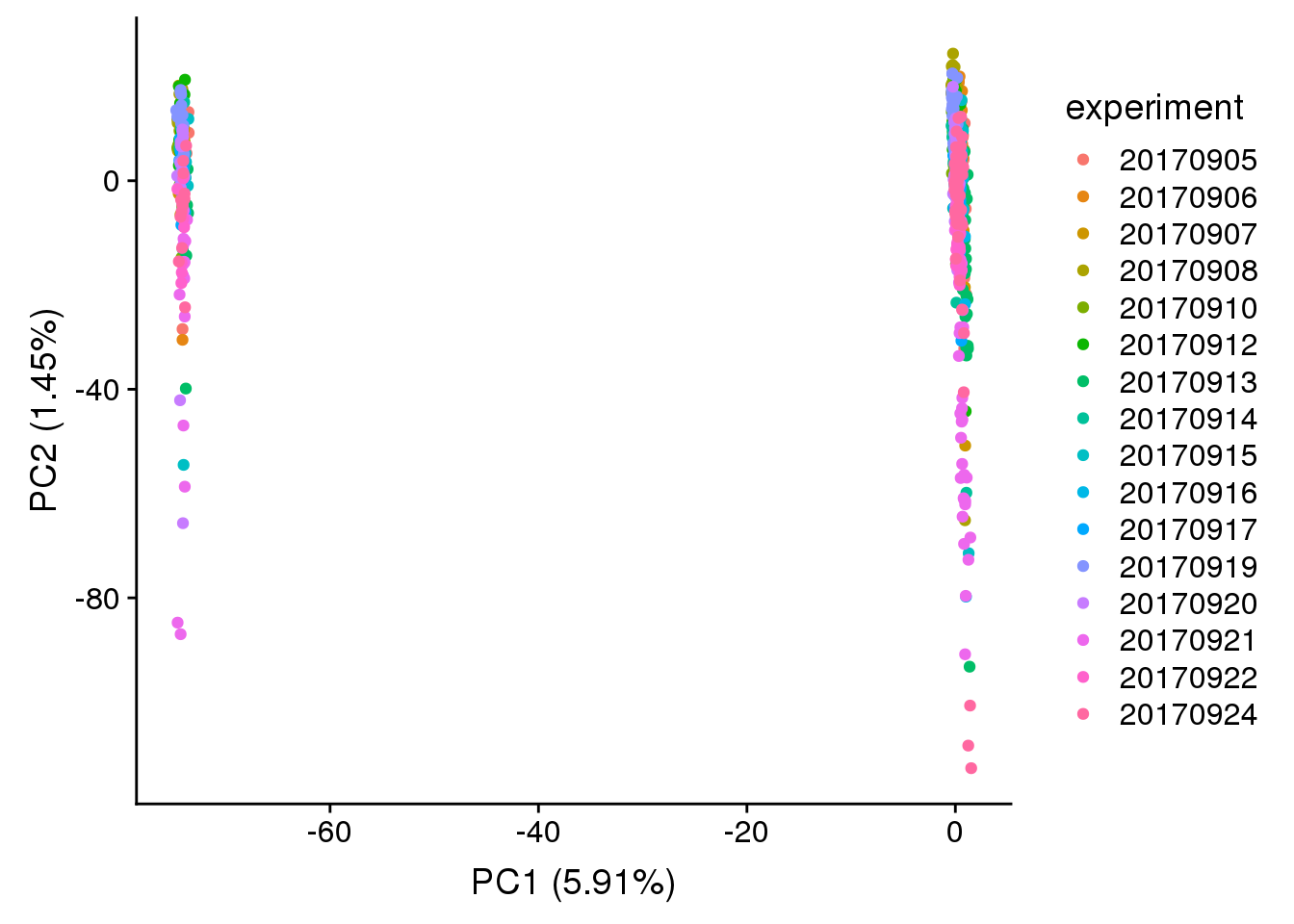

Supplement: Supplemental Material [file supp_gr.247759.118_Supplemental_peco-paper-master-source-code.tar.gz › peco-paper-master/docs/figure/pca_tf.Rmd/after-filter-2.png]

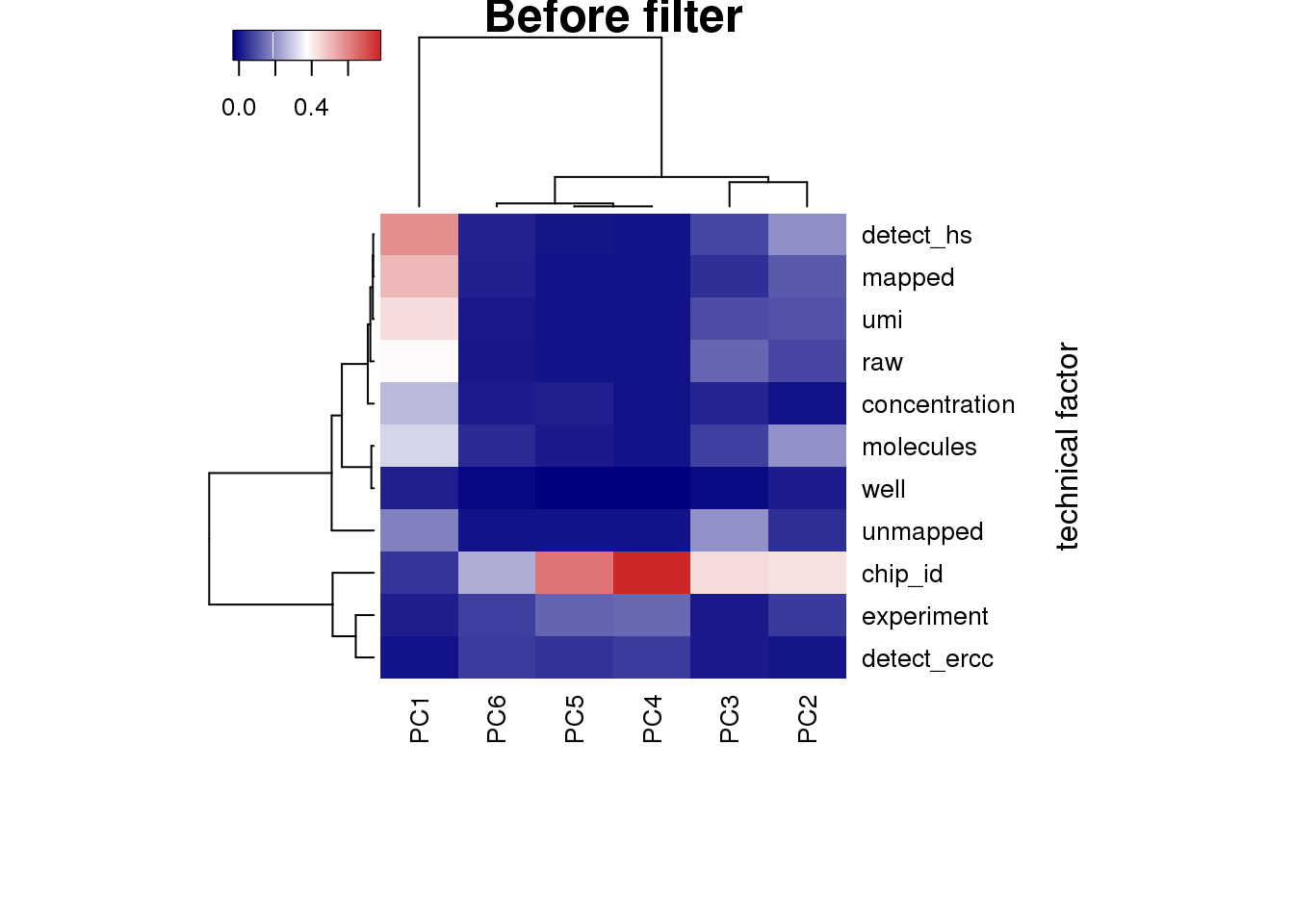

Supplement: Supplemental Material [file supp_gr.247759.118_Supplemental_peco-paper-master-source-code.tar.gz › peco-paper-master/docs/figure/pca_tf.Rmd/before-filter-1.png]

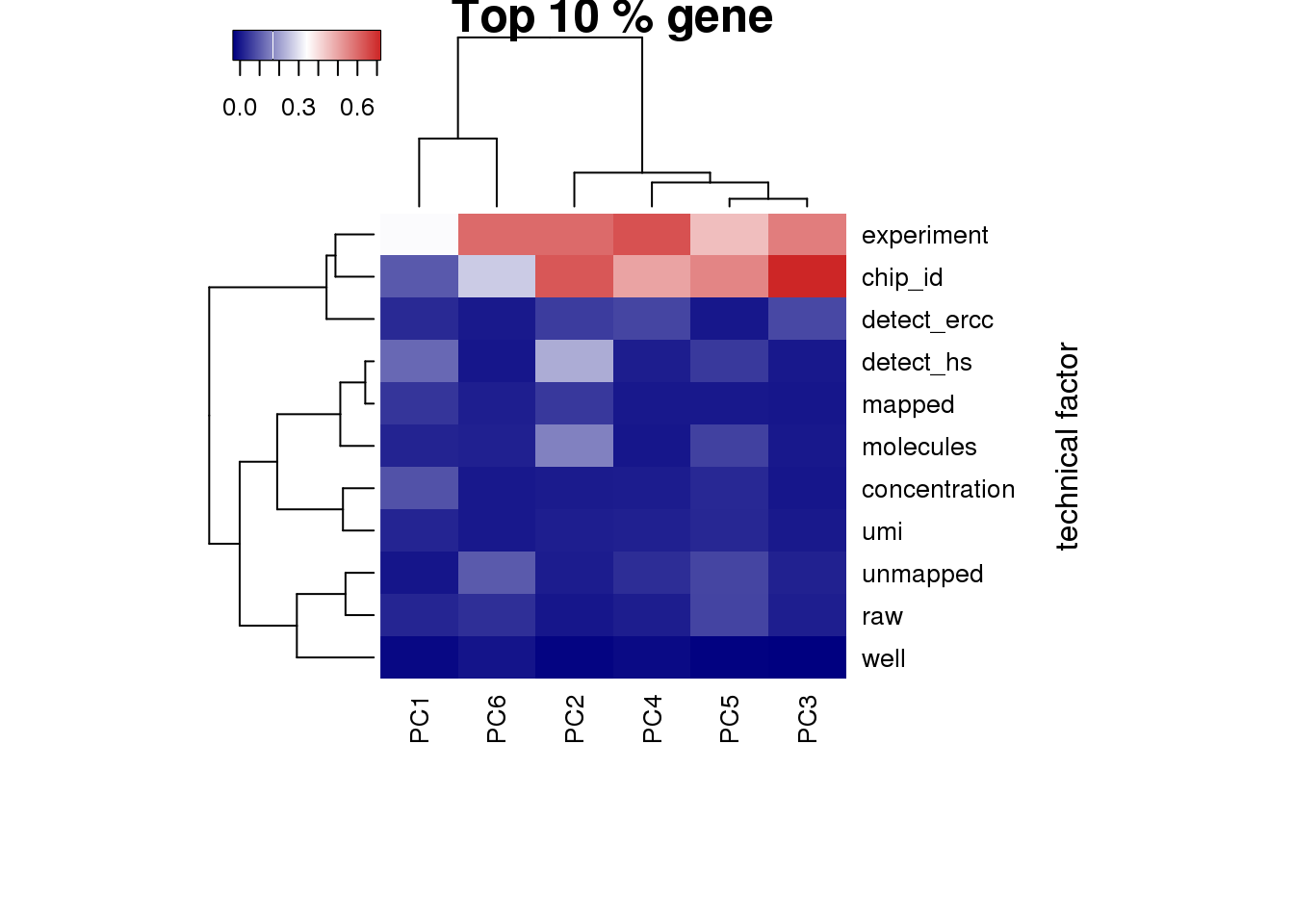

Supplement: Supplemental Material [file supp_gr.247759.118_Supplemental_peco-paper-master-source-code.tar.gz › peco-paper-master/docs/figure/pca_tf.Rmd/top-1.png]

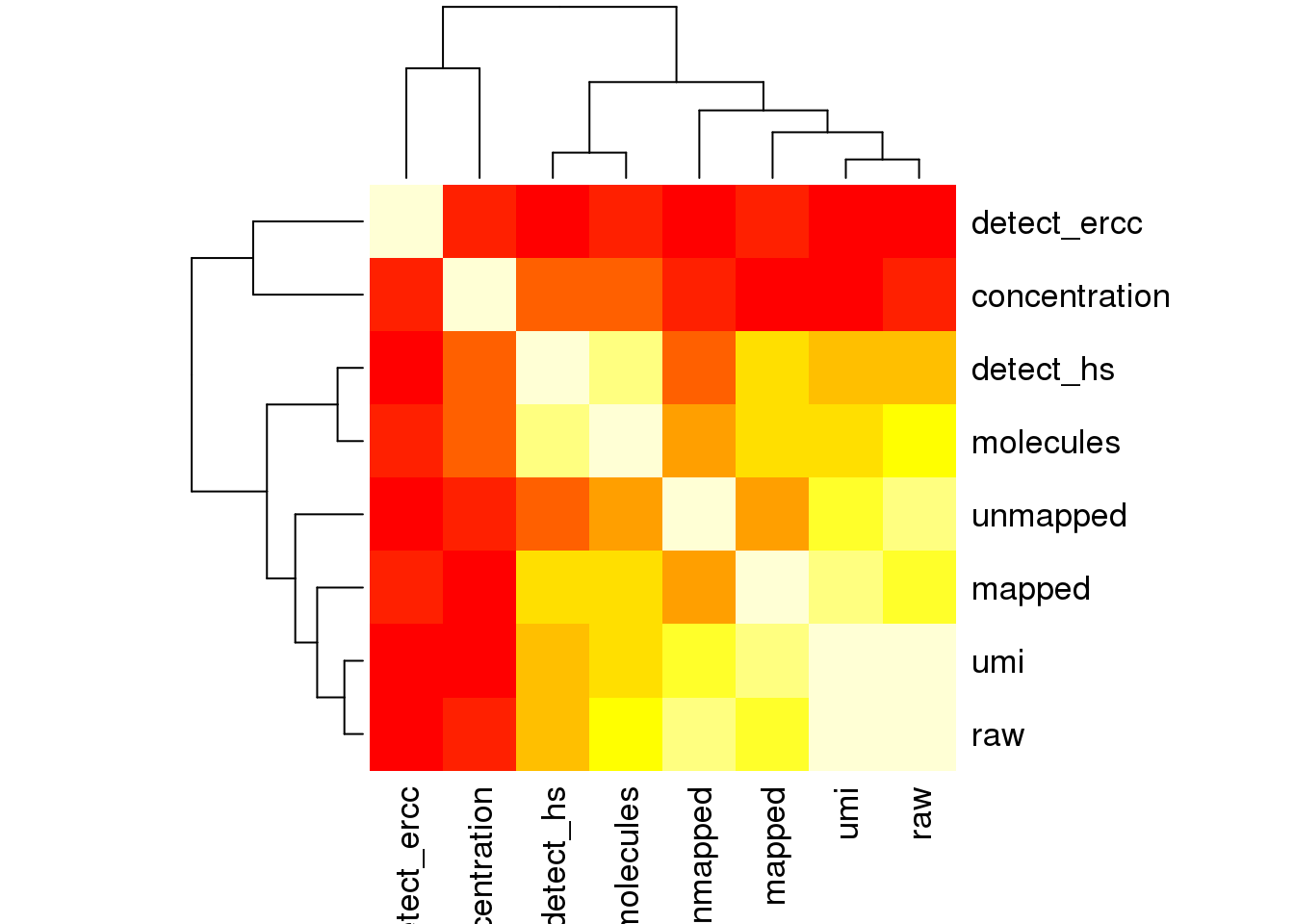

Supplement: Supplemental Material [file supp_gr.247759.118_Supplemental_peco-paper-master-source-code.tar.gz › peco-paper-master/docs/figure/pca_tf.Rmd/cor-1.png]

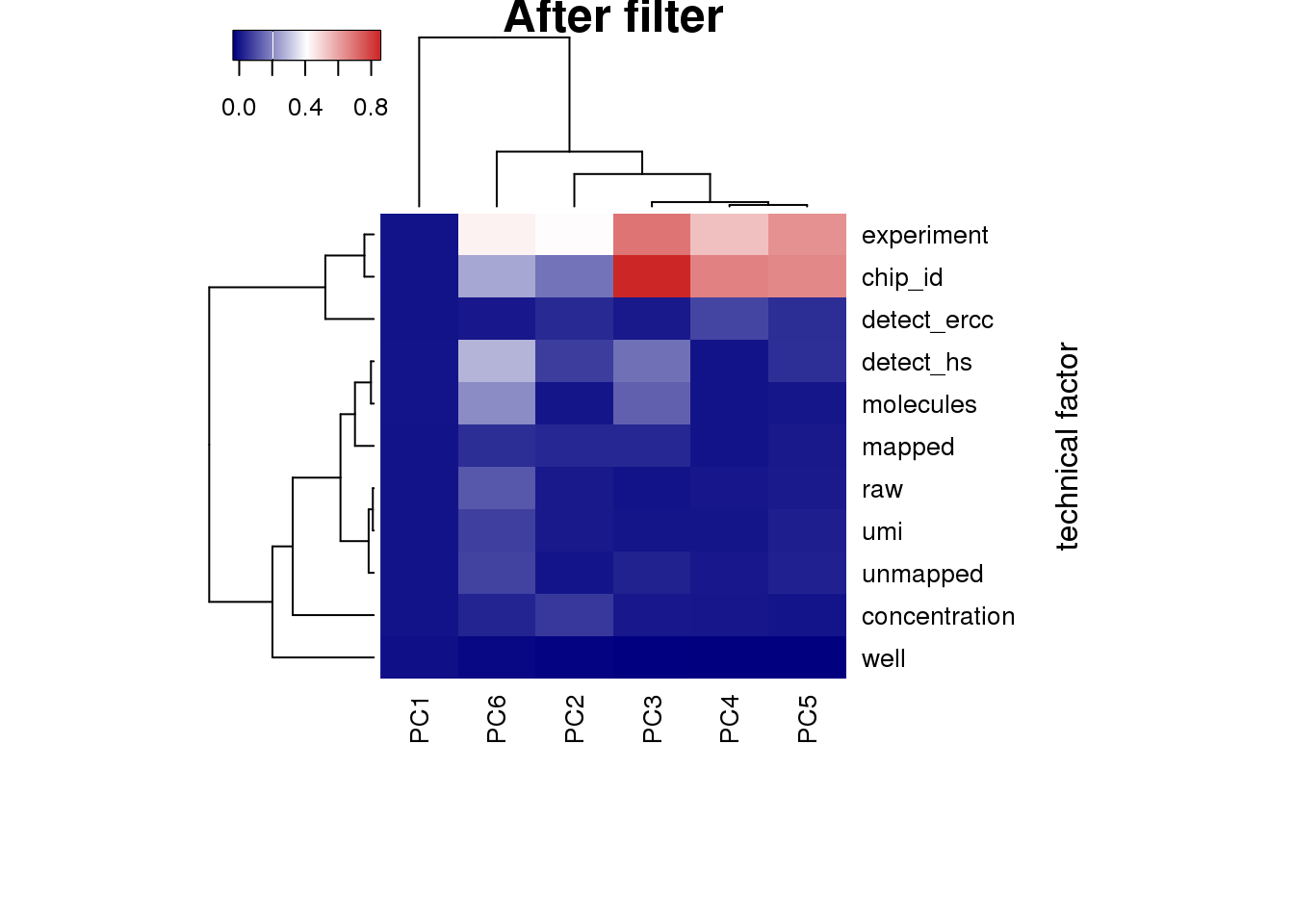

Supplement: Supplemental Material [file supp_gr.247759.118_Supplemental_peco-paper-master-source-code.tar.gz › peco-paper-master/docs/figure/pca_tf.Rmd/after-filter-tf-1.png]

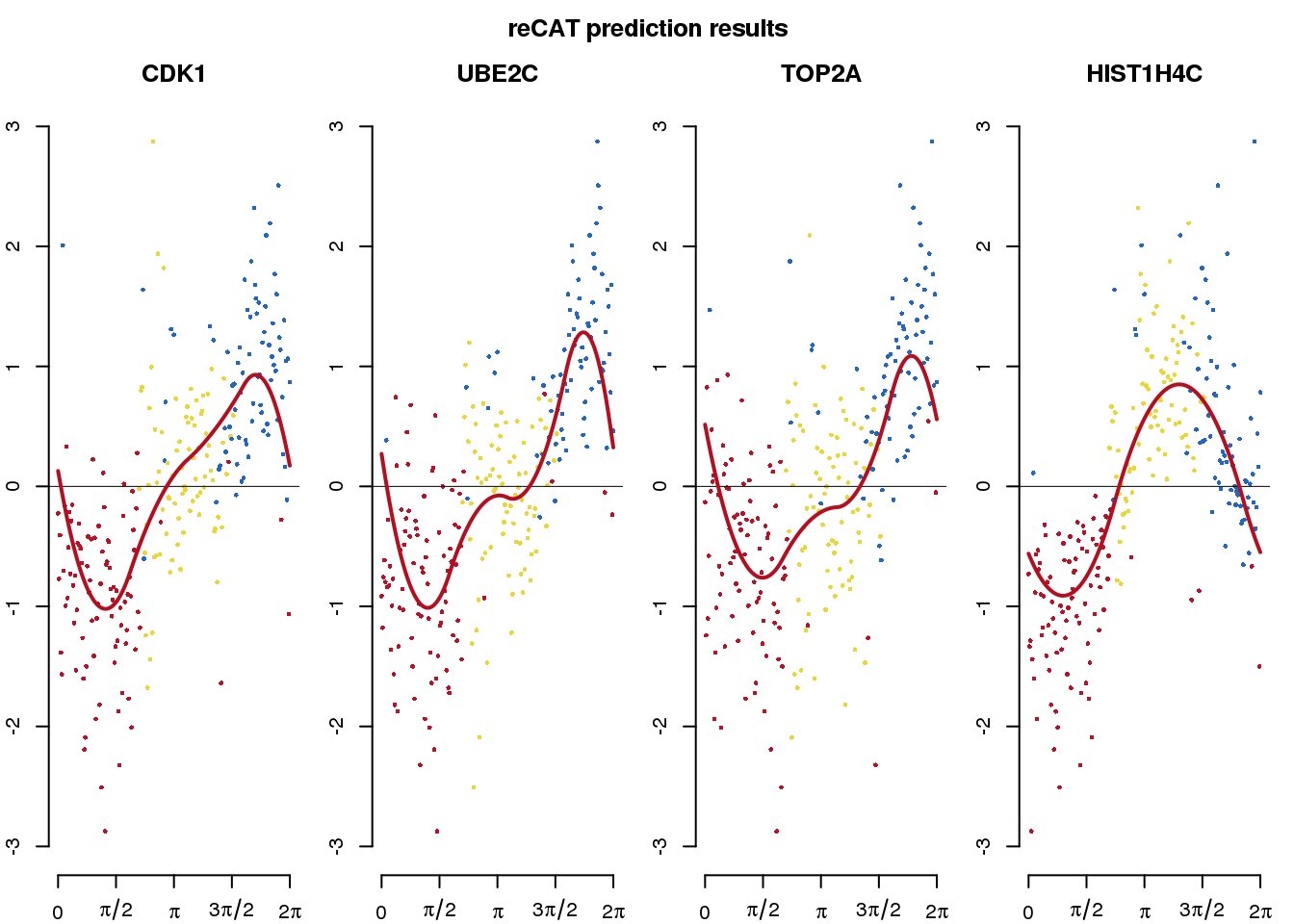

Supplement: Supplemental Material [file supp_gr.247759.118_Supplemental_peco-paper-master-source-code.tar.gz › peco-paper-master/docs/figure/eval_on_leng2015_data.Rmd/plot-recar-1.png]

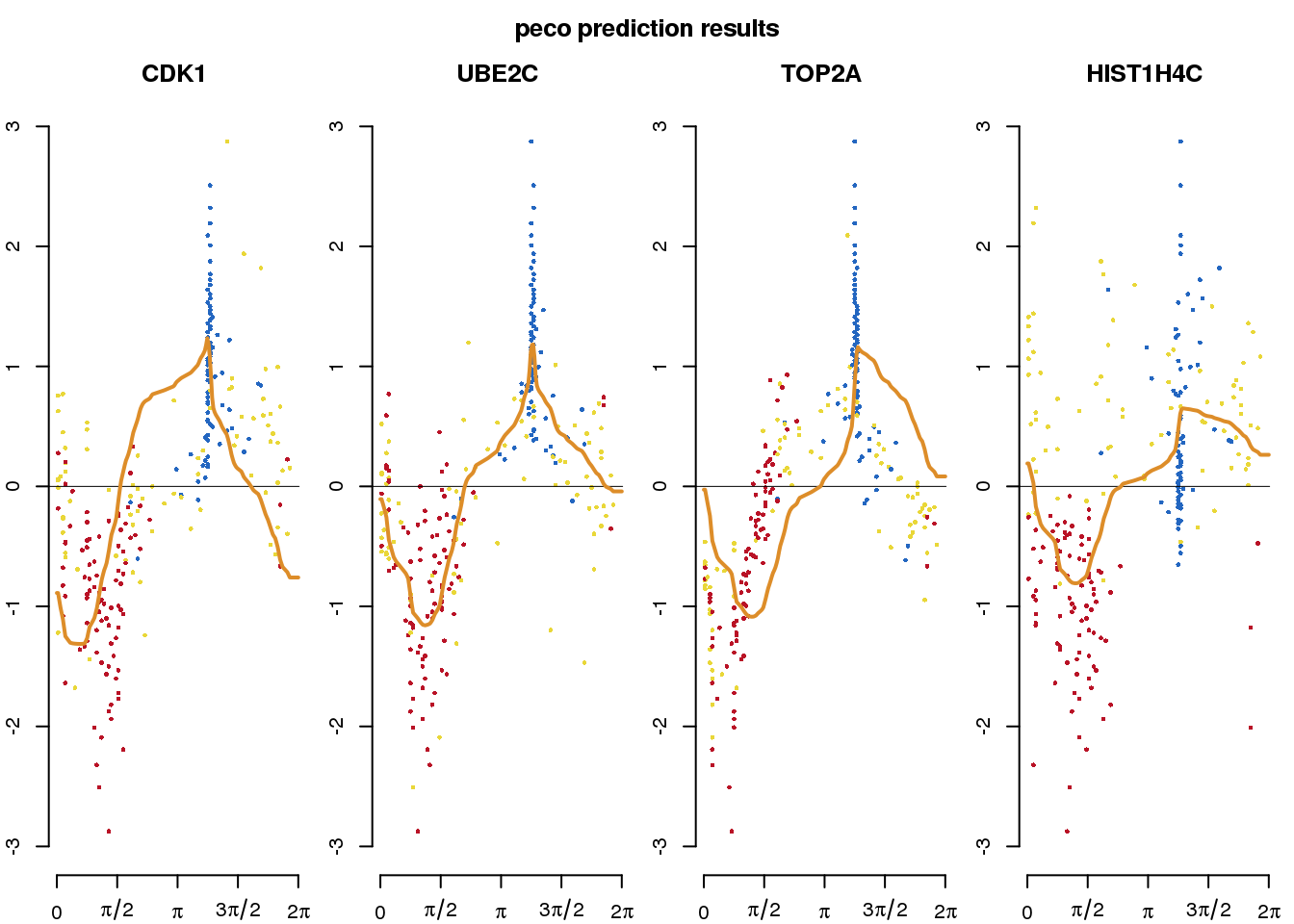

Supplement: Supplemental Material [file supp_gr.247759.118_Supplemental_peco-paper-master-source-code.tar.gz › peco-paper-master/docs/figure/eval_on_leng2015_data.Rmd/peco-2.png]

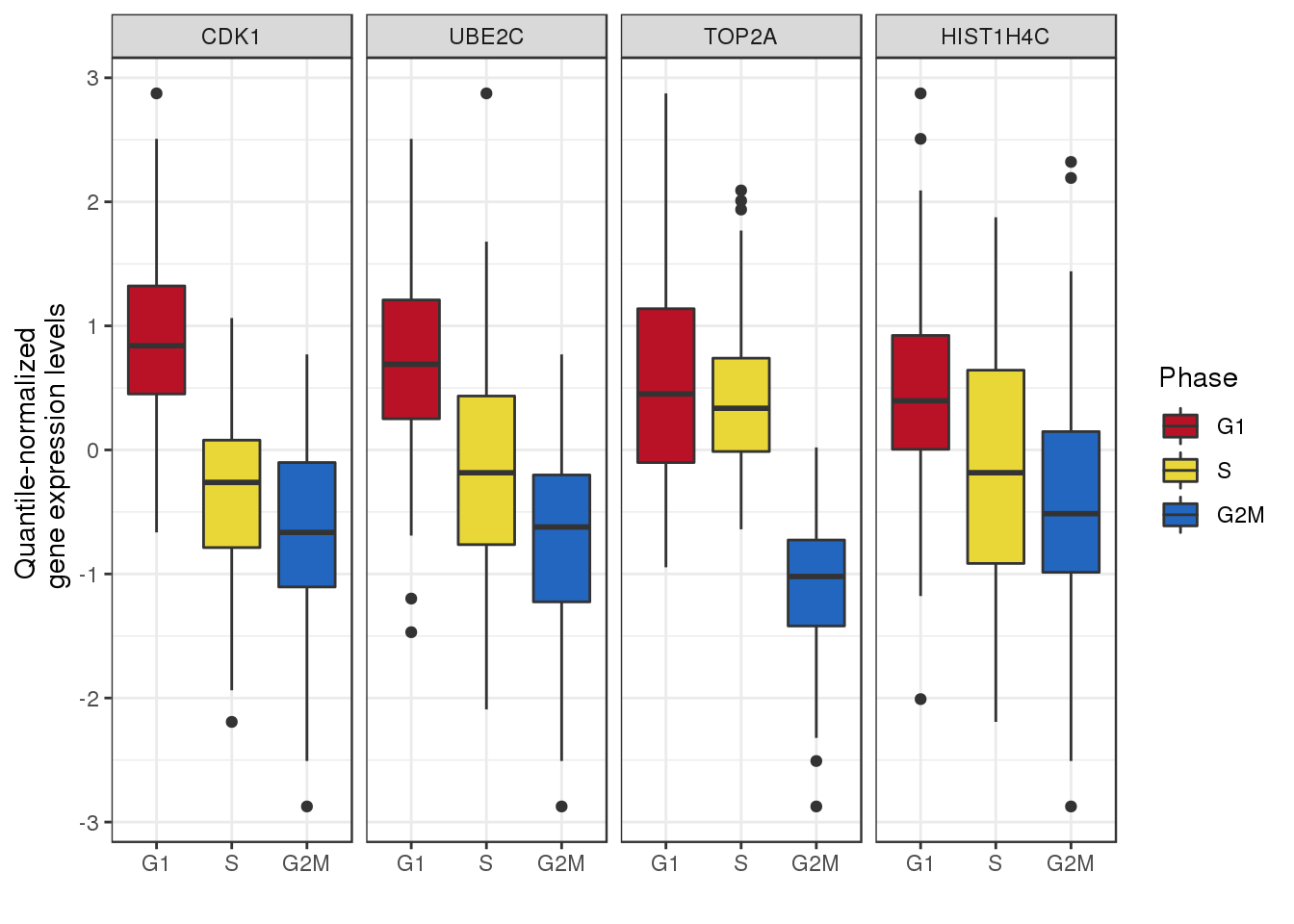

Supplement: Supplemental Material [file supp_gr.247759.118_Supplemental_peco-paper-master-source-code.tar.gz › peco-paper-master/docs/figure/eval_on_leng2015_data.Rmd/peco-1.png]

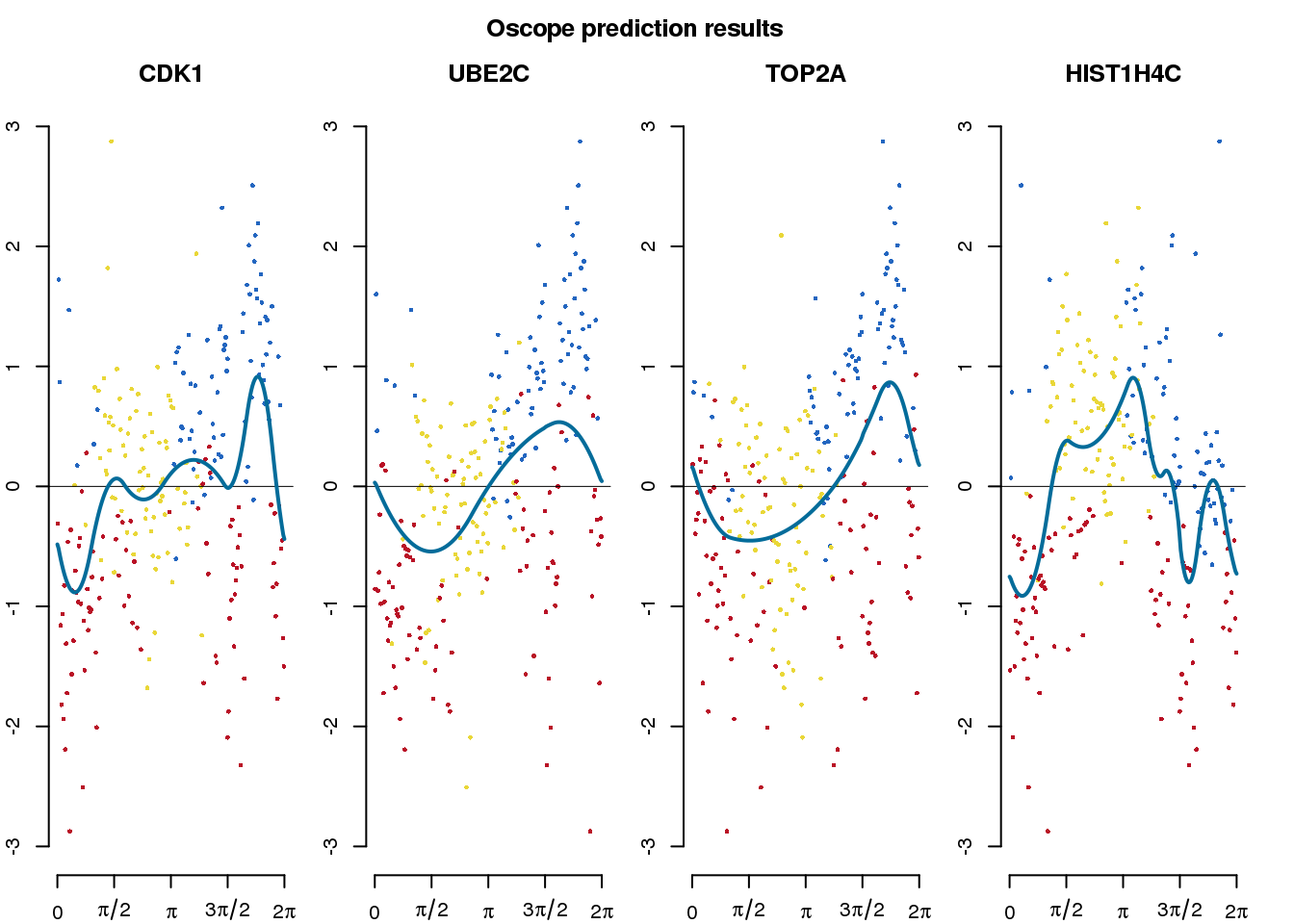

Supplement: Supplemental Material [file supp_gr.247759.118_Supplemental_peco-paper-master-source-code.tar.gz › peco-paper-master/docs/figure/eval_on_leng2015_data.Rmd/plot-oscope-1.png]

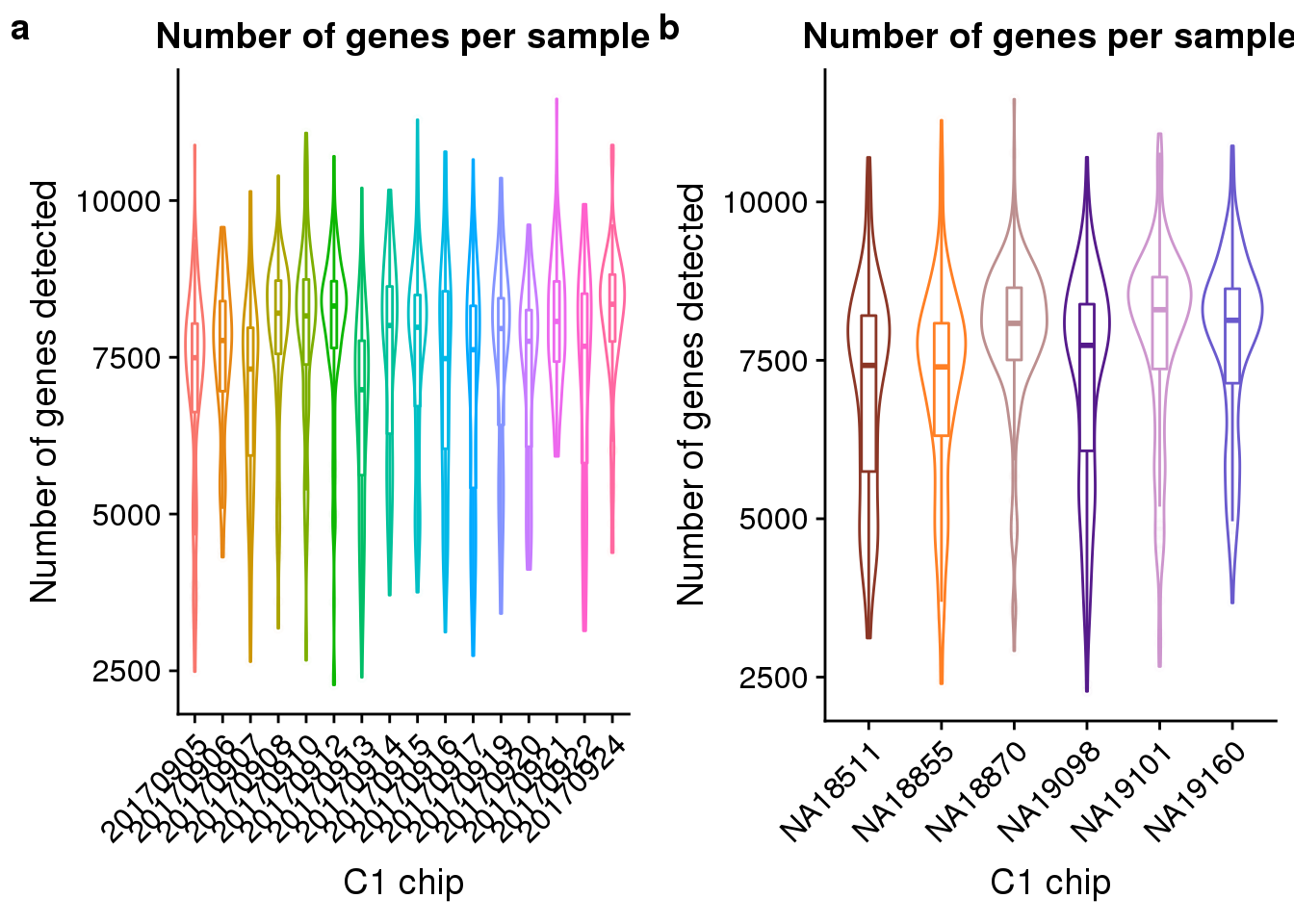

Supplement: Supplemental Material [file supp_gr.247759.118_Supplemental_peco-paper-master-source-code.tar.gz › peco-paper-master/docs/figure/sampleqc.Rmd/gene-number-exp-1.png]

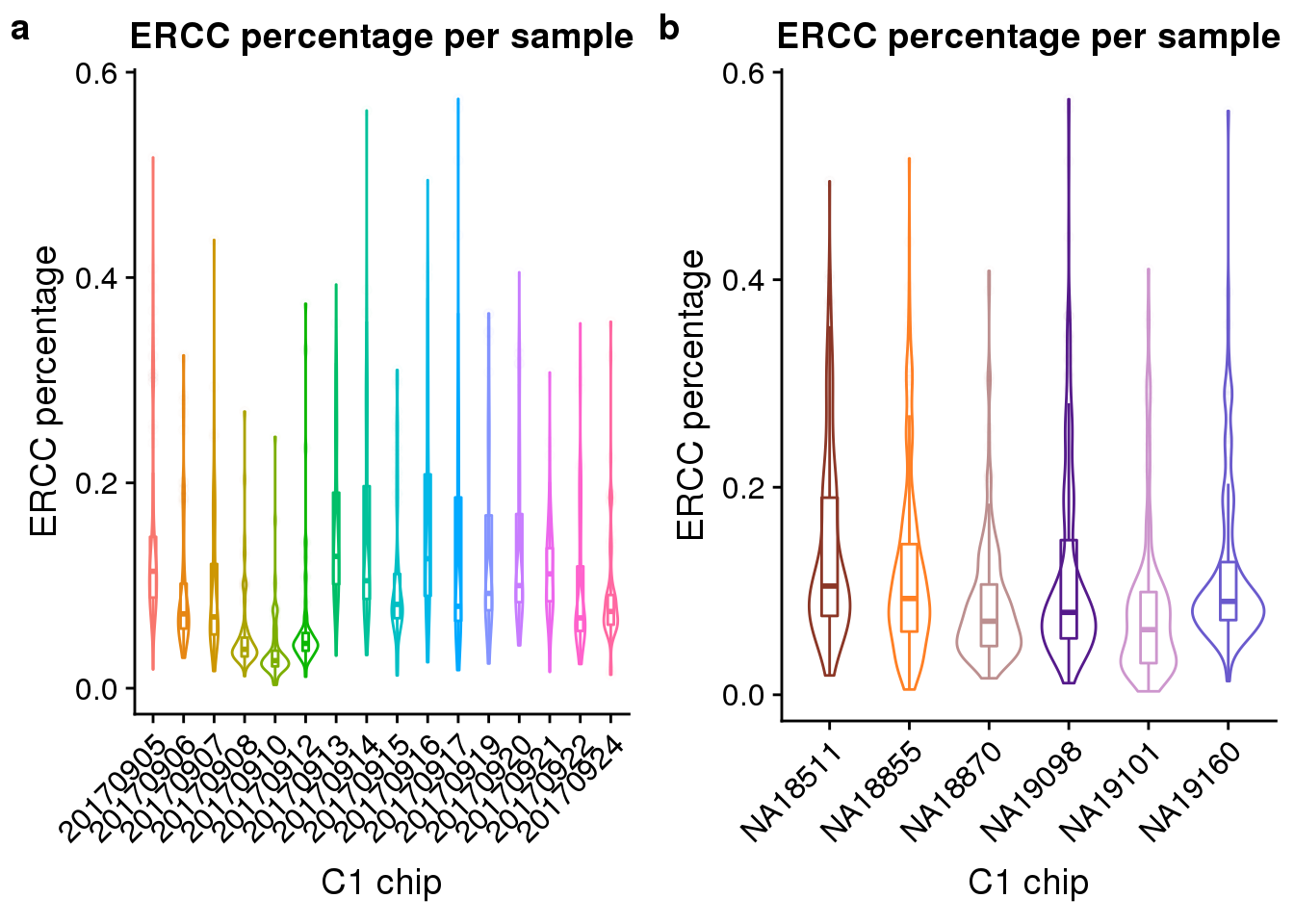

Supplement: Supplemental Material [file supp_gr.247759.118_Supplemental_peco-paper-master-source-code.tar.gz › peco-paper-master/docs/figure/sampleqc.Rmd/ercc_exp-1.png]

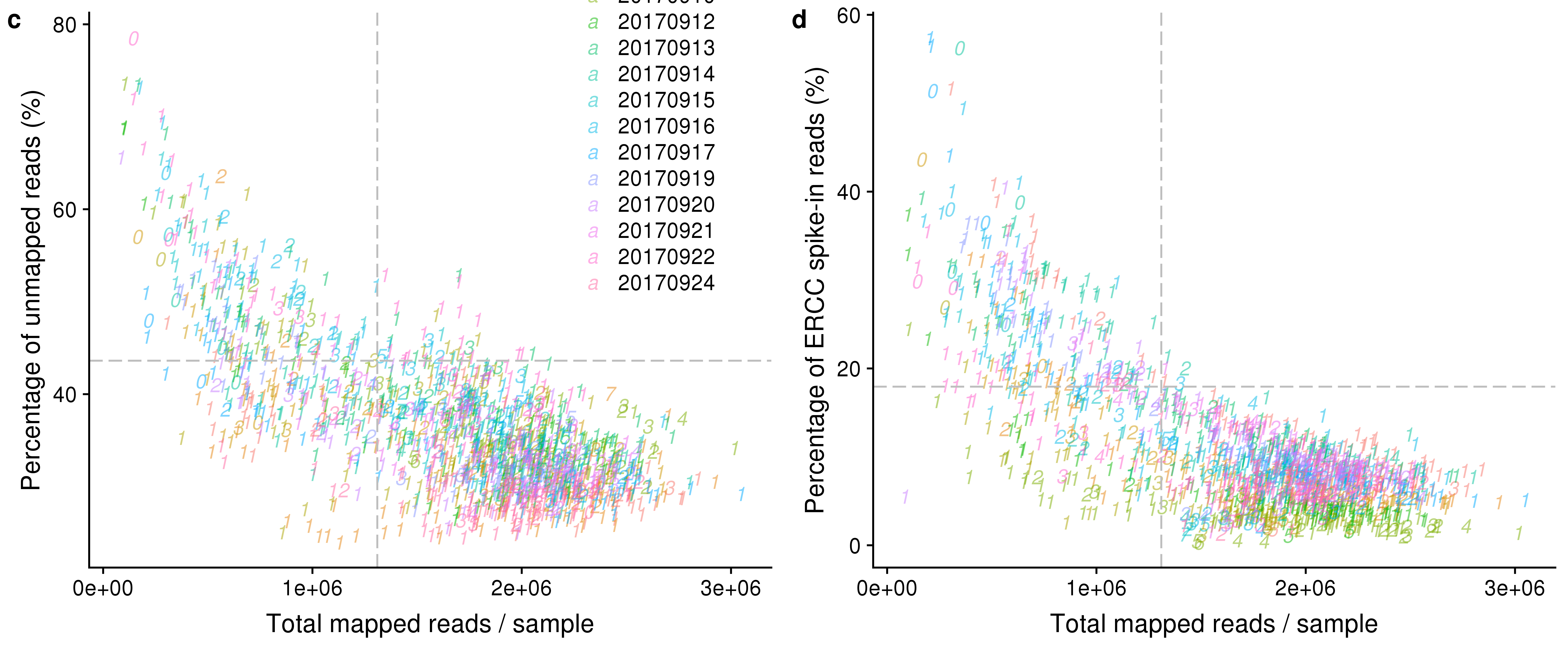

Supplement: Supplemental Material [file supp_gr.247759.118_Supplemental_peco-paper-master-source-code.tar.gz › peco-paper-master/docs/figure/sampleqc.Rmd/plots-2.png]

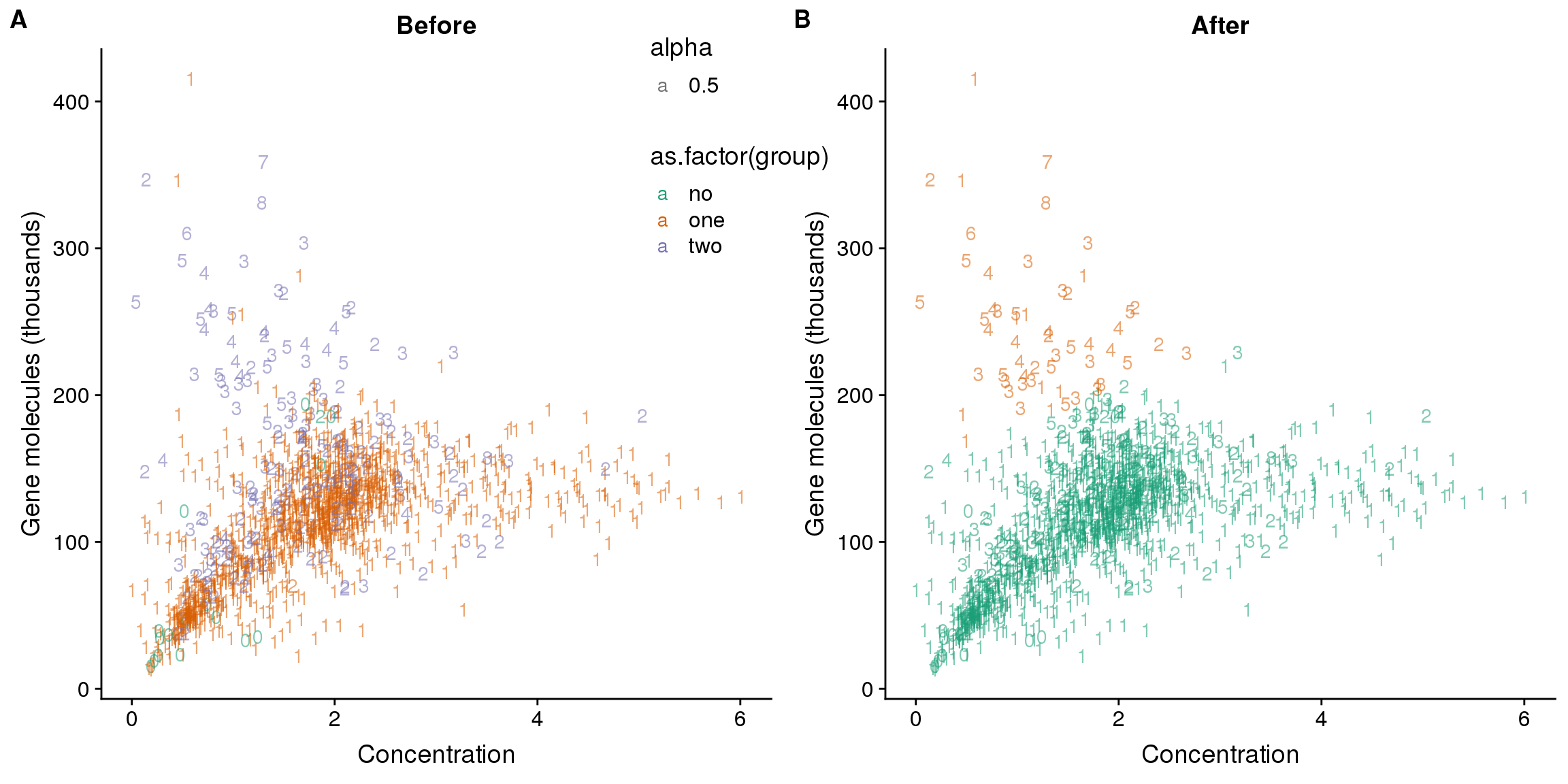

Supplement: Supplemental Material [file supp_gr.247759.118_Supplemental_peco-paper-master-source-code.tar.gz › peco-paper-master/docs/figure/sampleqc.Rmd/lda-1.png]

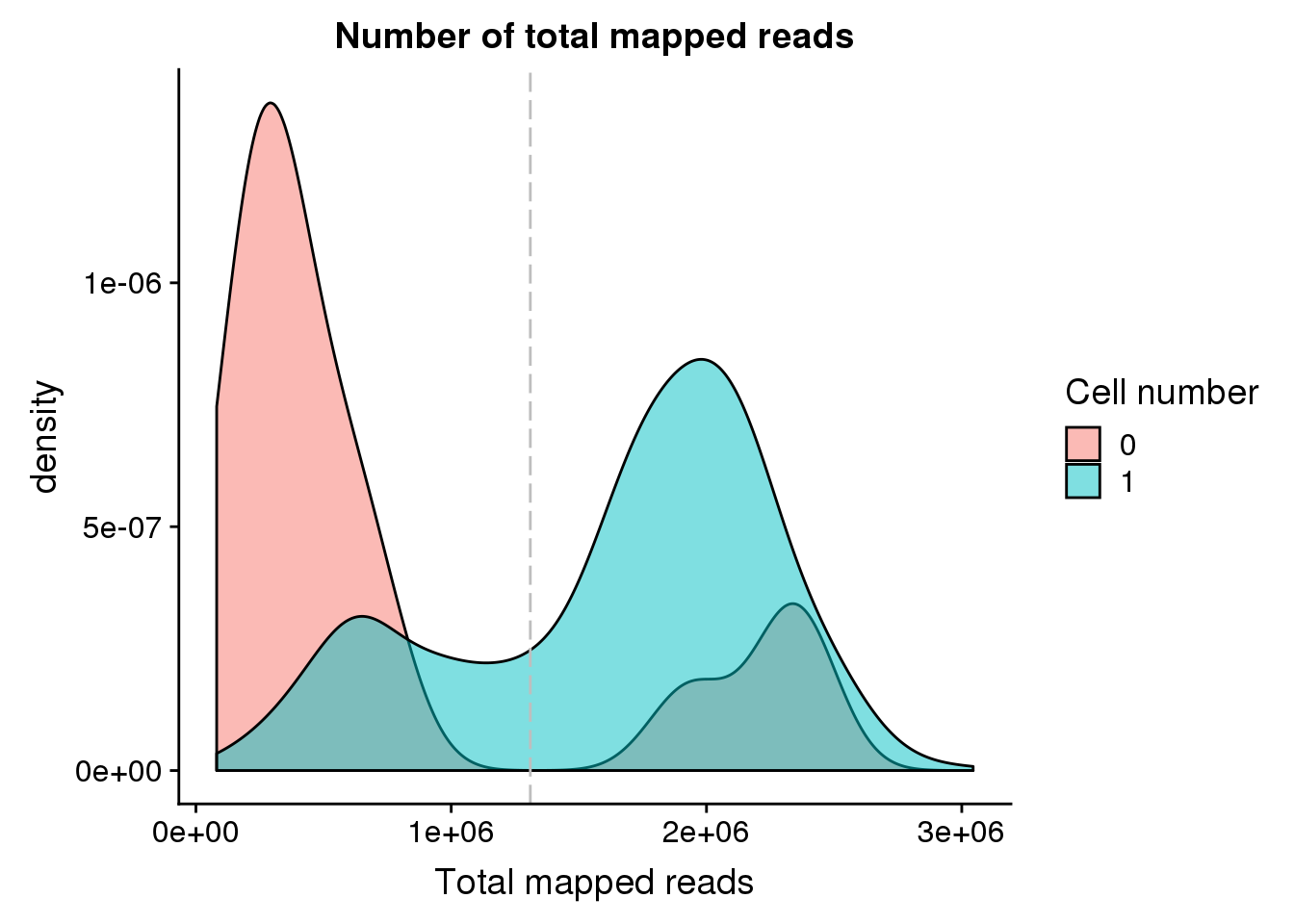

Supplement: Supplemental Material [file supp_gr.247759.118_Supplemental_peco-paper-master-source-code.tar.gz › peco-paper-master/docs/figure/sampleqc.Rmd/total-reads-1.png]

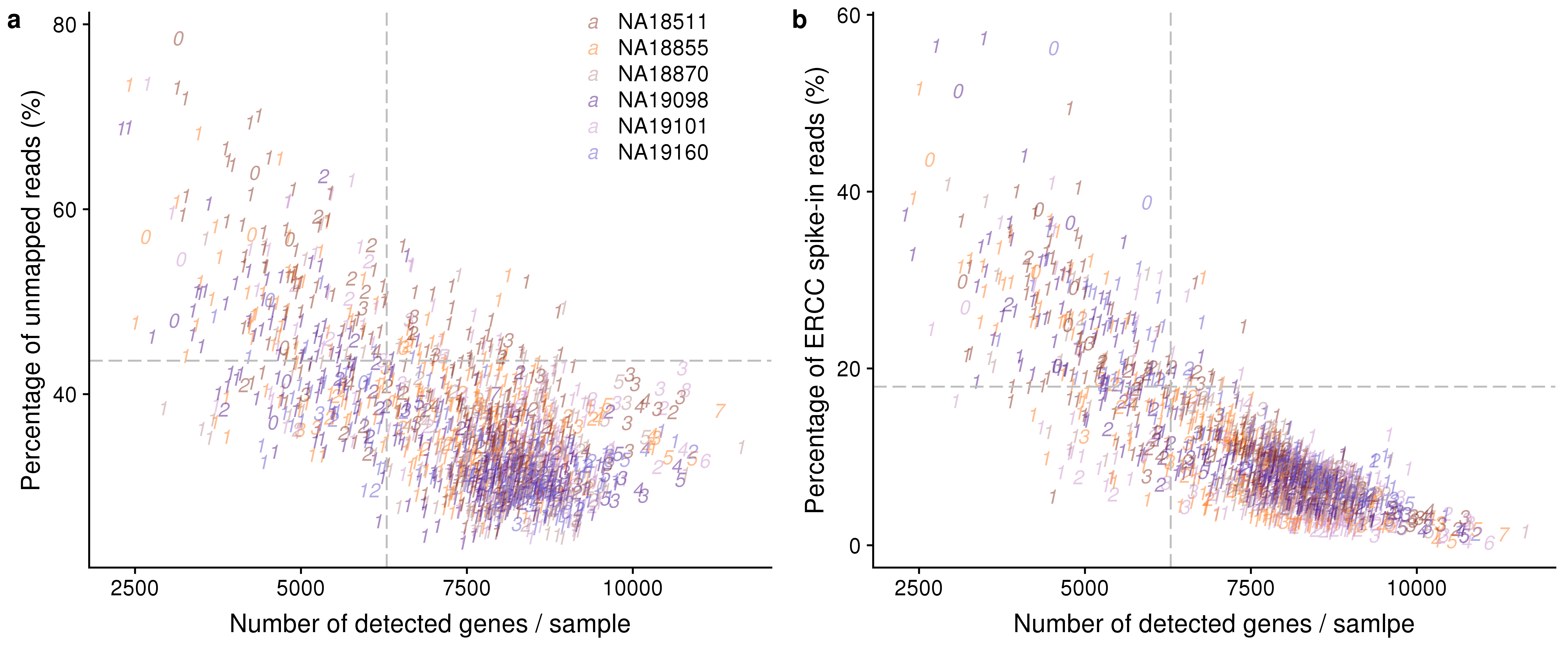

Supplement: Supplemental Material [file supp_gr.247759.118_Supplemental_peco-paper-master-source-code.tar.gz › peco-paper-master/docs/figure/sampleqc.Rmd/plots-1.png]

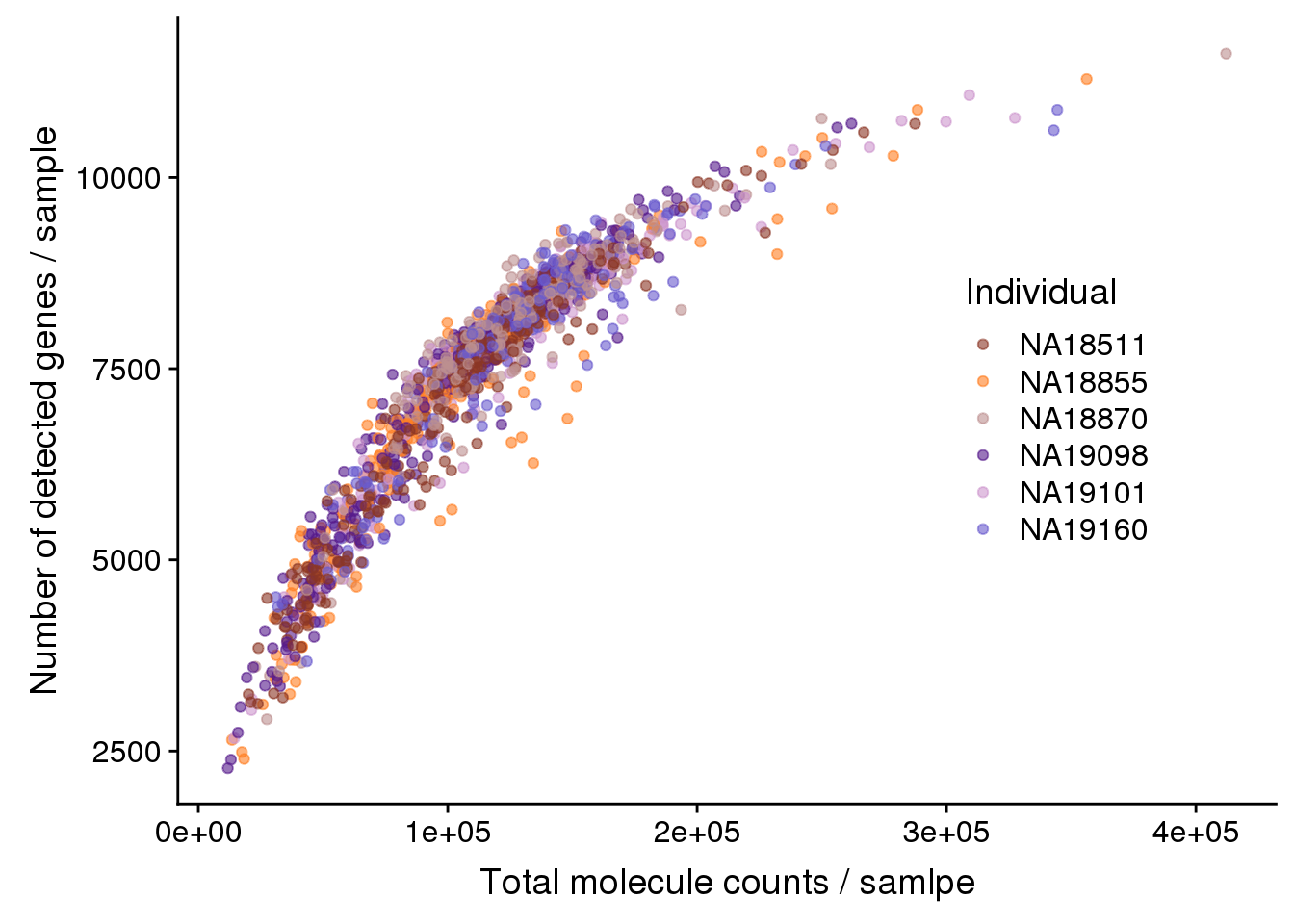

Supplement: Supplemental Material [file supp_gr.247759.118_Supplemental_peco-paper-master-source-code.tar.gz › peco-paper-master/docs/figure/sampleqc.Rmd/unnamed-chunk-1-1.png]

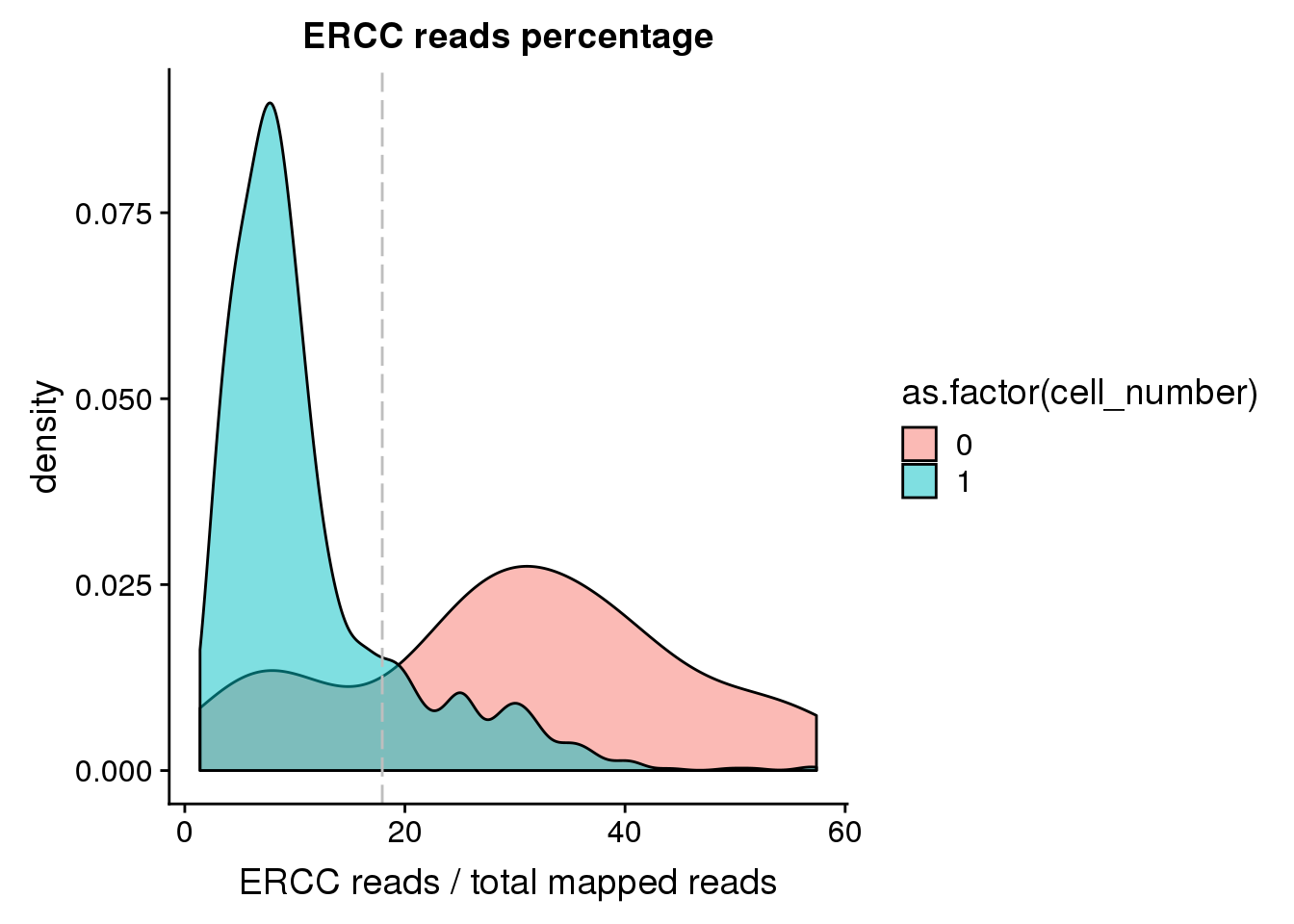

Supplement: Supplemental Material [file supp_gr.247759.118_Supplemental_peco-paper-master-source-code.tar.gz › peco-paper-master/docs/figure/sampleqc.Rmd/ercc-percentage-1.png]

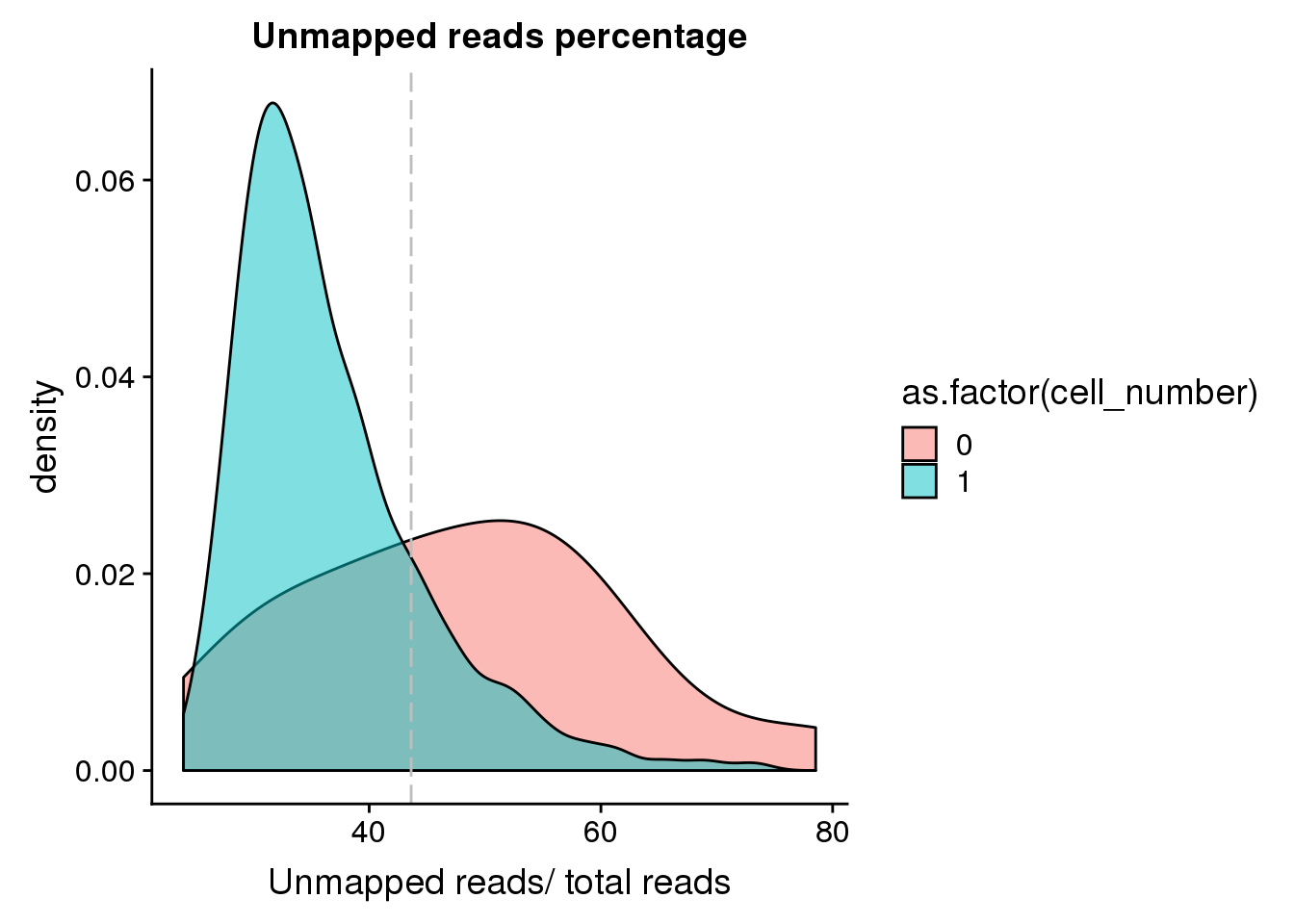

Supplement: Supplemental Material [file supp_gr.247759.118_Supplemental_peco-paper-master-source-code.tar.gz › peco-paper-master/docs/figure/sampleqc.Rmd/unmapped-ratios-1.png]

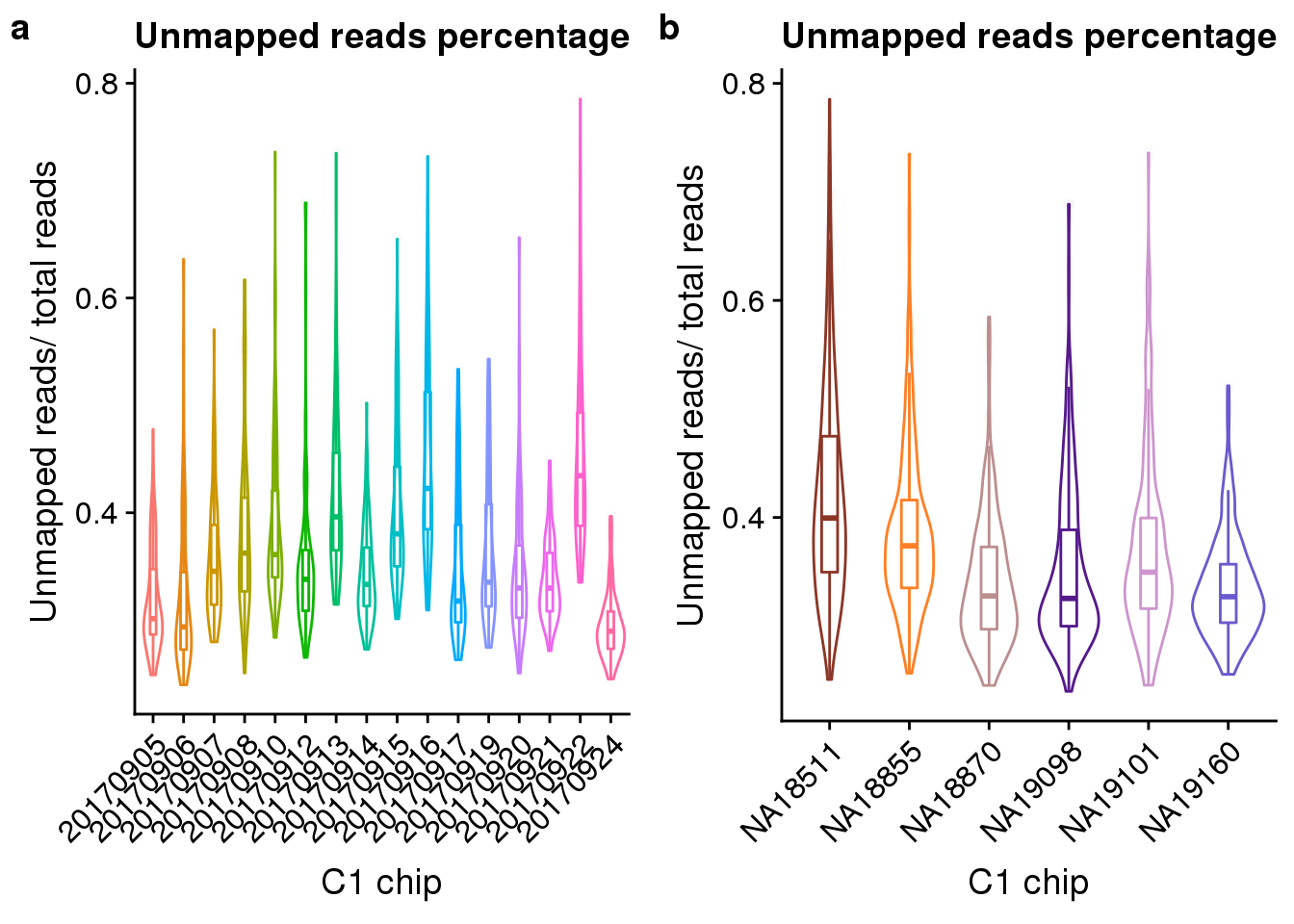

Supplement: Supplemental Material [file supp_gr.247759.118_Supplemental_peco-paper-master-source-code.tar.gz › peco-paper-master/docs/figure/sampleqc.Rmd/unmapped_exp-1.png]

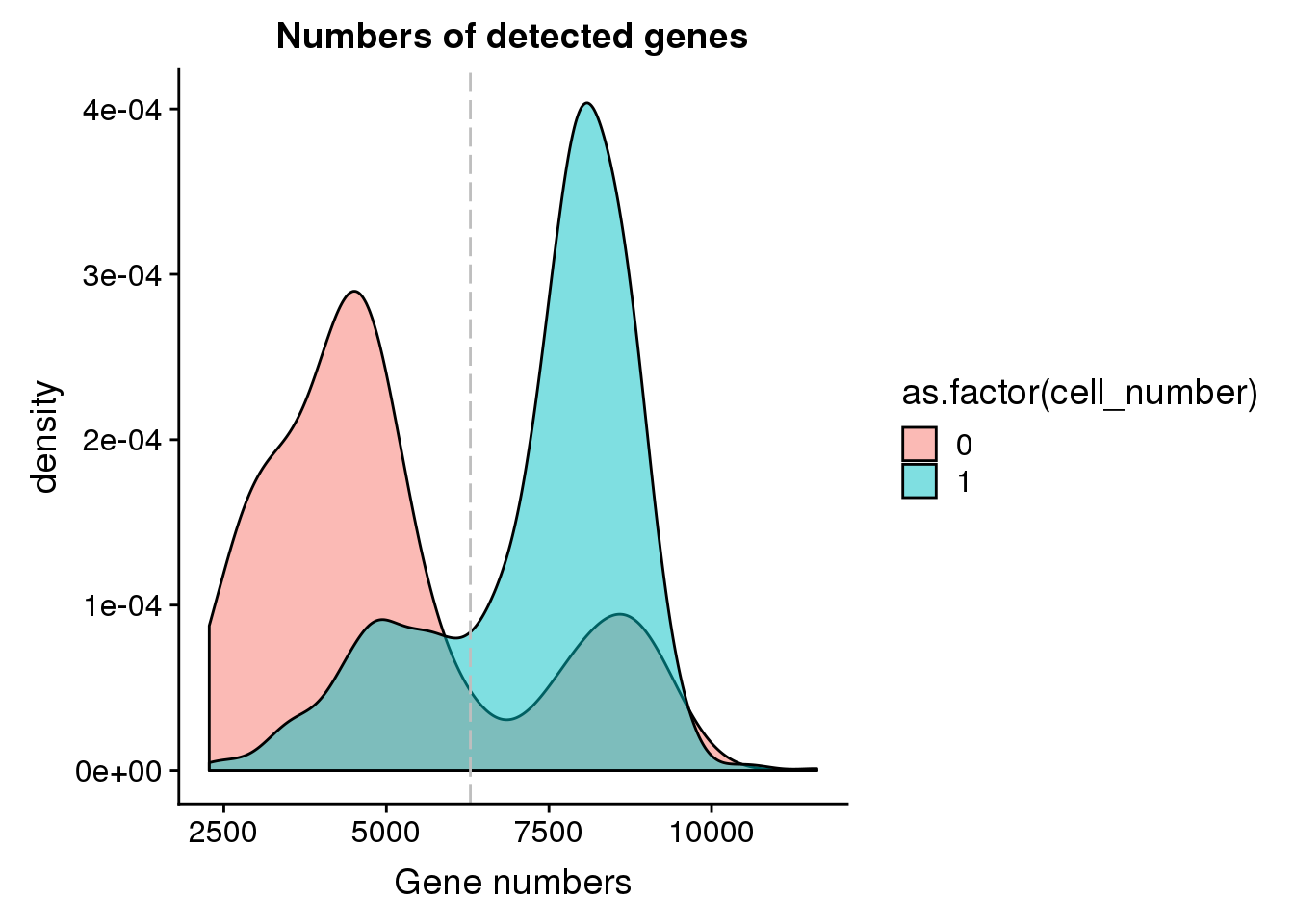

Supplement: Supplemental Material [file supp_gr.247759.118_Supplemental_peco-paper-master-source-code.tar.gz › peco-paper-master/docs/figure/sampleqc.Rmd/gene-number-1.png]

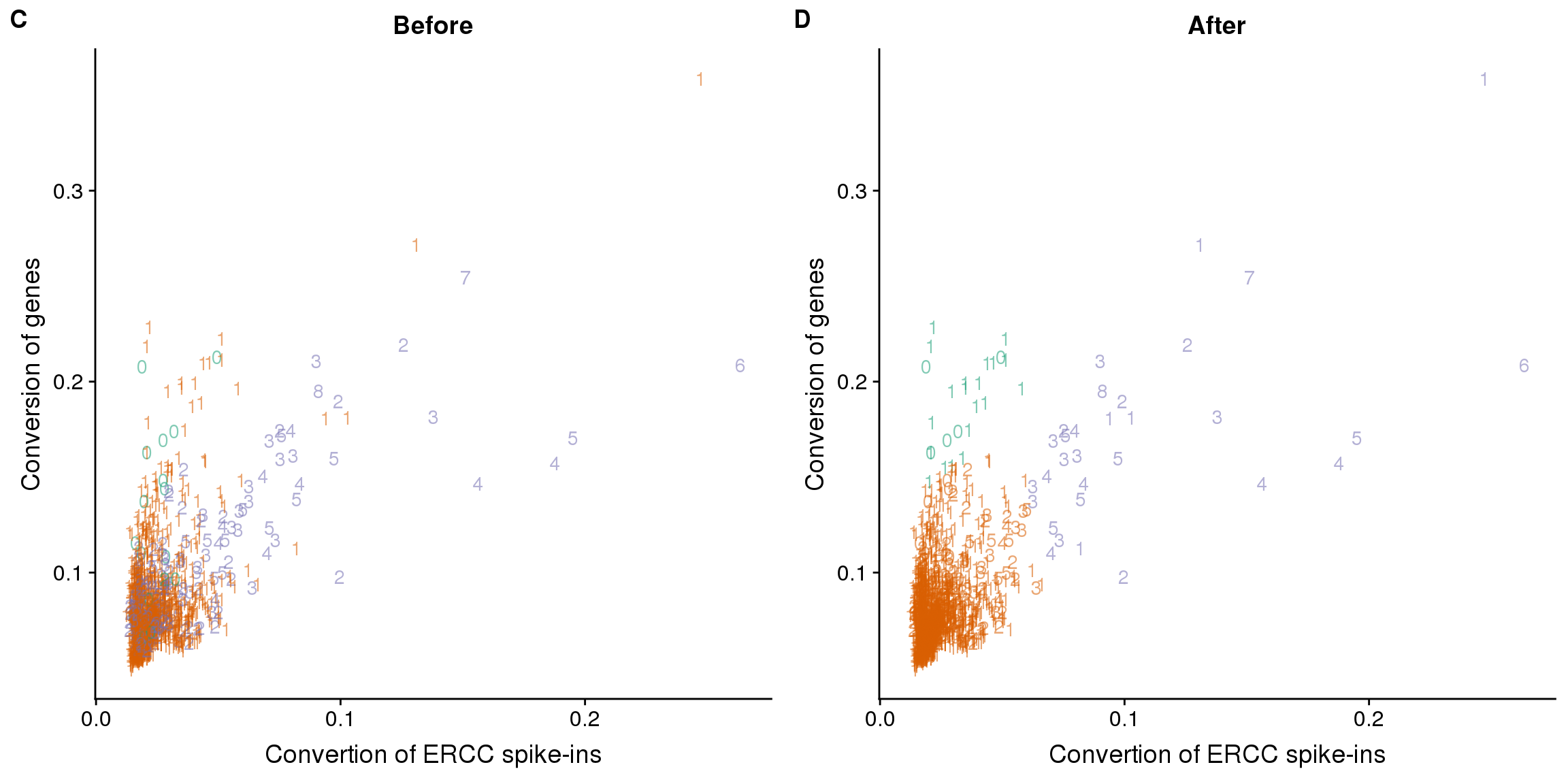

Supplement: Supplemental Material [file supp_gr.247759.118_Supplemental_peco-paper-master-source-code.tar.gz › peco-paper-master/docs/figure/sampleqc.Rmd/convertion-1.png]

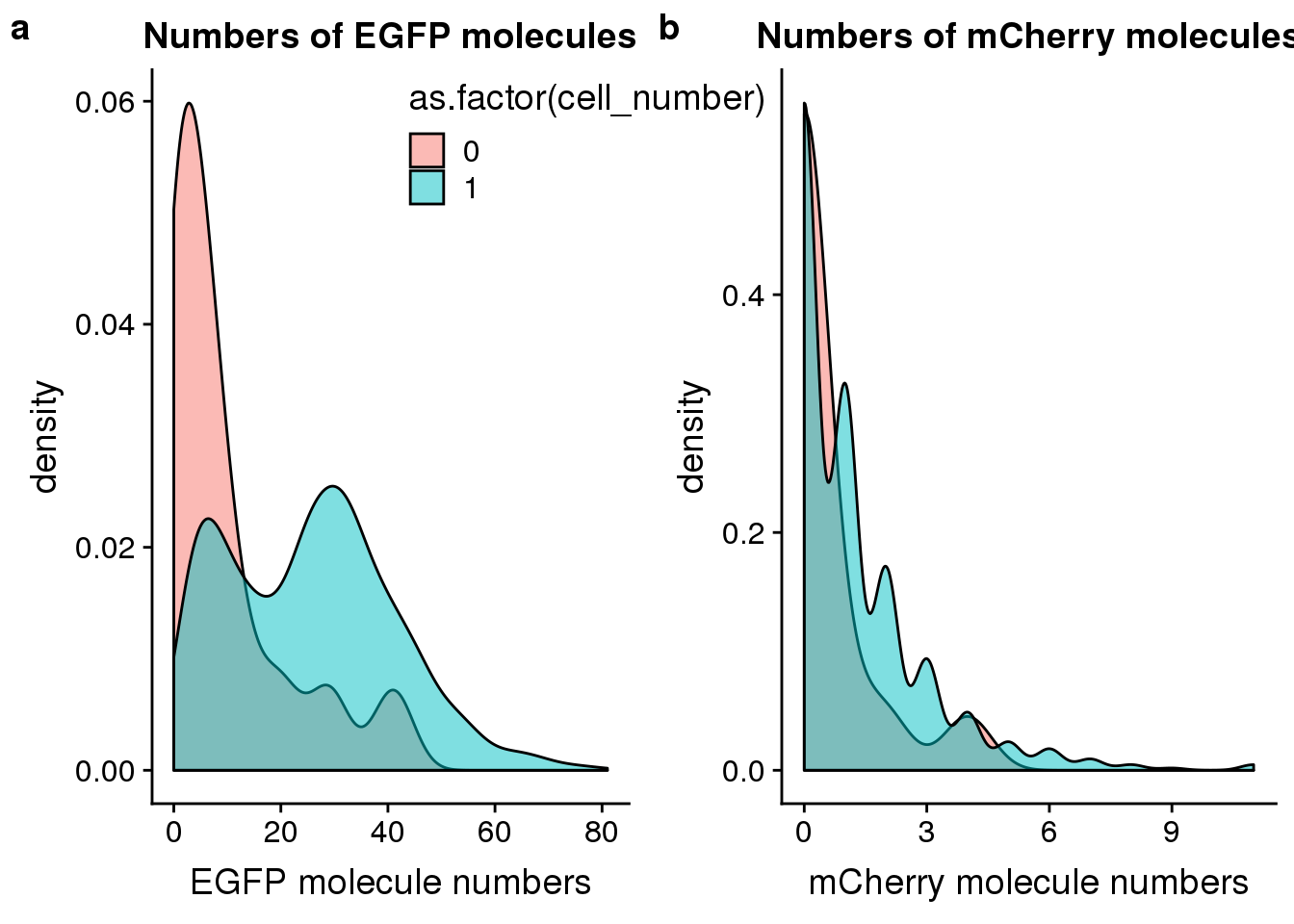

Supplement: Supplemental Material [file supp_gr.247759.118_Supplemental_peco-paper-master-source-code.tar.gz › peco-paper-master/docs/figure/sampleqc.Rmd/fucci-1.png]

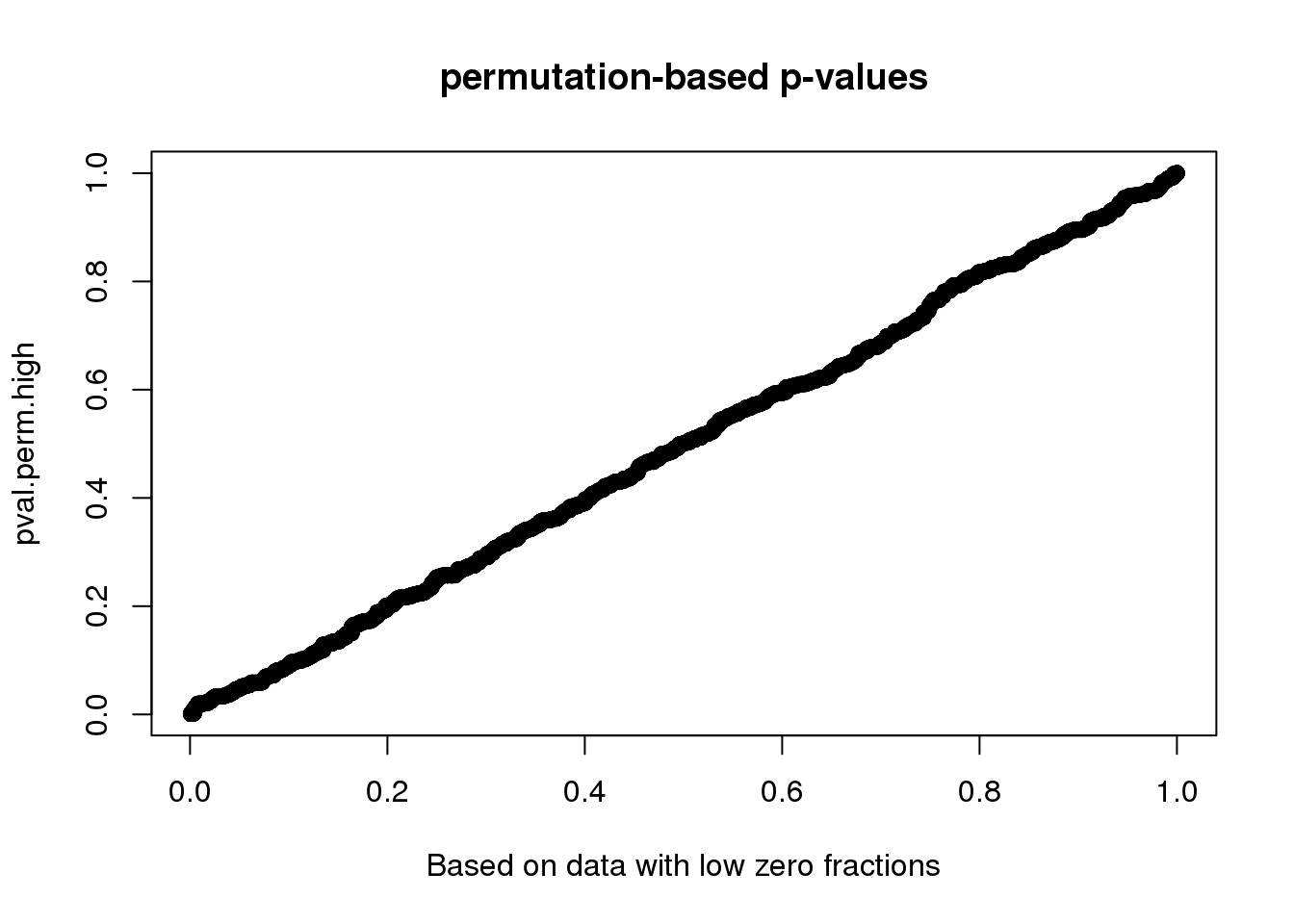

Supplement: Supplemental Material [file supp_gr.247759.118_Supplemental_peco-paper-master-source-code.tar.gz › peco-paper-master/docs/figure/npreg_trendfilter_quantile.Rmd/get-permute-pvals-1.png]

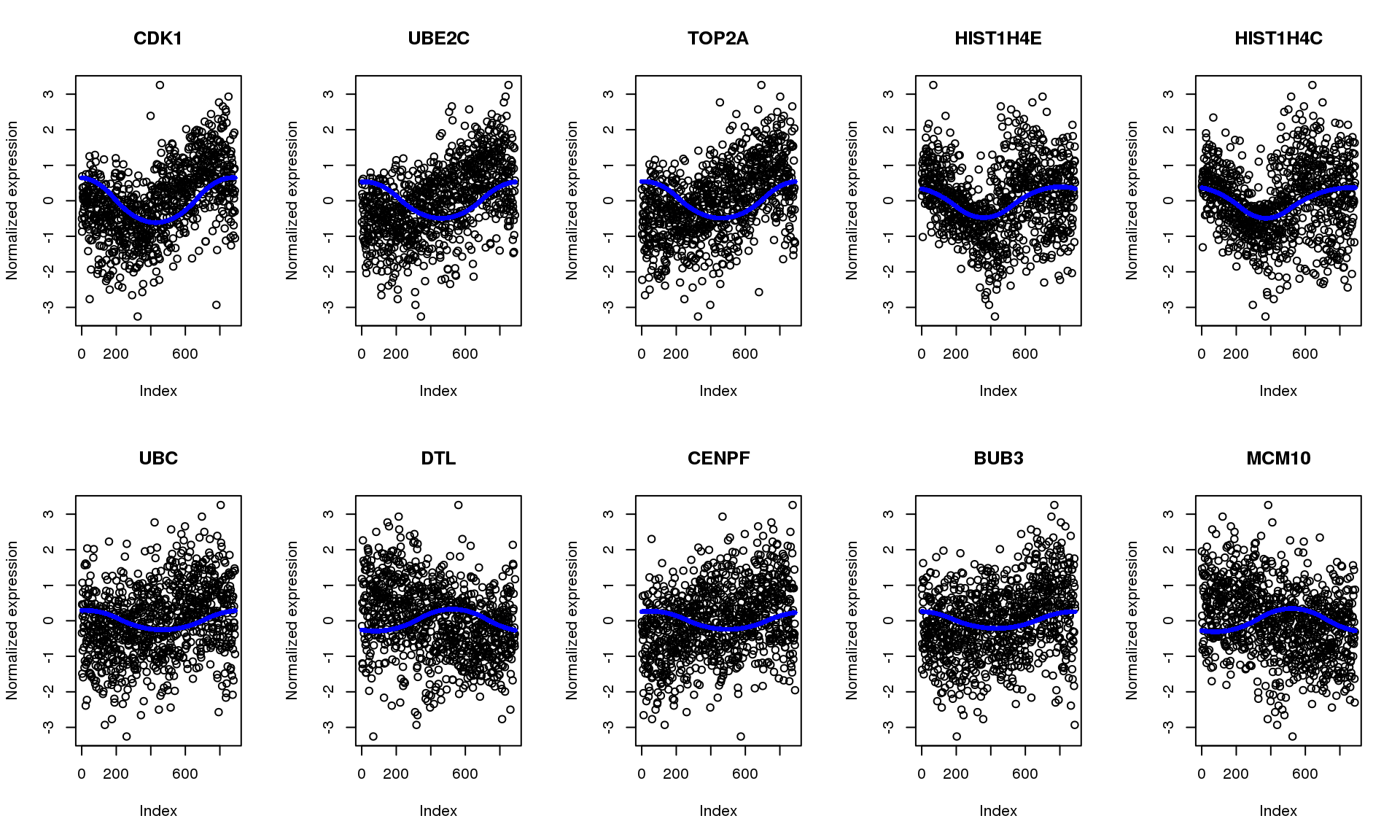

Supplement: Supplemental Material [file supp_gr.247759.118_Supplemental_peco-paper-master-source-code.tar.gz › peco-paper-master/docs/figure/npreg_trendfilter_quantile.Rmd/pve-top10-genes-1.png]

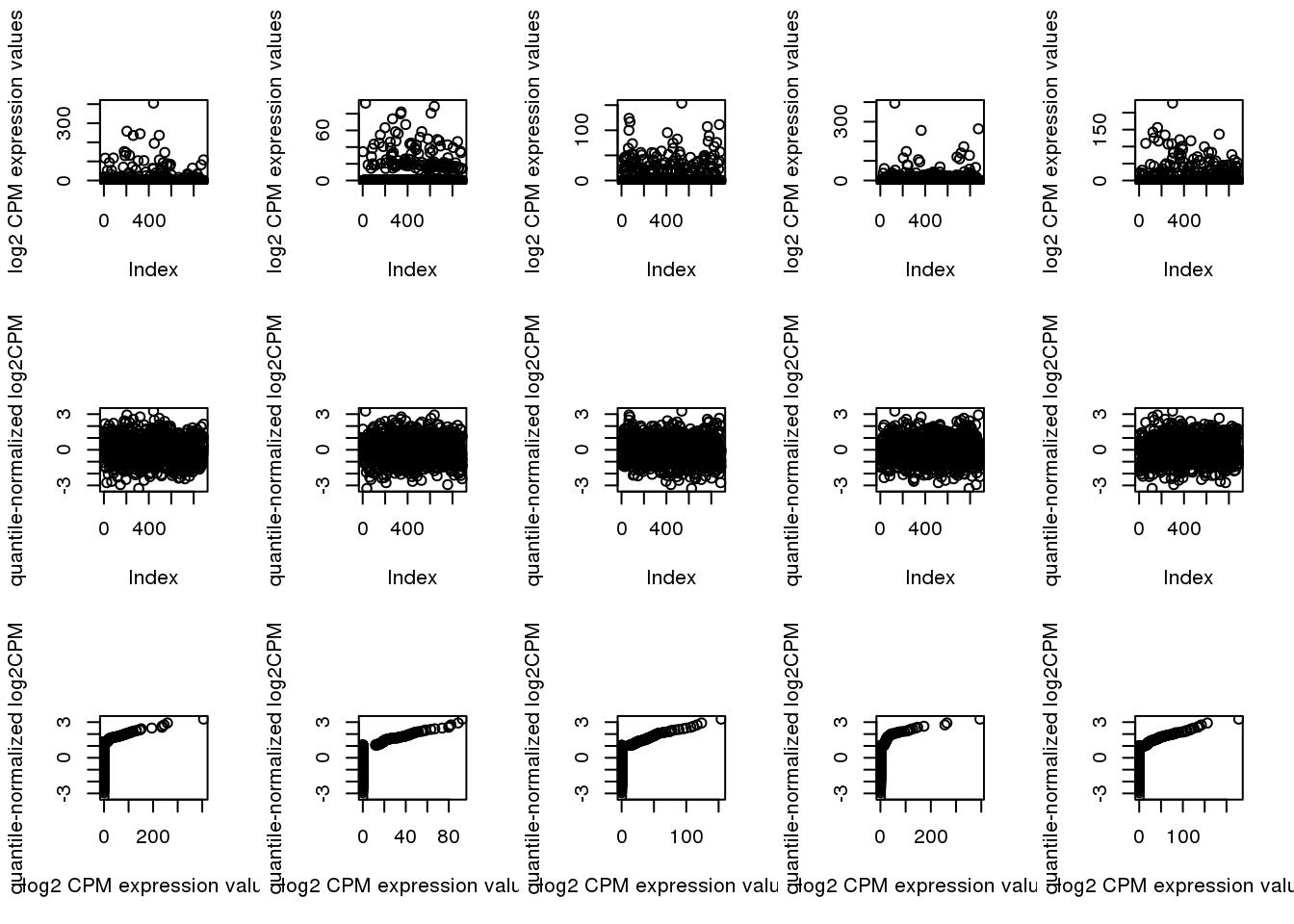

Supplement: Supplemental Material [file supp_gr.247759.118_Supplemental_peco-paper-master-source-code.tar.gz › peco-paper-master/docs/figure/npreg_trendfilter_quantile.Rmd/check-high-low-undetected-cells-1.png]

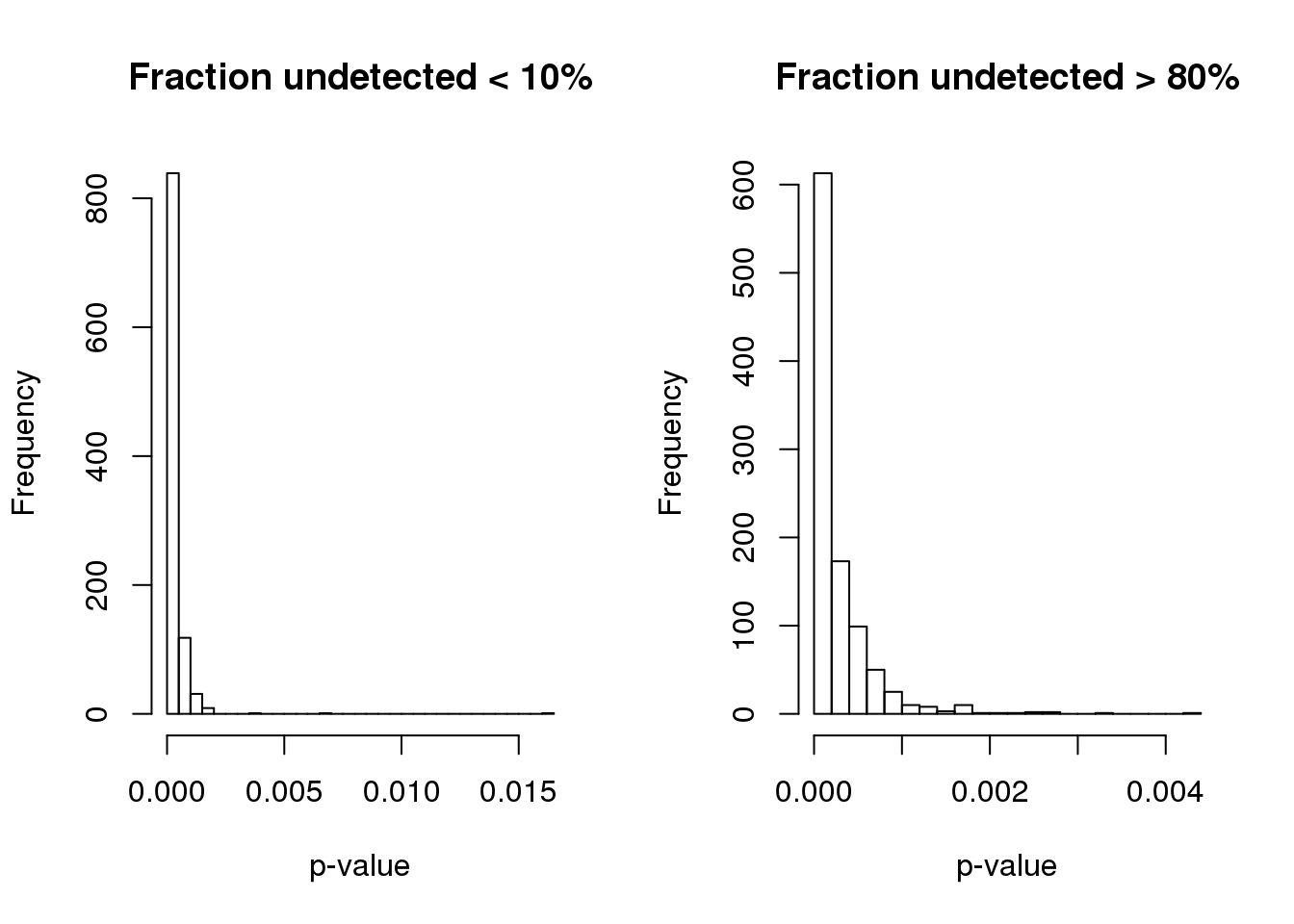

Supplement: Supplemental Material [file supp_gr.247759.118_Supplemental_peco-paper-master-source-code.tar.gz › peco-paper-master/docs/figure/npreg_trendfilter_quantile.Rmd/load-permdist-results-1.png]

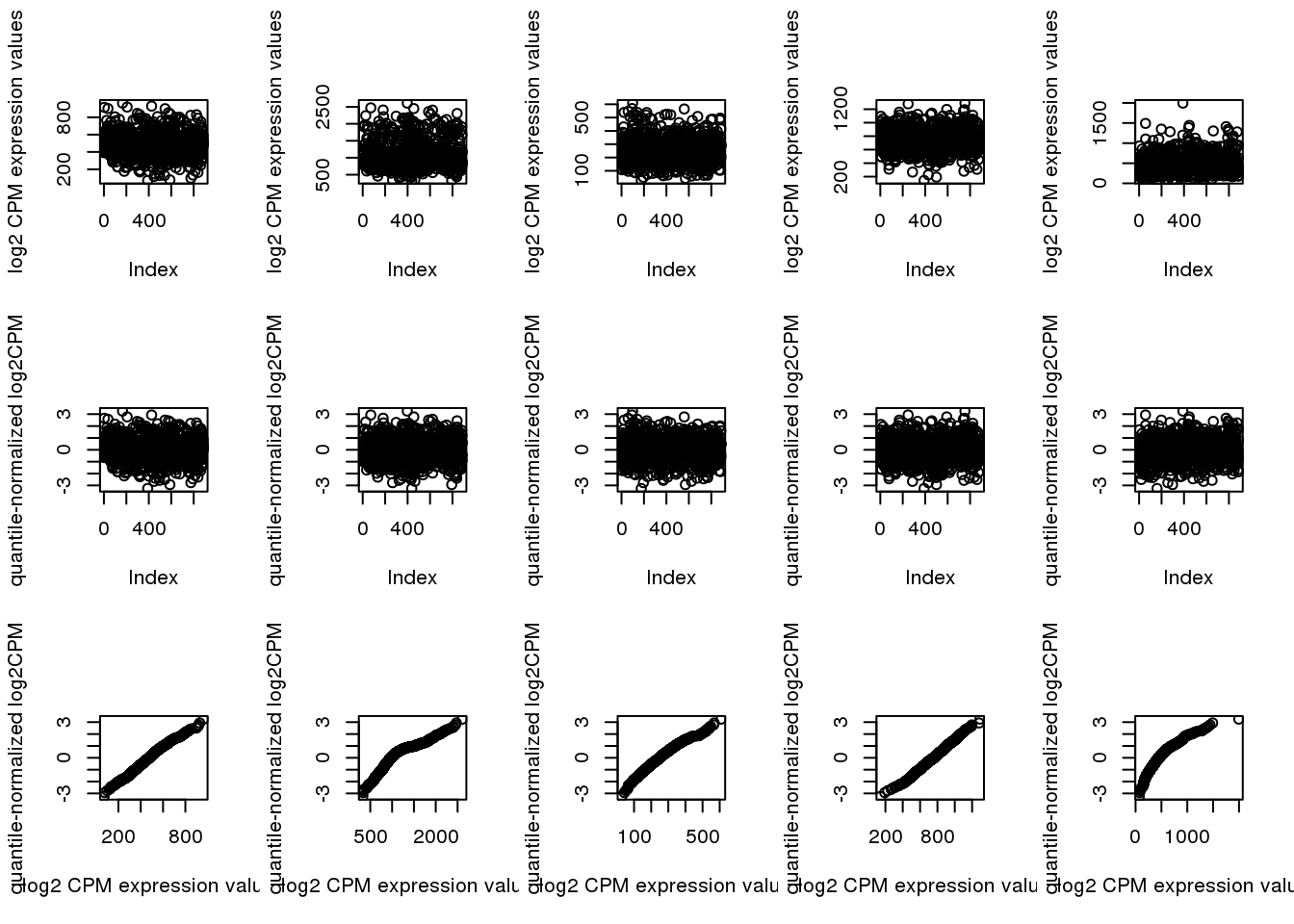

Supplement: Supplemental Material [file supp_gr.247759.118_Supplemental_peco-paper-master-source-code.tar.gz › peco-paper-master/docs/figure/npreg_trendfilter_quantile.Rmd/check-high-low-undetected-cells-2.png]

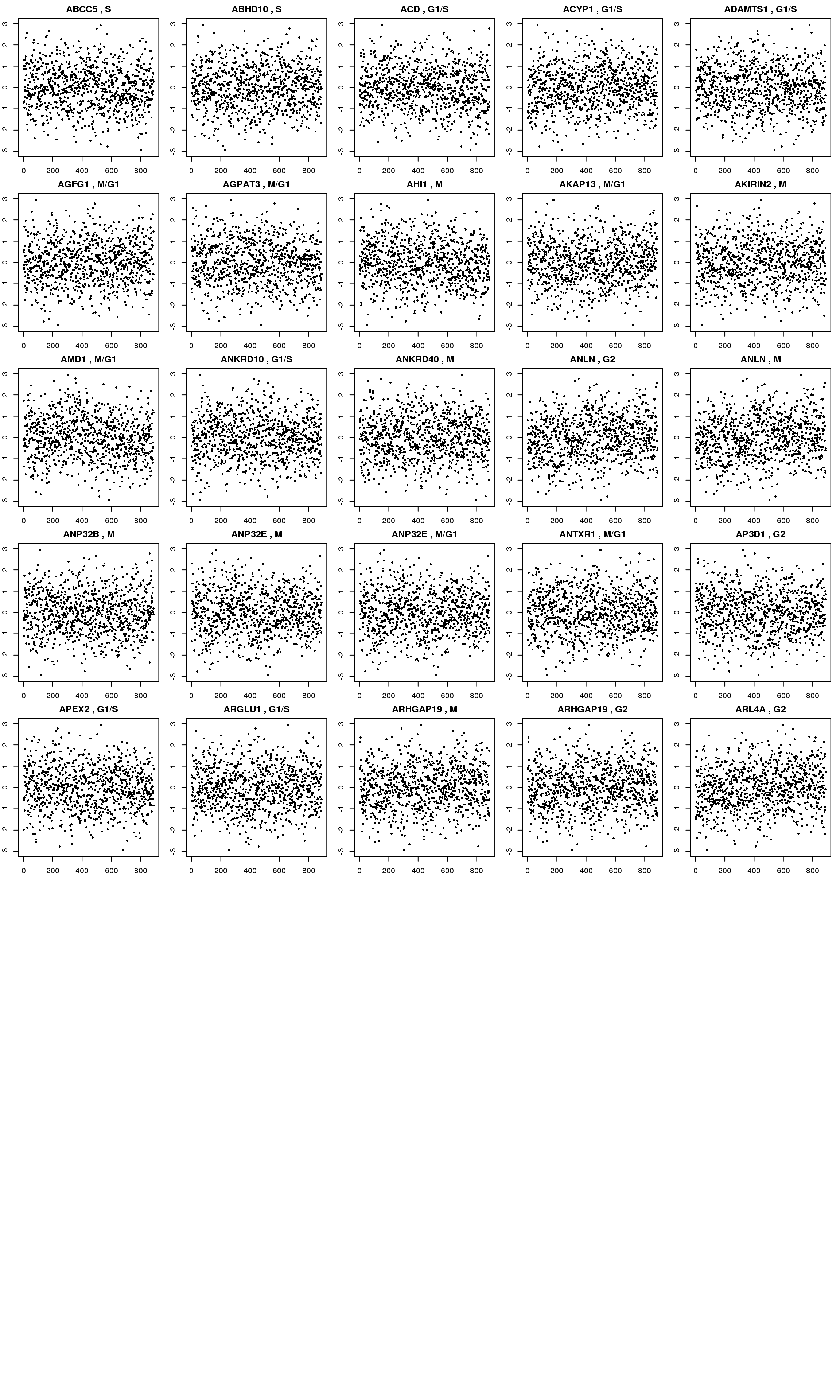

Supplement: Supplemental Material [file supp_gr.247759.118_Supplemental_peco-paper-master-source-code.tar.gz › peco-paper-master/docs/figure/npreg_trendfilter_quantile.Rmd/check-cyclic-1.png]

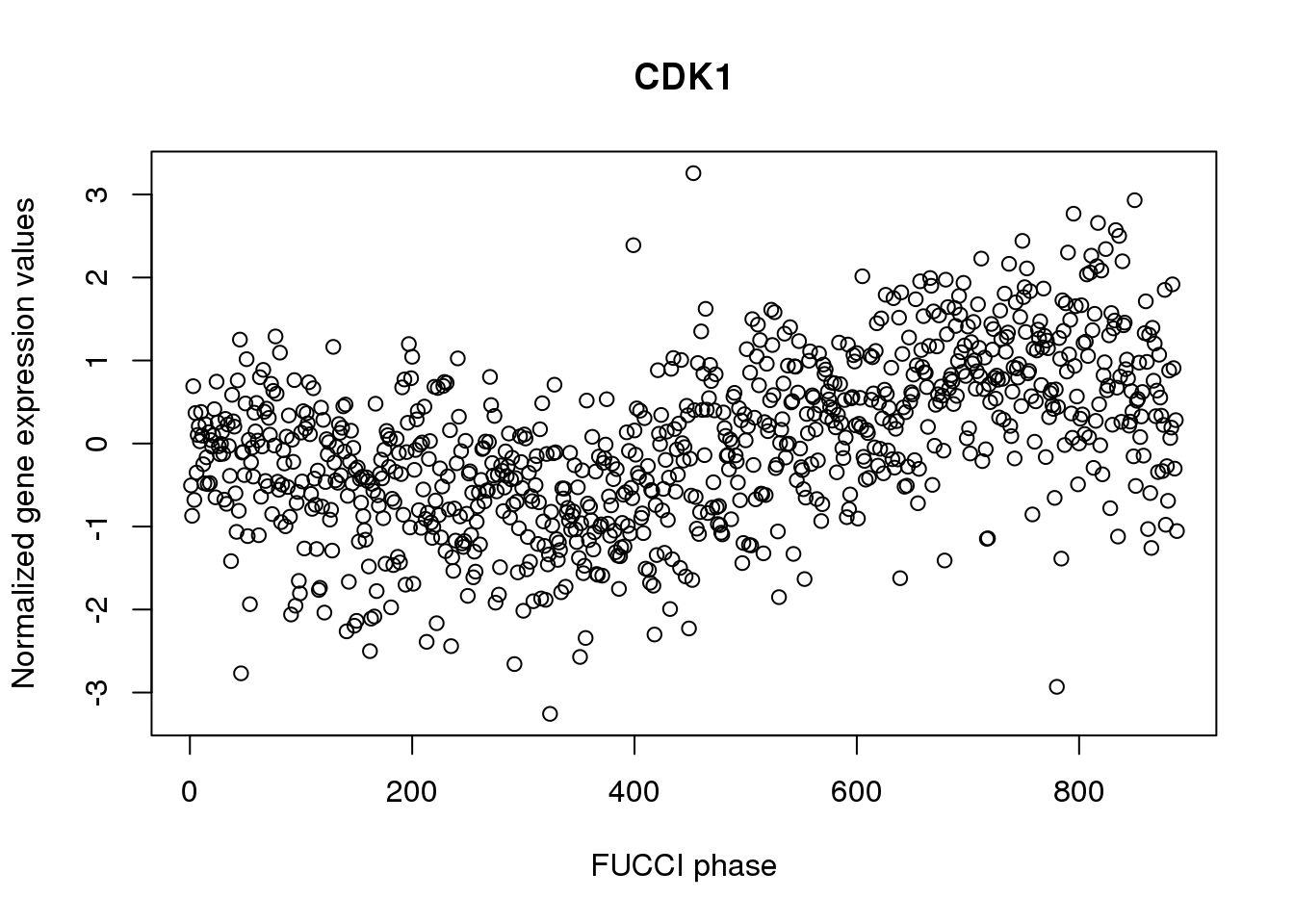

Supplement: Supplemental Material [file supp_gr.247759.118_Supplemental_peco-paper-master-source-code.tar.gz › peco-paper-master/docs/figure/npreg_trendfilter_quantile.Rmd/load-data-1.png]

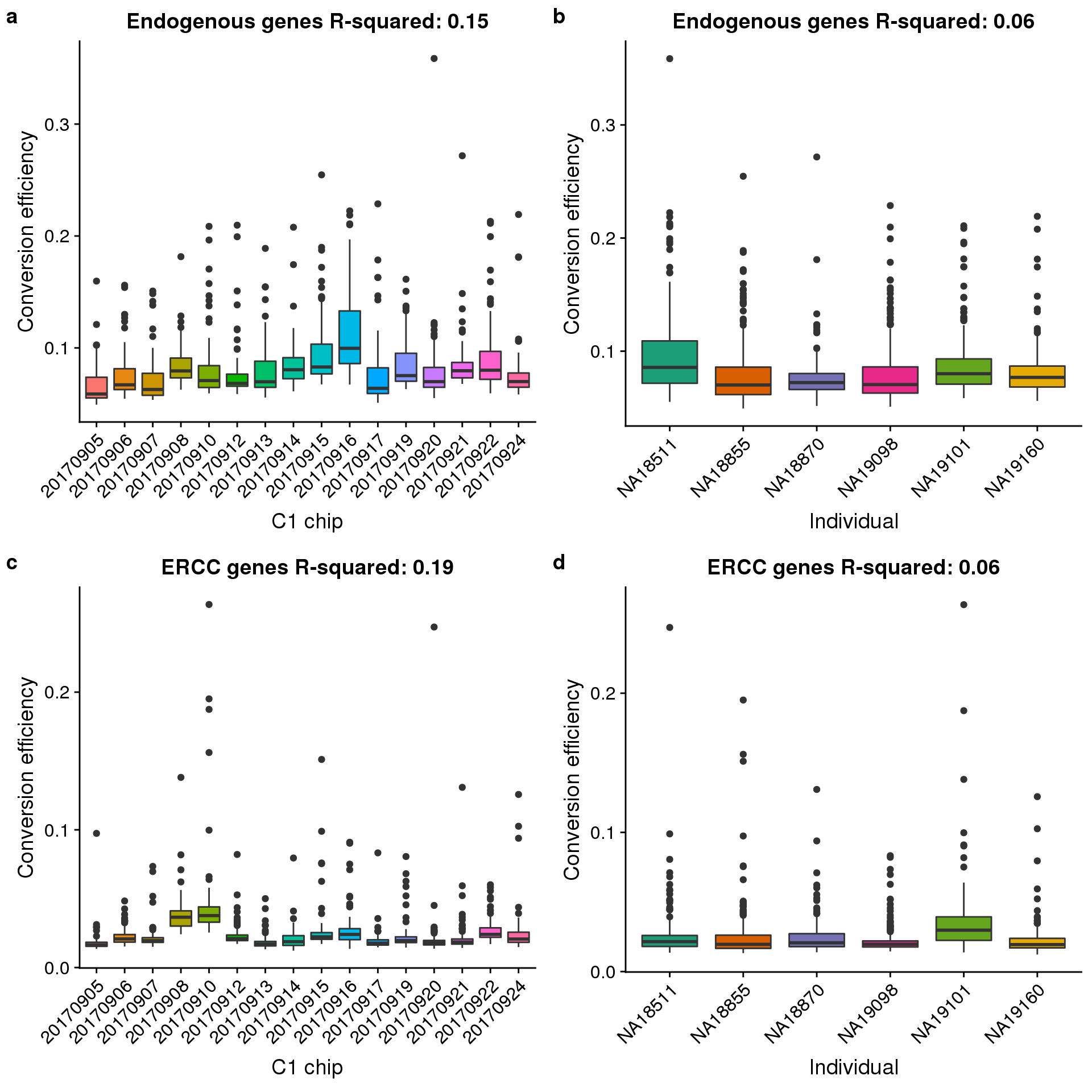

Supplement: Supplemental Material [file supp_gr.247759.118_Supplemental_peco-paper-master-source-code.tar.gz › peco-paper-master/docs/figure/reads-v-molecules.Rmd/conversion-efficiency-1.png]

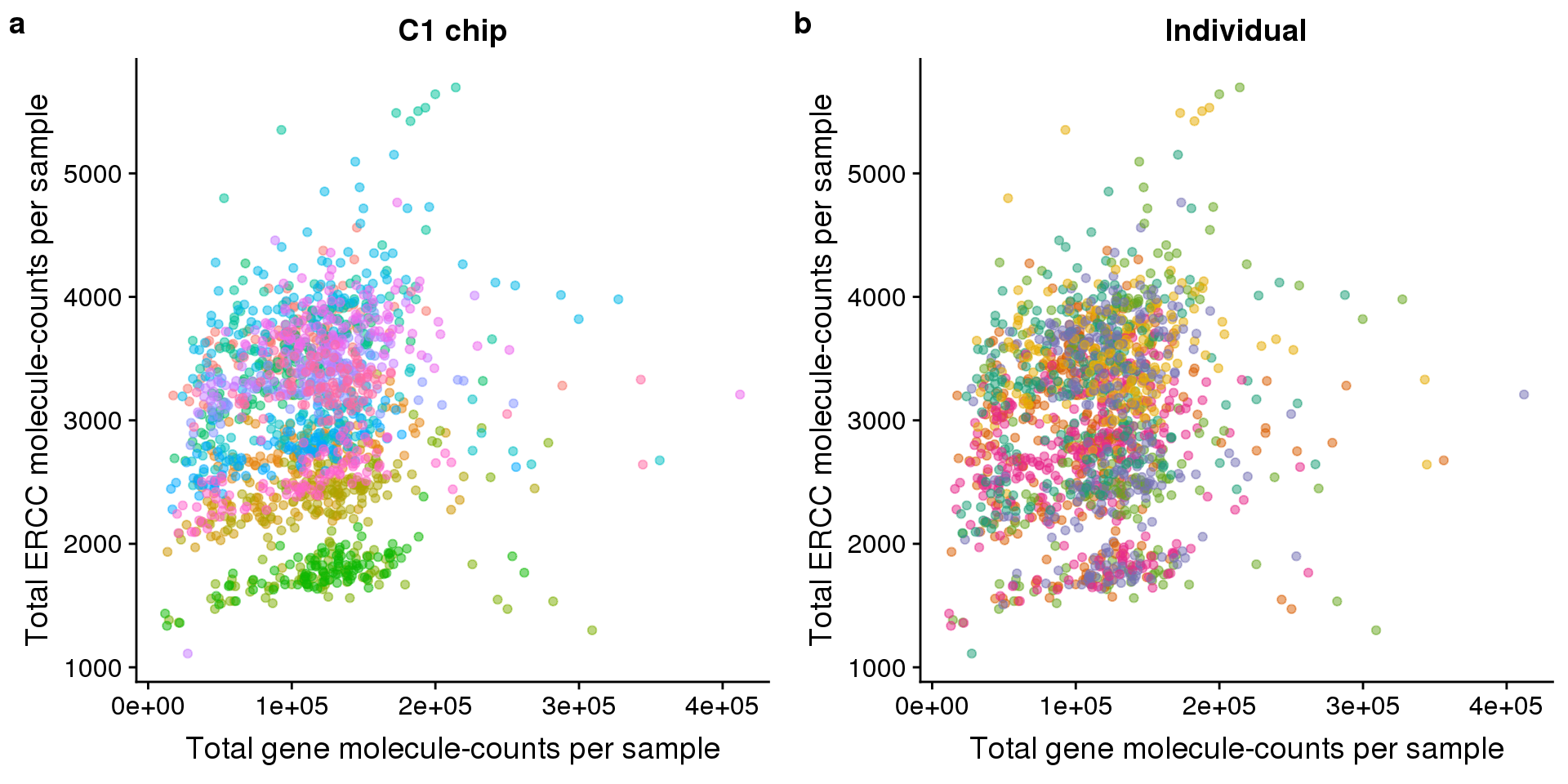

Supplement: Supplemental Material [file supp_gr.247759.118_Supplemental_peco-paper-master-source-code.tar.gz › peco-paper-master/docs/figure/reads-v-molecules.Rmd/gene-v-ercc-1.png]

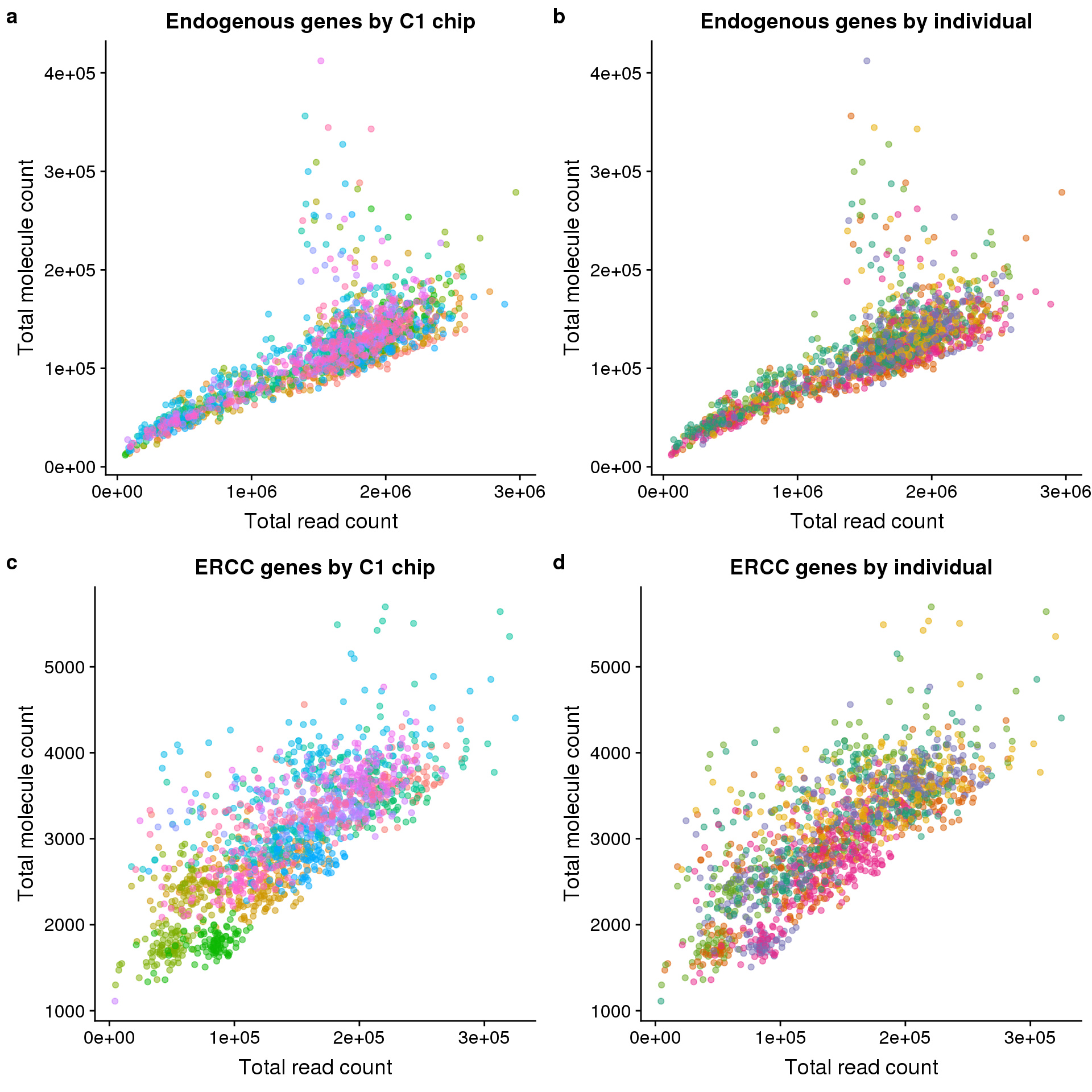

Supplement: Supplemental Material [file supp_gr.247759.118_Supplemental_peco-paper-master-source-code.tar.gz › peco-paper-master/docs/figure/reads-v-molecules.Rmd/reads-v-molecules-1.png]

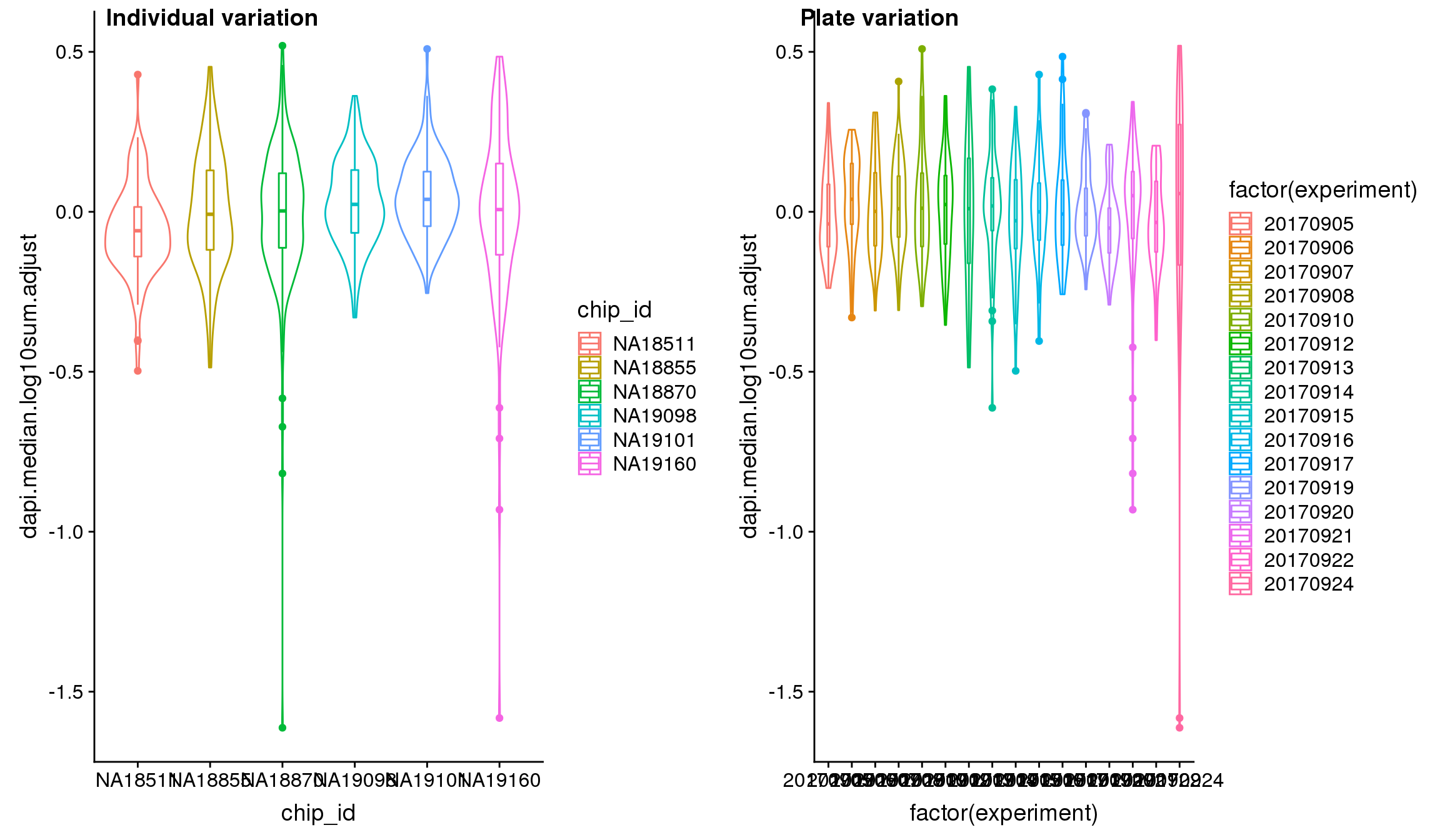

Supplement: Supplemental Material [file supp_gr.247759.118_Supplemental_peco-paper-master-source-code.tar.gz › peco-paper-master/docs/figure/images_batchcorrect.Rmd/boxplot-adjusted-6.png]

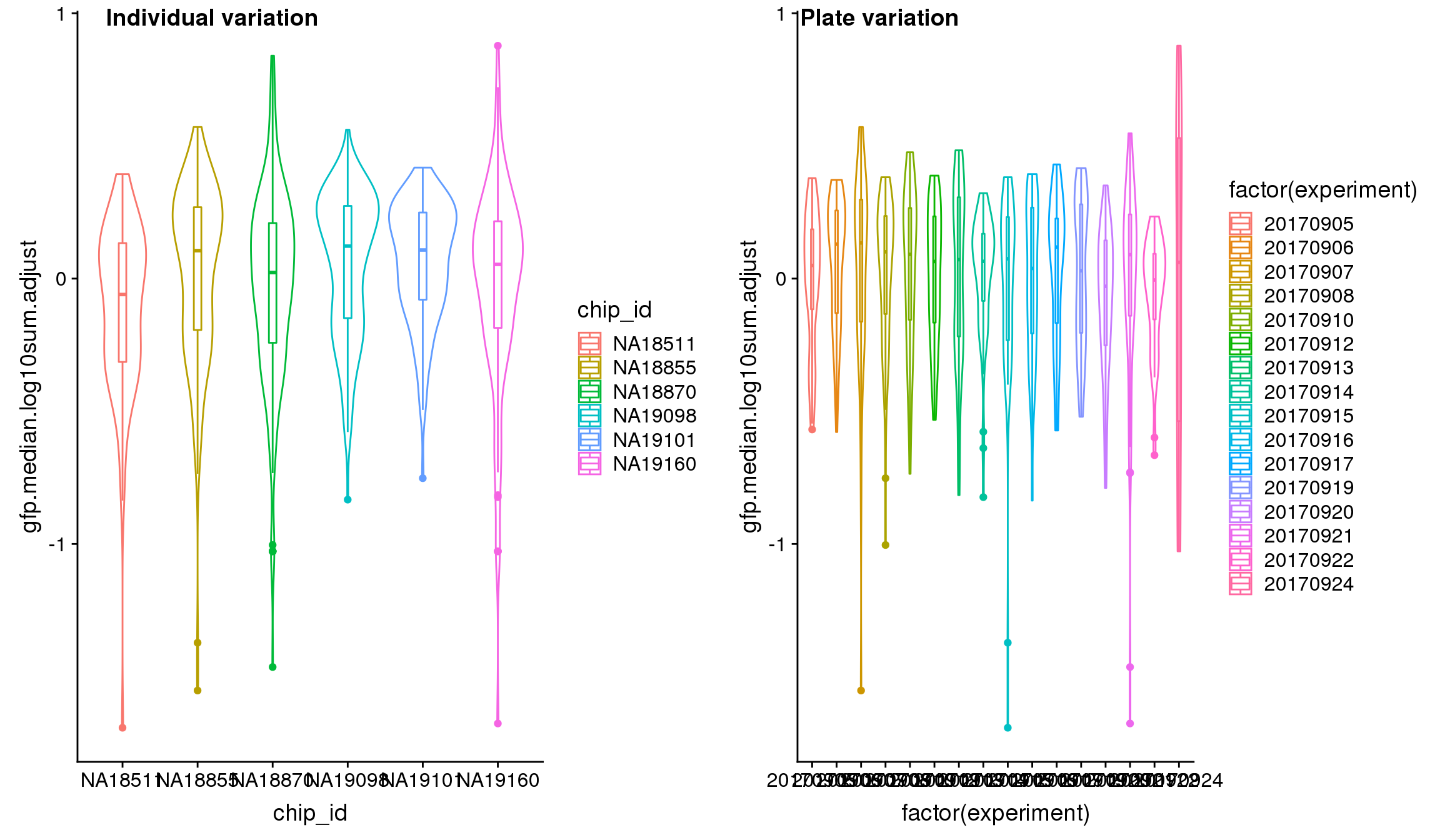

Supplement: Supplemental Material [file supp_gr.247759.118_Supplemental_peco-paper-master-source-code.tar.gz › peco-paper-master/docs/figure/images_batchcorrect.Rmd/boxplot-adjusted-4.png]

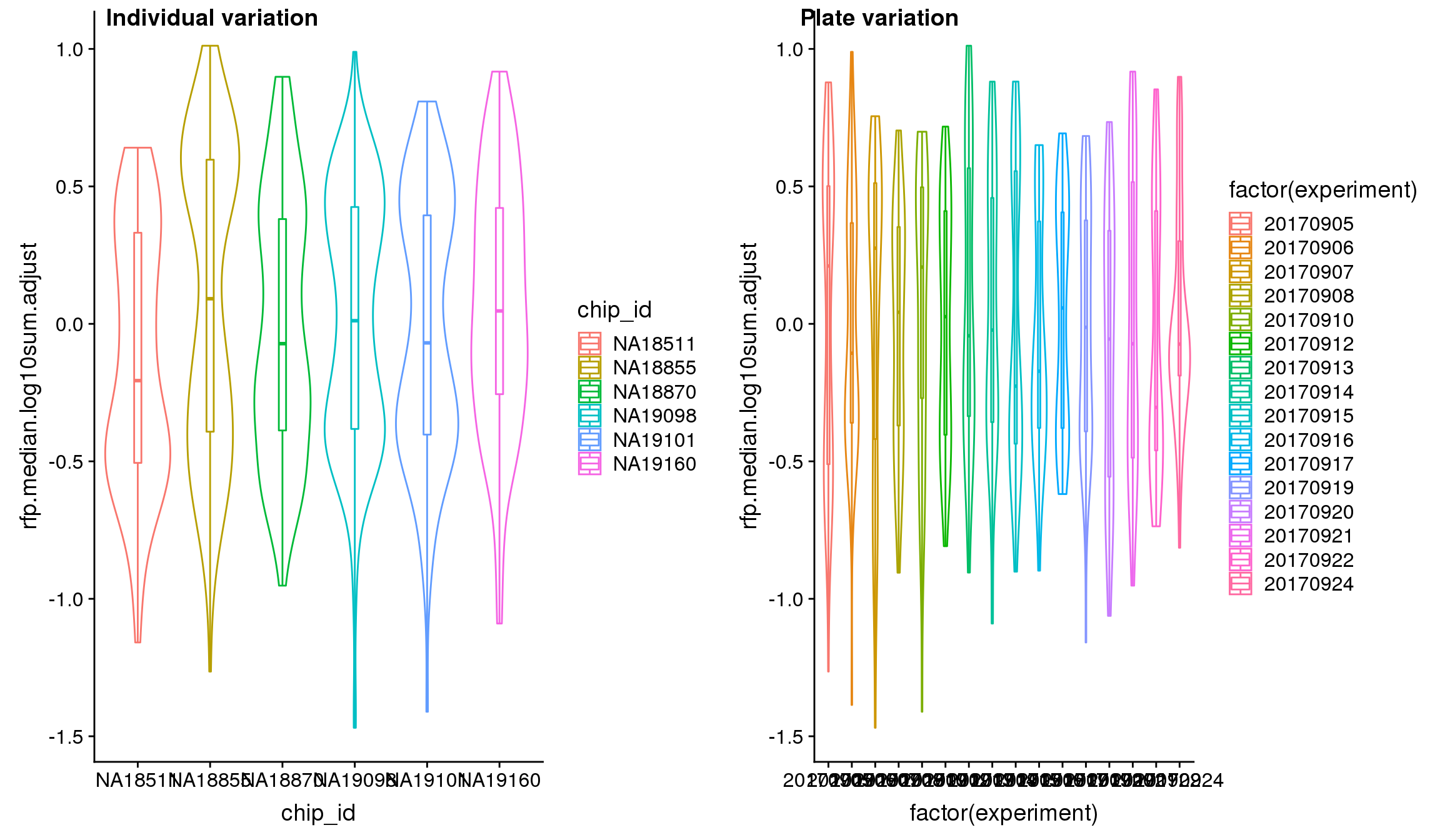

Supplement: Supplemental Material [file supp_gr.247759.118_Supplemental_peco-paper-master-source-code.tar.gz › peco-paper-master/docs/figure/images_batchcorrect.Rmd/boxplot-adjusted-5.png]

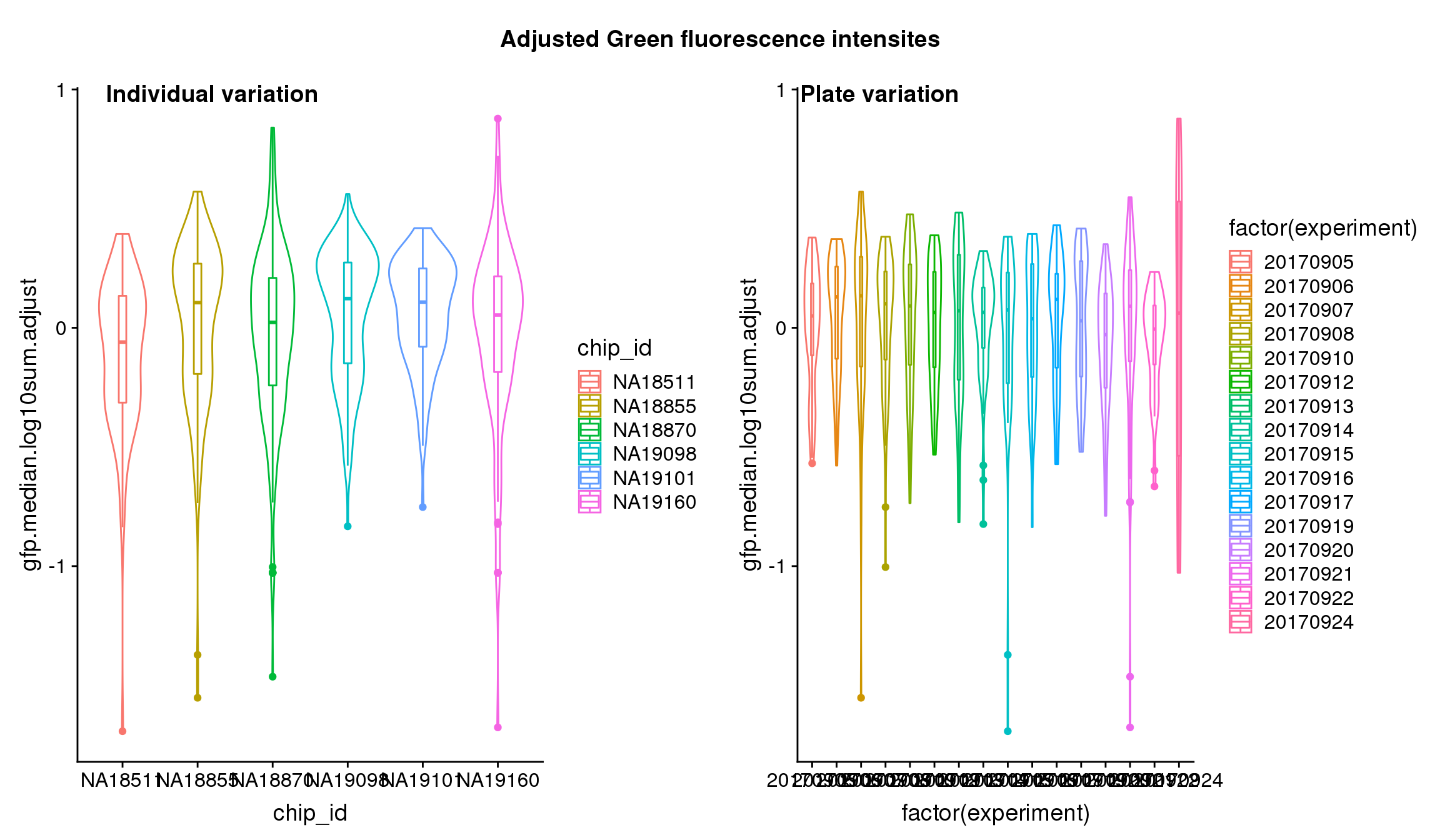

Supplement: Supplemental Material [file supp_gr.247759.118_Supplemental_peco-paper-master-source-code.tar.gz › peco-paper-master/docs/figure/images_batchcorrect.Rmd/boxplot-adjusted-1.png]

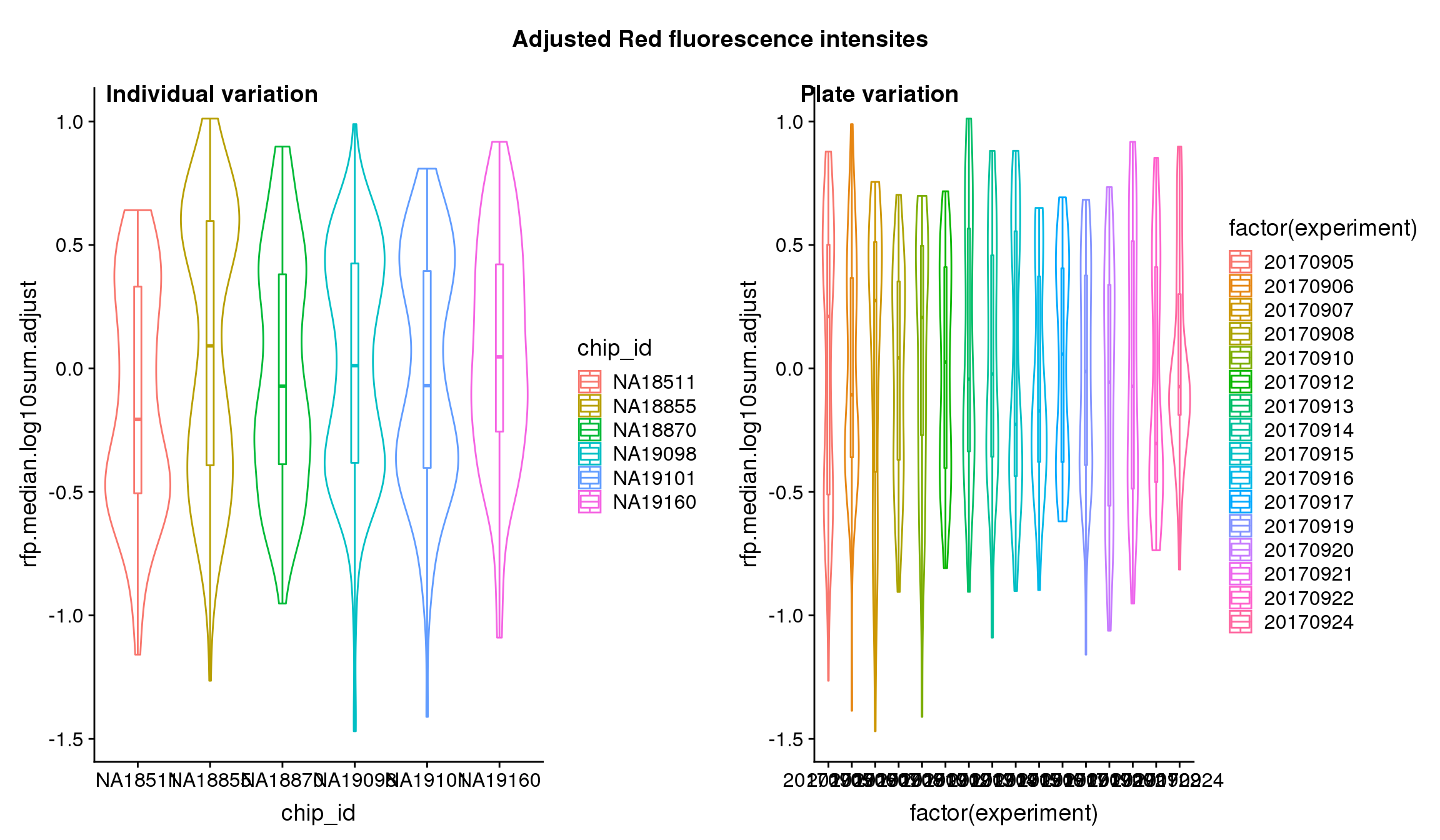

Supplement: Supplemental Material [file supp_gr.247759.118_Supplemental_peco-paper-master-source-code.tar.gz › peco-paper-master/docs/figure/images_batchcorrect.Rmd/boxplot-adjusted-2.png]

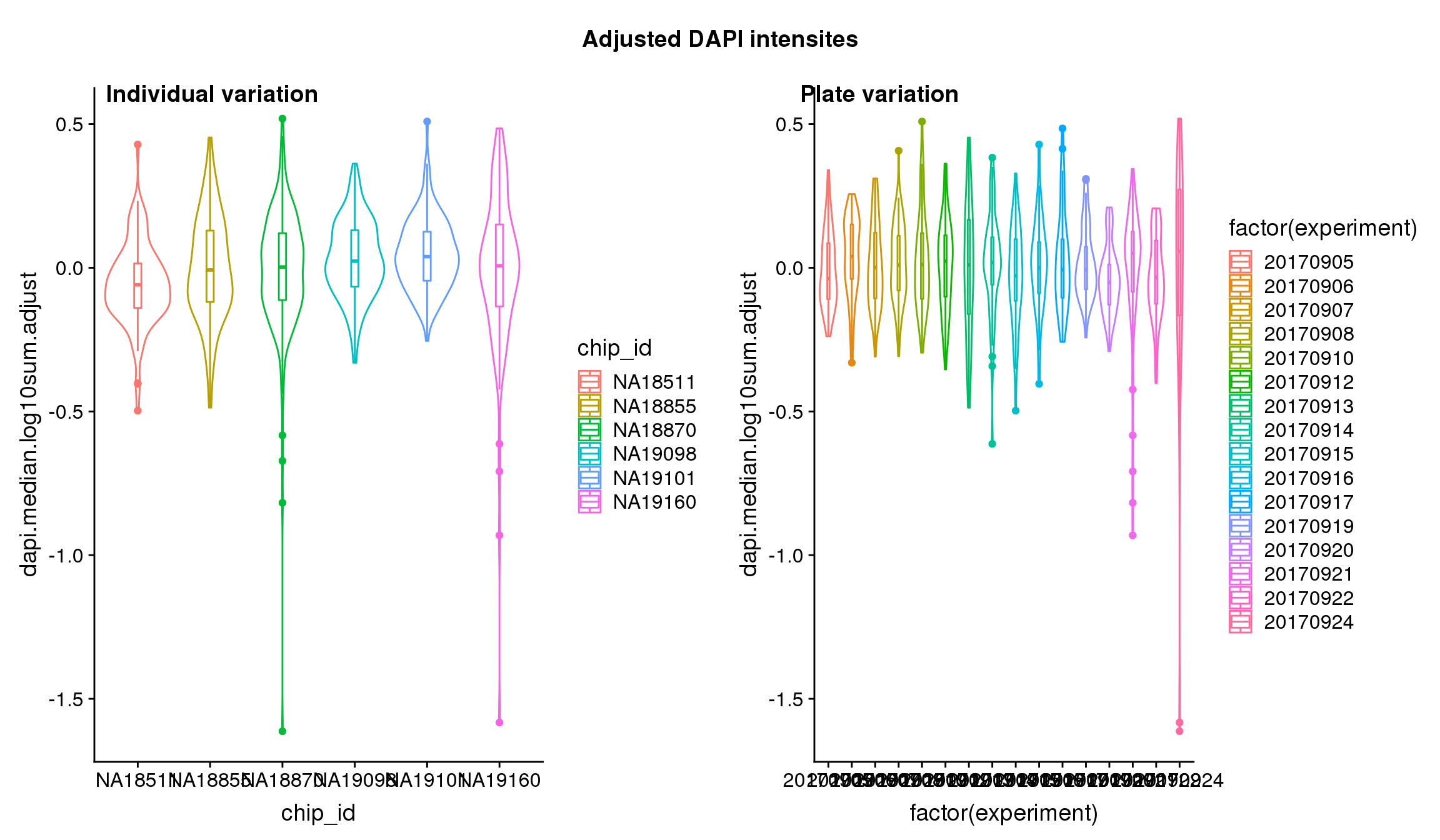

Supplement: Supplemental Material [file supp_gr.247759.118_Supplemental_peco-paper-master-source-code.tar.gz › peco-paper-master/docs/figure/images_batchcorrect.Rmd/boxplot-adjusted-3.png]

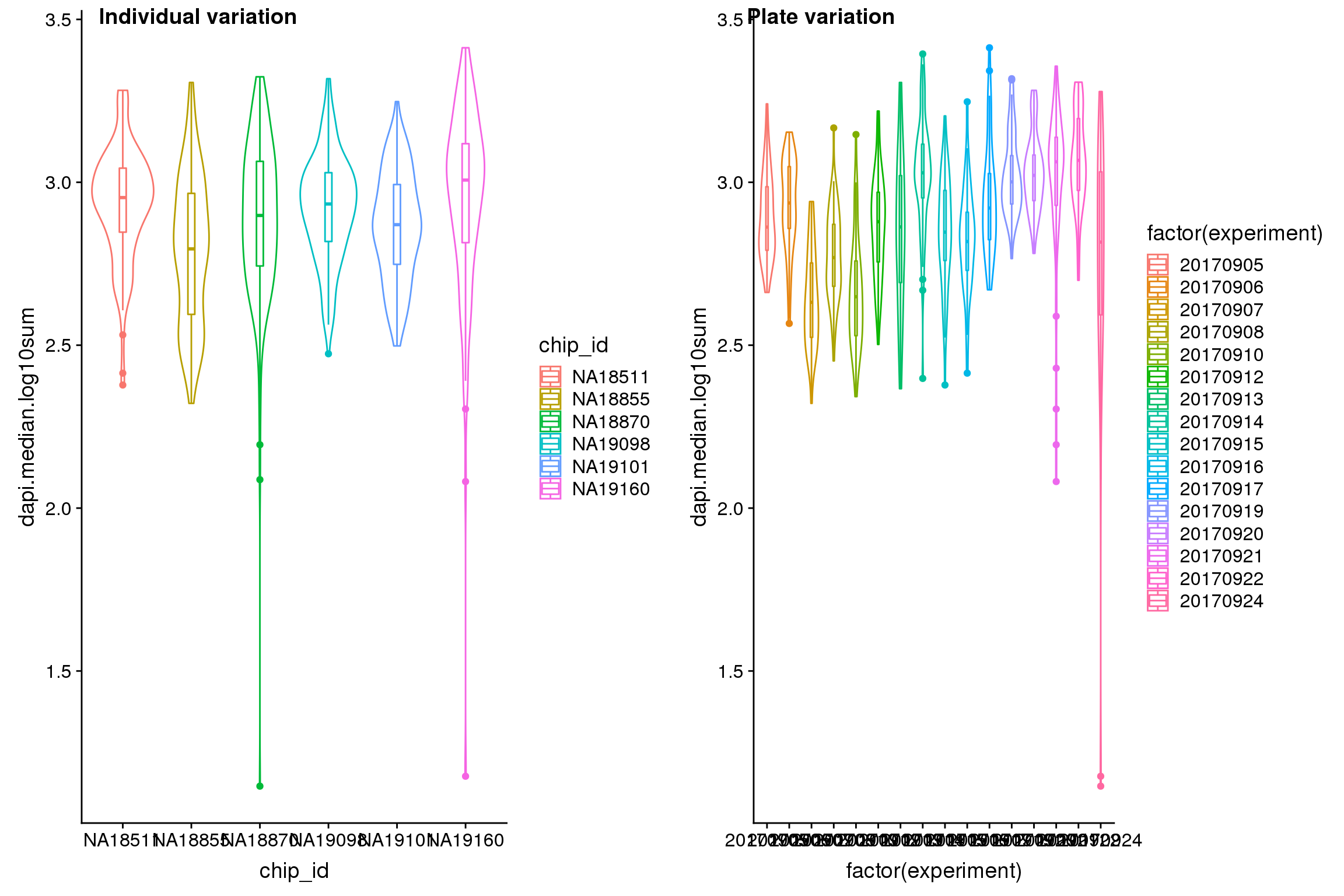

Supplement: Supplemental Material [file supp_gr.247759.118_Supplemental_peco-paper-master-source-code.tar.gz › peco-paper-master/docs/figure/images_batchcorrect.Rmd/unnamed-chunk-4-6.png]

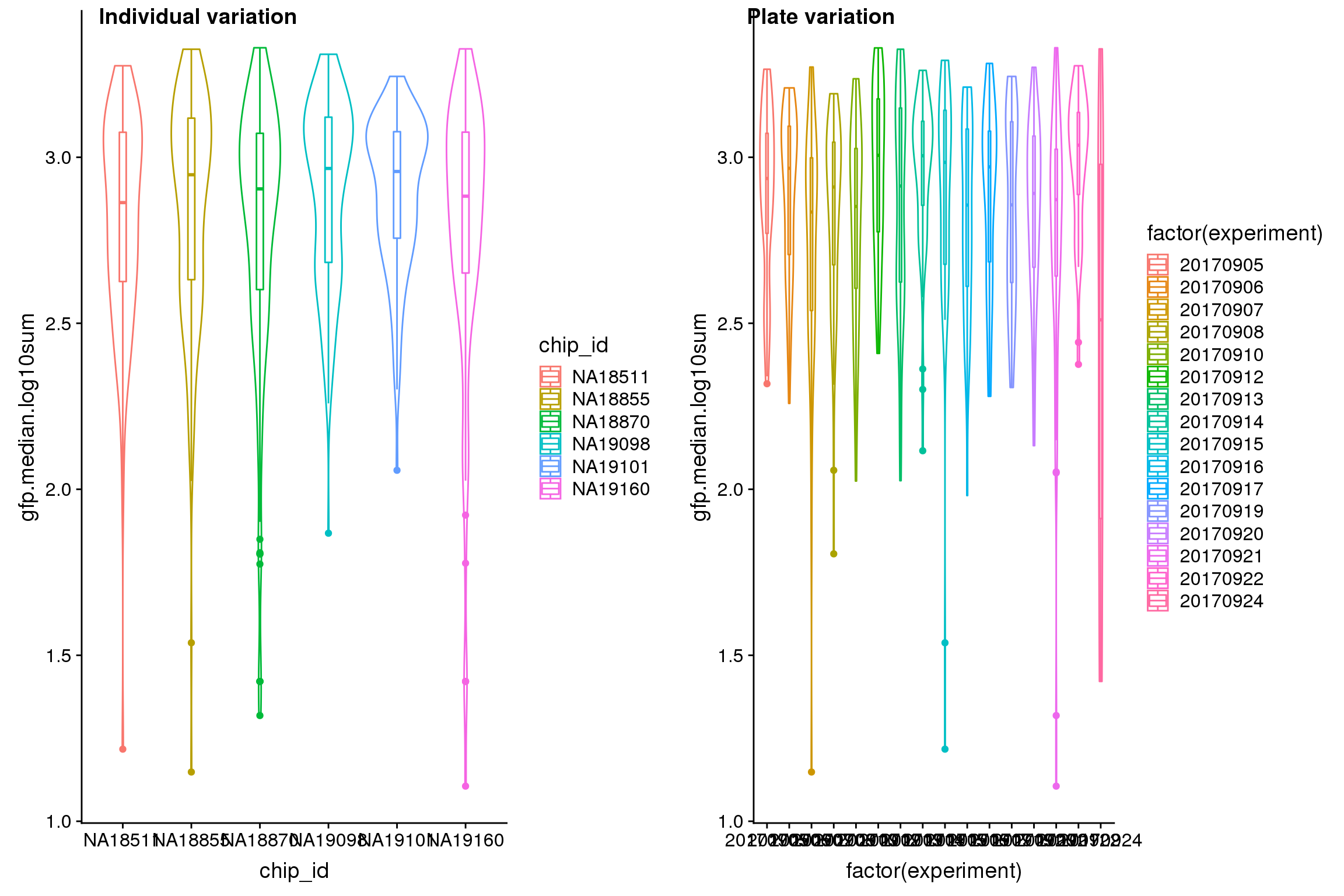

Supplement: Supplemental Material [file supp_gr.247759.118_Supplemental_peco-paper-master-source-code.tar.gz › peco-paper-master/docs/figure/images_batchcorrect.Rmd/unnamed-chunk-4-4.png]

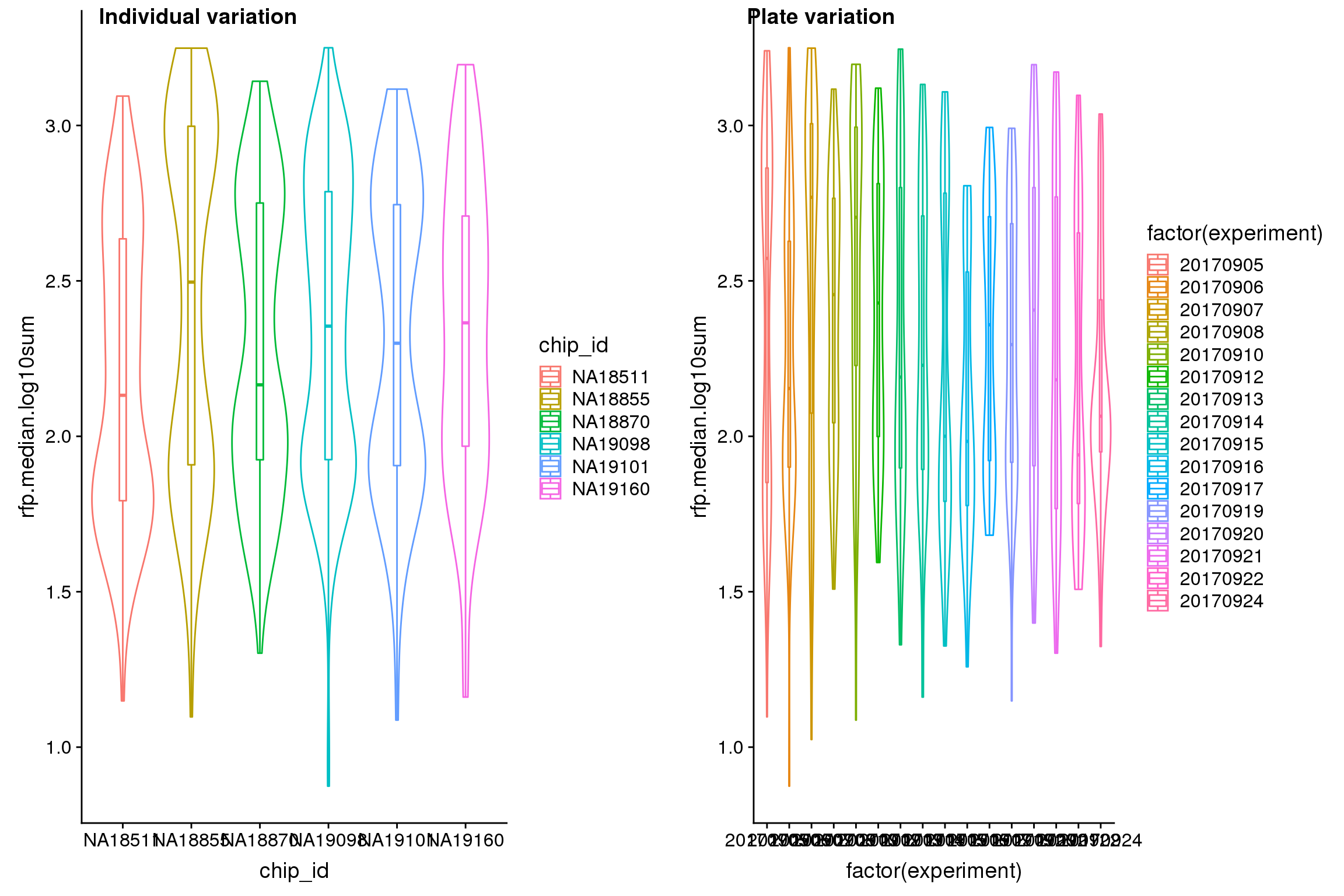

Supplement: Supplemental Material [file supp_gr.247759.118_Supplemental_peco-paper-master-source-code.tar.gz › peco-paper-master/docs/figure/images_batchcorrect.Rmd/unnamed-chunk-4-5.png]

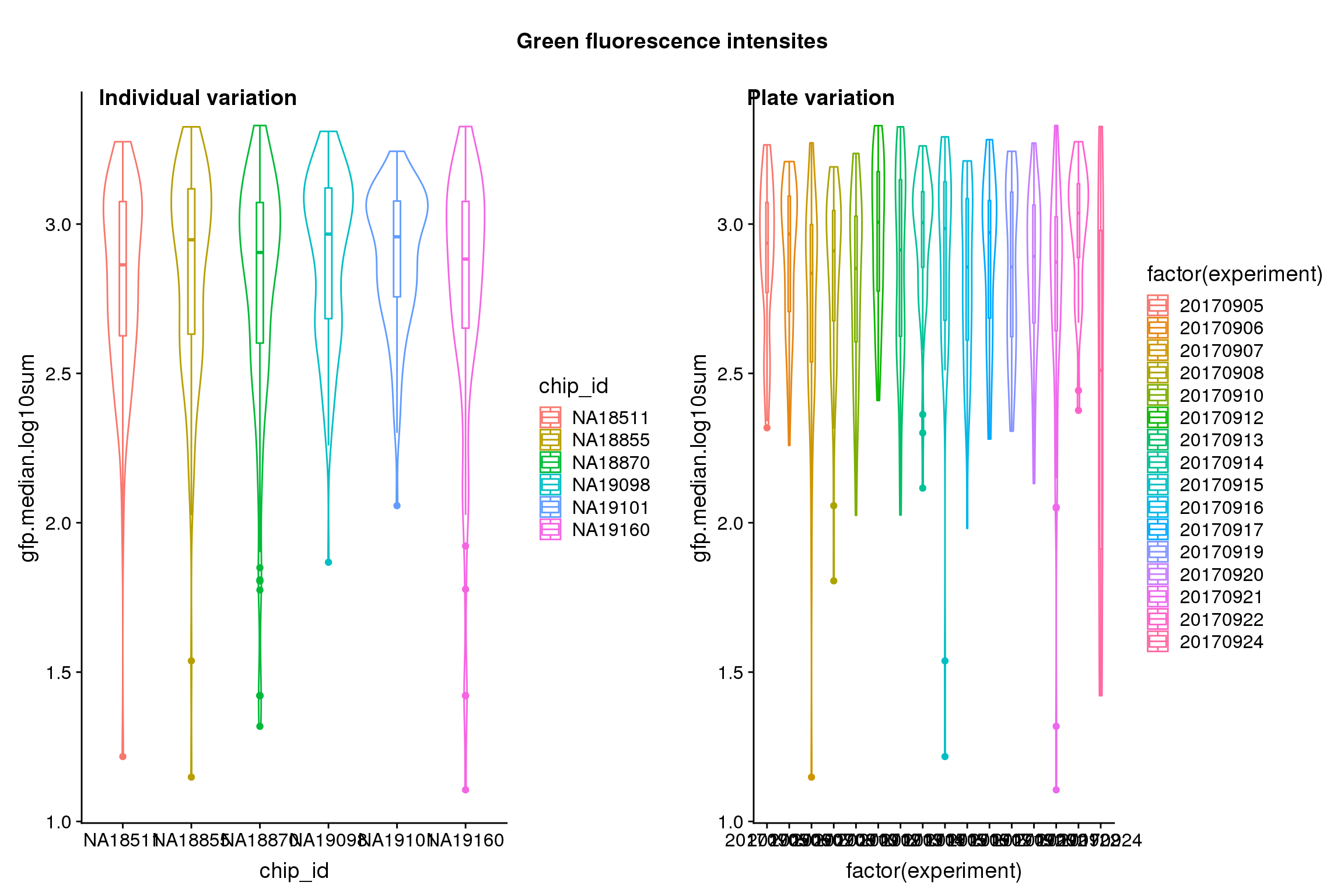

Supplement: Supplemental Material [file supp_gr.247759.118_Supplemental_peco-paper-master-source-code.tar.gz › peco-paper-master/docs/figure/images_batchcorrect.Rmd/unnamed-chunk-4-1.png]

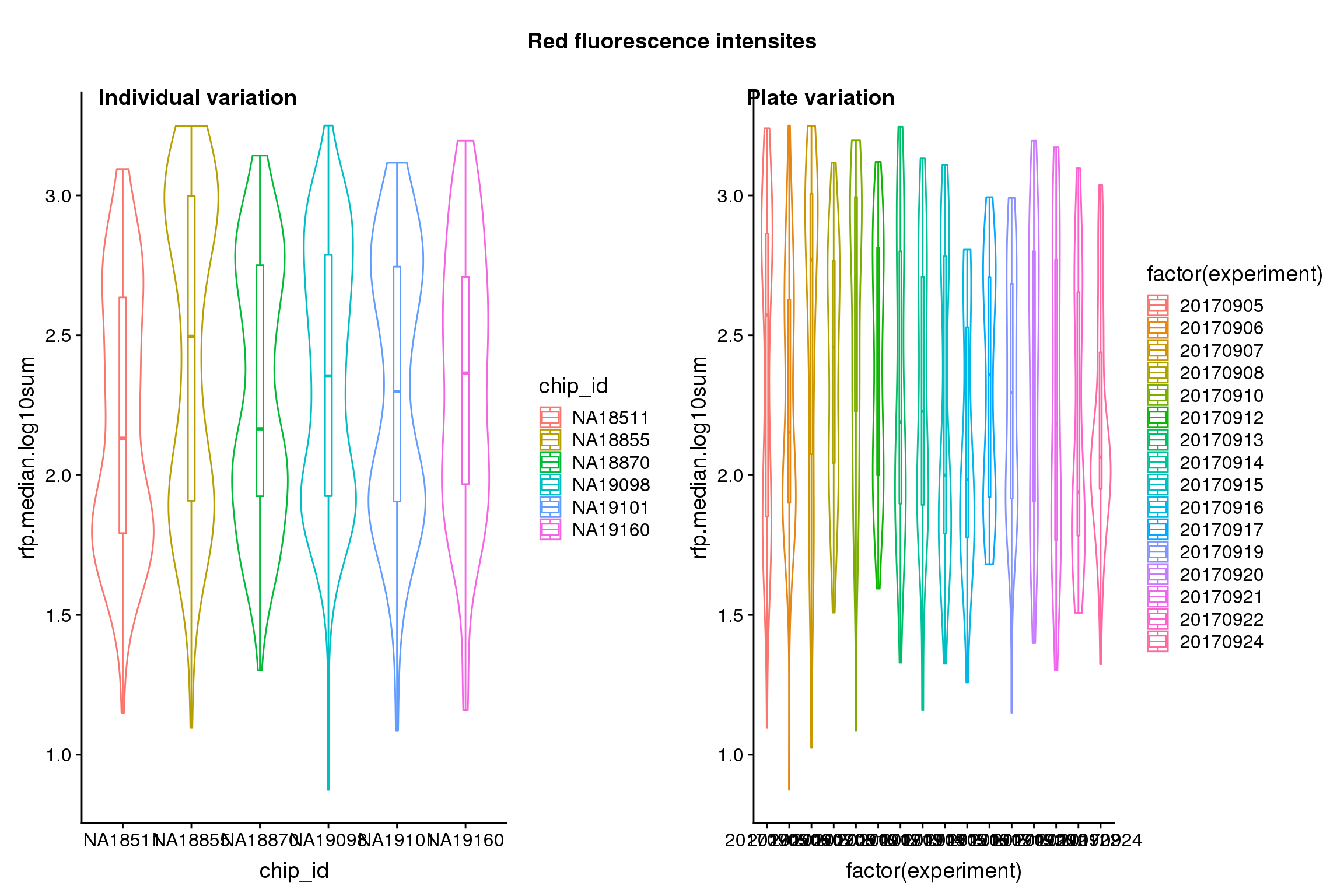

Supplement: Supplemental Material [file supp_gr.247759.118_Supplemental_peco-paper-master-source-code.tar.gz › peco-paper-master/docs/figure/images_batchcorrect.Rmd/unnamed-chunk-4-2.png]

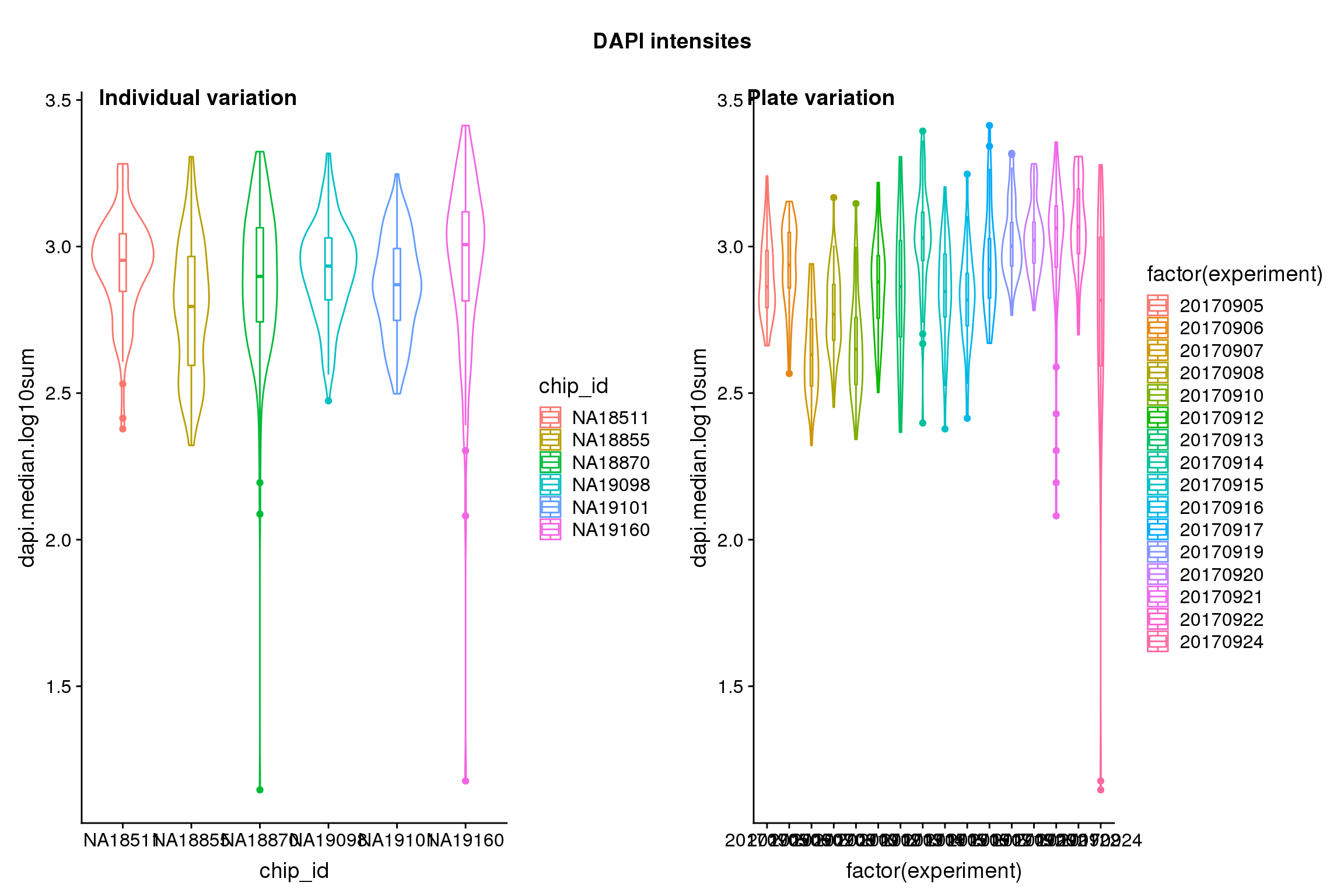

Supplement: Supplemental Material [file supp_gr.247759.118_Supplemental_peco-paper-master-source-code.tar.gz › peco-paper-master/docs/figure/images_batchcorrect.Rmd/unnamed-chunk-4-3.png]

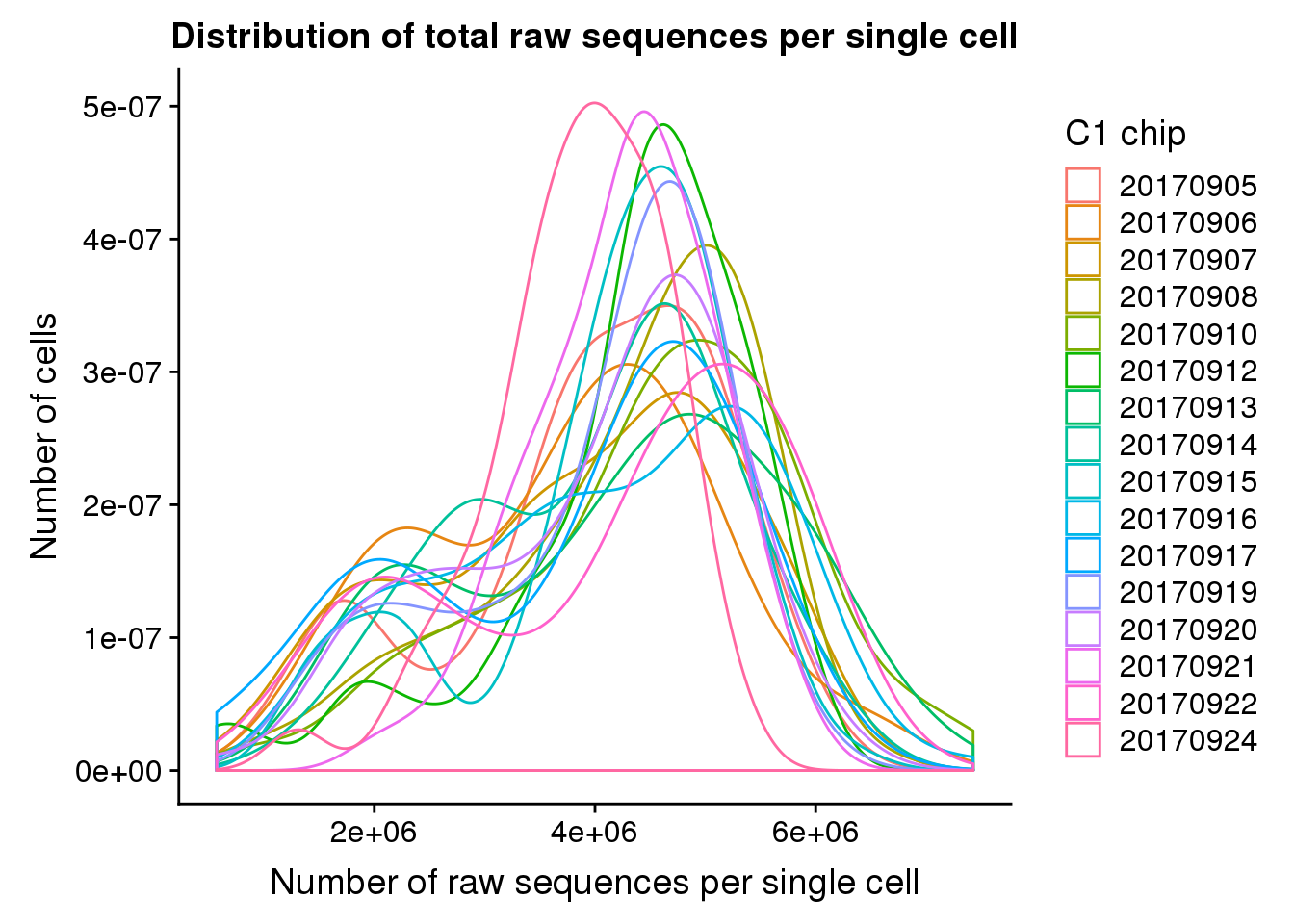

Supplement: Supplemental Material [file supp_gr.247759.118_Supplemental_peco-paper-master-source-code.tar.gz › peco-paper-master/docs/figure/totals.Rmd/distribution-sequencing-depth-1.png]

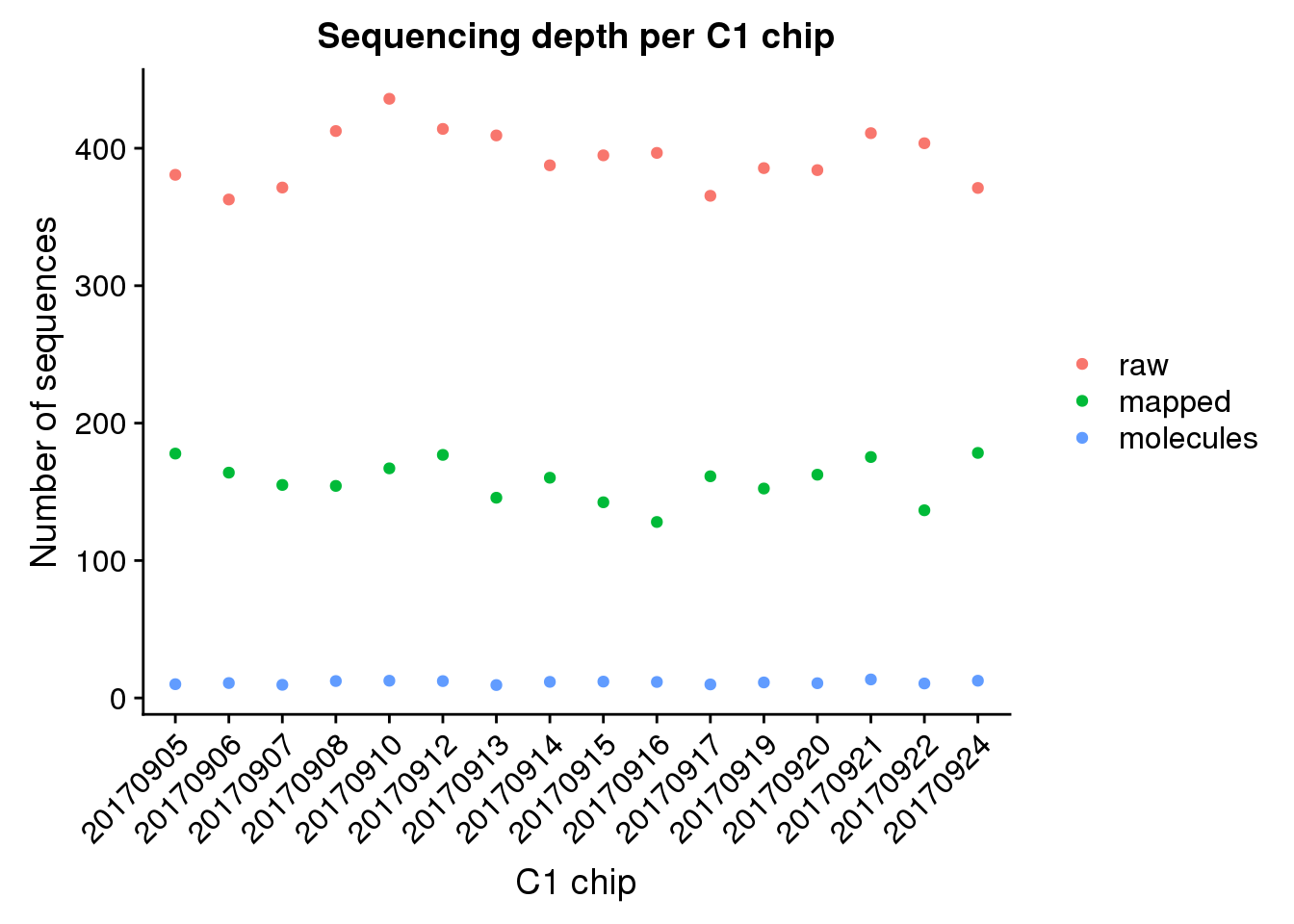

Supplement: Supplemental Material [file supp_gr.247759.118_Supplemental_peco-paper-master-source-code.tar.gz › peco-paper-master/docs/figure/totals.Rmd/unnamed-chunk-1-1.png]

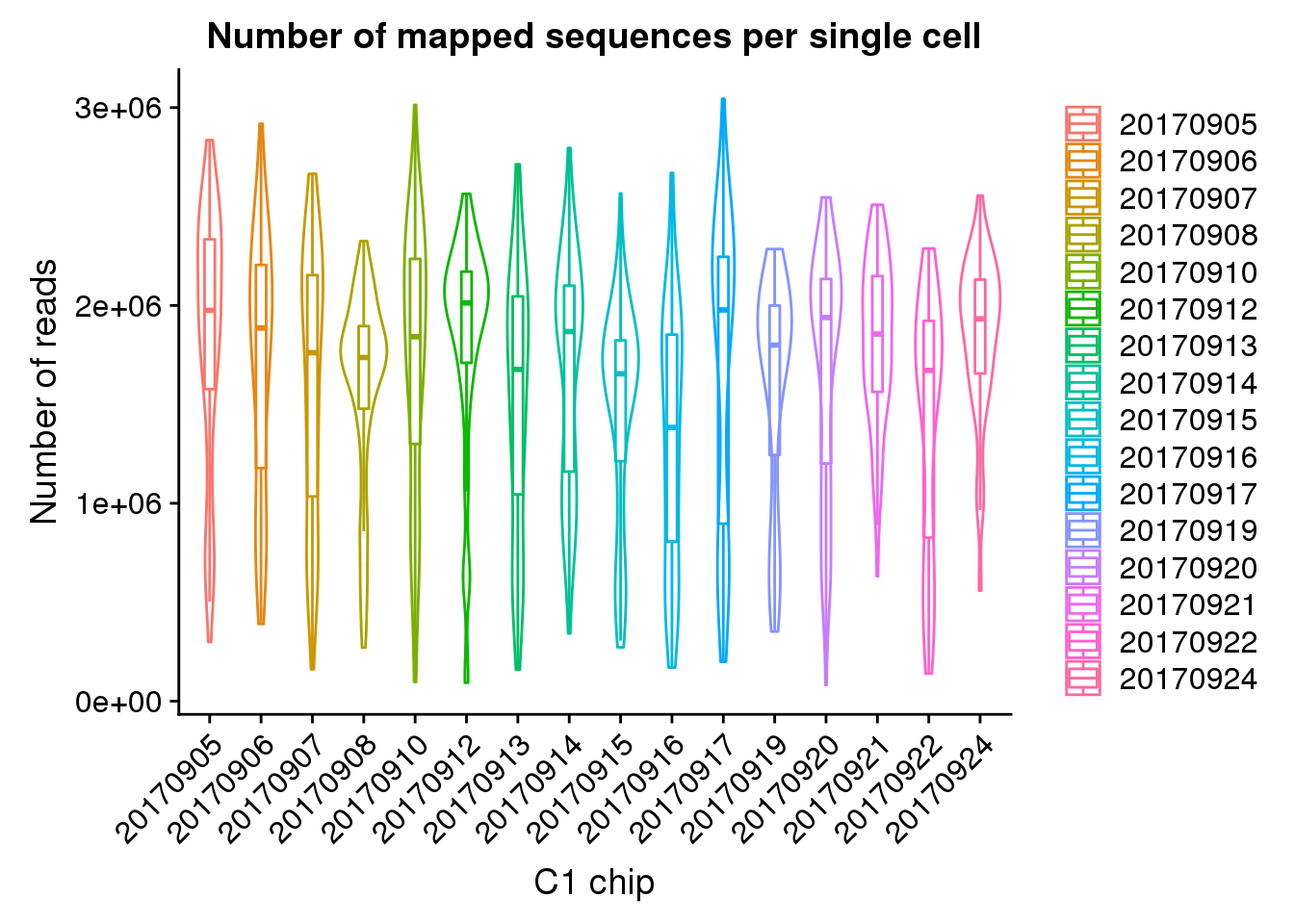

Supplement: Supplemental Material [file supp_gr.247759.118_Supplemental_peco-paper-master-source-code.tar.gz › peco-paper-master/docs/figure/totals.Rmd/mapped-1.png]
